# Supplementary material for: Safety and continued use of the levonorgestrel intrauterine system as compared with the copper intrauterine device among women living with HIV in South Africa: A randomized controlled trial
Source: PLoS Med. 2020 May 22;17(5):e1003110. doi: 10.1371/journal.pmed.1003110 (PMC7244096; doi:10.1371/journal.pmed.1003110)
Supplement: S1 Table — ART, antiretroviral therapy; C-IUD, copper T-380 intrauterine device; LNG-IUS, levonorgestrel intrauterine system (DOCX) [file pmed.1003110.s002.docx]

|  | **All participants (n= 199)** | **ART-using women (n= 132)** | **Non-ART women (n= 67)** |
| --- | --- | --- | --- |
| **Detectable gVL by study visit** | **OR (95% CI)** | **OR (95% CI)** | **OR (95% CI)** |
|  |  |  |  |
| **As-treated analysis** |  |  |  |
| Across 6 months | 0·83 (0·46–1·48) | 0·84 (0·39–1·82) | 0·81 (0·33–1·97) |
| Across 24 months | 0·98 (0·64 – 1·50) | 0·78 (0·46 – 1·34) | 1·30 (0·65 – 2·62) |
| **Intent-to-treat analysis** |  |  |  |
| Across 6 months | 0·87 (0·50–1·53) | 0·75 (0·36–1·58) | 1·07 (0·44–2·60) |
| Across 24 months | 1·02 (0·68–1·52) | 0·72 (0·43–1·22) | 1·53 (0·79–2·94) |
| **Adjusted as-treated analysis** |  |  |  |
| Across 6 months (covariate set #1) | 0·78 (0·44–1·38) | 0·81 (0·38–1·75) | 0·72 (0·30–1·73) |
| Across 6 months (covariate set #5) | 0·89 (0·49–1·60) | 0·84 (0·40–1·79) |  |
| Across 24 months (covariate set #1) | 0·98 (0·64–1·49) | 0·78 (0·45–1·33) | 1·30 (0·65–2·62) |
| Across 24 months (covariate set #5) | 1·04 (0·69–1·58) | 0·79 (0·48–1·32) | 1·33 (0·61–2·93) |
| ART=antiretroviral therapy; CI=confidence interval; n=number; OR=odds ratio; pVL= plasma VL; RTI=reproductive tract infection.  Covariate set in as-treated and intent-to-treat population: baseline detectable genital VL, age, and ART group (combined only). Covariate set #1: Baseline detectable gVL, any RTI, age, and ART group (combined only). Covariate set #5: Baseline detectable gVL, any RTI, age, baseline pVL (dichotomous), pVL (log 10 continuous), and ART group (combined only). | | | |

**S1_Table. Odds of detectable genital tract viral load comparing women using the levonorgestrel intrauterine system (LNG-IUS) with those using the copper T-380 intrauterine device (C-IUD) with linear regression weighted for differential intrauterine contraceptive discontinuation rates, stratified by ART status, among women living with HIV in Cape Town, South Africa.**
