# Supplementary material for: Safety and continued use of the levonorgestrel intrauterine system as compared with the copper intrauterine device among women living with HIV in South Africa: A randomized controlled trial
Source: PLoS Med. 2020 May 22;17(5):e1003110. doi: 10.1371/journal.pmed.1003110 (PMC7244096; doi:10.1371/journal.pmed.1003110)
Supplement: S2 Table — ART, antiretroviral therapy; C-IUD, copper T-380 intrauterine device; LNG-IUS, levonorgestrel intrauterine system (DOCX) [file pmed.1003110.s003.docx]

**S2 Table. Odds of detectable genital tract viral load comparing women using the levonorgestrel intrauterine system (LNG-IUS) with those using the copper T-380 intrauterine device (C-IUD) with linear regression adjusted by visit month, stratified by ART status, among women living with HIV in Cape Town, South Africa**

|  | **All participants (n= 199)** | **ART-using women (n= 132)** | **Non-ART women (n= 67)** |
| --- | --- | --- | --- |
| **Detectable gVL by study visit** | **OR (95% CI)** | **OR (95% CI)** | **OR (95% CI)** |
| **As-treated analysis** |  |  |  |
| Across 6 months | 0·82 (0·46–1·47) | 0·85 (0·39–1·84) | 0·80 (0·33–1·93) |
| Across 24 months | 0·96 (0·63–1·46) | 0·79 (0·46–1·34) | 1·22 (0·61–2·42) |
| **Intent-to-treat analysis** |  |  |  |
| Across 6 months | 0·87 (0·49–1·52) | 0·76 (0·36–1·60) | 1·04 (0·43–2·53) |
| Across 24 months | 0·99 (0·66–1·48) | 0·74 (0·44–1·25) | 1·41 (0·74–2·68) |
| **Adjusted as-treated analysis** |  |  |  |
| Across 6 months (covariate set #1) | 0·77 (0·43–1·37) | 0·81 (0·38–1·76) | 0·71 (0·30–1·69) |
| Across 6 months (covariate set #5) | 0·88 (0·49–1·60) | 0·83 (0·39–1·78) |  |
| Across 24 months (covariate set #1) | 0·95 (0·63–1·45) | 0·78 (0·46–1·34) | 1·22 (0·61–2·41) |
| Across 24 months (covariate set #5) | 1‡03 (0·68–1·57) | 0·79 (0·48–1·32) | 1·31 (0·60–2·86) |
| ART=antiretroviral therapy; CI=confidence interval; n=number; OR=odds ratio; pVL=plasma viral load; RTI=reproductive tract infection. Covariate set in as-treated and intent-to-treat population: baseline detectable gVL, age, visit month, and ART group (combined only). Covariate set #1: Baseline detectable gVL, any RTI, age, visit month, and ART group (combined only). Covariate set #5: Baseline detectable gVL, any RTI, age, visit month, baseline pVL (dichotomous), pVL (log 10 continuous), and ART group (combined only). | | | |
