# Supplementary material for: Safety and continued use of the levonorgestrel intrauterine system as compared with the copper intrauterine device among women living with HIV in South Africa: A randomized controlled trial
Source: PLoS Med. 2020 May 22;17(5):e1003110. doi: 10.1371/journal.pmed.1003110 (PMC7244096; doi:10.1371/journal.pmed.1003110)
Supplement: S4 Table — ART, antiretroviral therapy; C-IUD, copper T-380 intrauterine device; LNG-IUS, levonorgestrel intrauterine system; pVL, plasma viral load (DOCX) [file pmed.1003110.s005.docx]

|  | **Detectable pVL by study visit**  **ART-using women (n= 132)** | **Change of log_10_ pVL at 6-month visit**  **Non-ART women (n= 67)** | |
| --- | --- | --- | --- |
|  | **Weighted OR (95% CI)** | **Weighted**  **difference (95% CI)** | |
| **As-treated analysis** |  |  | |
| Across 6 months (covariate set #1) | 0·82 (0·36–1·84) |  | |
| Across 6 months (covariate set #2) |  | -0·09 (-0·30–0·11) | |
| Across 24 months (covariate set #1) | 0·92 (0·47–1·78) |  | |
| Across 24 months (covariate set #2) |  | 0·03 (-0·37–0·43) | |
| **Intent-to-treat analysis** |  |  | |
| Across 6 months (covariate set #1) | 0·83 (0·37–1·87) |  | |
| Across 6 months (covariate set #2) |  | -0·07 (-0·26–0·12) | |
| Across 24 months (covariate set #1) | 0·87 (0·45–1·69) |  | |
| Across 24 months (covariate set #2) |  | 0·12 (-0·26–0·50) | |
| **Adjusted as-treated analysis** |  |  | |
| Across 6 months (covariate set #3) | 0·83 (0·37–1·87) |  | |
| Across 6 months (covariate set #4) |  | -0·11 (-0·31–0·09) | |
| Across 24 months (covariate set #3) | 0·92 (0·47–1·79) |  | |
| Across 24 months (covariate set #4) |  | 0·02 (-0·37–0·42) | |
| ART=antiretroviral therapy; CI=confidence interval; n=number; OR=odds ratio; pVL=plasma viral load; RTI=reproductive tract infection.  Covariate set #1: Baseline detectable pVL, and age. Covariate set #2: Baseline continuous pVL (log 10 continuous) and age. Covariate set #3: Baseline detectable pVL, any RTI, and age. Covariate set #4: Baseline continuous pVL (log 10 continuous), any RTI, and age. | | |  |

**S4 Table. Odds of detectable plasma viral load for women using ART or difference in mean change of log_10_ pVL among women not using ART at enrolment, comparing women using the levonorgestrel intrauterine system (LNG-IUS) with those using the copper T-380 intrauterine device (C-IUD), with linear regression weighted for differential intrauterine contraceptive discontinuation rates, among women living with HIV in Cape Town, South Africa**
