# Supplementary material for: Safety and continued use of the levonorgestrel intrauterine system as compared with the copper intrauterine device among women living with HIV in South Africa: A randomized controlled trial
Source: PLoS Med. 2020 May 22;17(5):e1003110. doi: 10.1371/journal.pmed.1003110 (PMC7244096; doi:10.1371/journal.pmed.1003110)
Supplement: S5 Table — ART, antiretroviral therapy; C-IUD, copper T-380 intrauterine device; LNG-IUS, levonorgestrel intrauterine system; pVL, plasma viral load (DOCX) [file pmed.1003110.s006.docx]

**S5 Table. Odds of detectable plasma viral load for women using ART or difference in mean change of log_10_ pVL among women not using ART at enrolment, comparing women using the levonorgestrel intrauterine system (LNG-IUS) with those using the copper T-380 intrauterine device (C-IUD), with linear regression adjusted by visit month, among women living with HIV in Cape Town, South Africa**

|  | **Detectable pVL by study visit ART-using women (n= 132)** | **Change of log_10_ pVL across 6- or 24-month visit  Non-ART women (n= 67)** |
| --- | --- | --- |
|  | **OR (95% CI)** | **Difference (95% CI)** |
| **As-treated analysis** |  |  |
| Across 6 months (covariate set #1) | 0·80 (0·35–1·80) |  |
| Across 6 months (covariate set #2) |  | -0·09 (-0·29–0·11) |
| Across 24 months (covariate set #1) | 0·90 (0·46–1·74) |  |
| Across 24 months (covariate set #2) |  | -0·02 (-0·38–0·34) |
| **Intent-to-treat analysis** |  |  |
| Across 6 months (covariate set #1) | 0·81 (0·36–1·83) |  |
| Across 6 months (covariate set #2) |  | -0·07 (-0·26–0·11) |
| Across 24 months (covariate set #1) | 0·90 (0·46–1·73) |  |
| Across 24 months (covariate set #2) |  | 0·03 (-0·31–0·36) |
| **Adjusted As-treated analysis** |  |  |
| Across 6 months (covariate set #3) | 0·80 (0·35–1·82) |  |
| Across 6 months (covariate set #4) |  | -0·11 (-0·30–0·09) |
| Across 24 months (covariate set #3) | 0·91 (0·47–1·76) |  |
| Across 24 months (covariate set #4) |  | -0·03 (-0·38–0·33) |
| ART=antiretroviral therapy; CI=confidence interval; n=number; OR=odds ratio; pVL=plasma viral load. RTI=reproductive tract infection. Covariate set #1: Baseline detectable pVL, age, and visit month. Covariate set #2: Baseline continuous pVL (log 10 continuous), age, and visit month. Covariate set #3: Baseline detectable pVL, any RTI, age, and visit month. Covariate set #4: Baseline continuous pVL (log 10 continuous), any RTI, age, and visit month. | | |
