# Supplementary material for: Safety and continued use of the levonorgestrel intrauterine system as compared with the copper intrauterine device among women living with HIV in South Africa: A randomized controlled trial
Source: PLoS Med. 2020 May 22;17(5):e1003110. doi: 10.1371/journal.pmed.1003110 (PMC7244096; doi:10.1371/journal.pmed.1003110)
Supplement: S6 Table — ART, antiretroviral therapy; C-IUD, copper T-380 intrauterine device; LNG-IUS, levonorgestrel intrauterine system; pVL, plasma viral load (DOCX) [file pmed.1003110.s007.docx]

**S6 Table. Odds of detectable plasma viral load for women using ART or difference in mean change of log_10_ pVL among women not using ART at enrolment, comparing women using the levonorgestrel intrauterine system (LNG-IUS) with those using the copper T-380 intrauterine device (C-IUD), with linear regression using working independent correlation structure, among women living with HIV in Cape Town, South Africa**

|  | **Detectable pVL by study visit ART-using women (n= 132)** | **Change of log_10_ pVL at 6- or 24-month visit Pre-ART women (n= 67)** |
| --- | --- | --- |
|  | **OR (95% CI)** | **Difference (95% CI)** |
| **As-treated analysis** |  |  |
| Across 6 months (covariate set #1) | 0·82 (0·36–1·83) |  |
| Across 6 months (covariate set #2) |  | -0·08 (-0·28–0·12) |
| Across 24 months (covariate set #1) | 0·97 (0·50–1·90) |  |
| Across 24 months (covariate set #2) |  | -0·02 (-0·38–0·34) |
| **Intent-to-treat analysis** |  |  |
| Across 6 months (covariate set #1) | 0·83 (0·37–1·87) |  |
| Across 6 months (covariate set #2) |  | -0·07 (-0·25–0·12) |
| Across 24 months (covariate set #1) | 0·88 (0·45–1·71) |  |
| Across 24 months (covariate set #2) |  | 0·04 (-0·30–0·38) |
| **Adjusted as-treated analysis** |  |  |
| Across 6 months (covariate set #3) | 0·81 (0·36–1·83) |  |
| Across 6 months (covariate set #4) |  | -0·10 (-0·29–0·09) |
| Across 24 months (covariate set #3) | 0·97 (0·50–1·91) |  |
| Across 24 months (covariate set #4) |  | -0·02 (-0·38–0·34) |
| ART=antiretroviral therapy; CI=confidence interval; n=number; OR=odds ratio; pVL=plasma viral load; RTI=reproductive tract infection.  Covariate set #1: Baseline detectable pVL and age. Covariate set #2: Baseline continuous pVL (log 10 continuous) and age. Covariate set #3: Baseline detectable pVL, any RTI, and age. Covariate set #4: Baseline continuous pVL (log 10 continuous), any RTI, and age. | | |
