# Supplementary figures and images for: Safety and continued use of the levonorgestrel intrauterine system as compared with the copper intrauterine device among women living with HIV in South Africa: A randomized controlled trial
Source: PLoS Med. 2020 May 22;17(5):e1003110. doi: 10.1371/journal.pmed.1003110 (PMC7244096; doi:10.1371/journal.pmed.1003110)

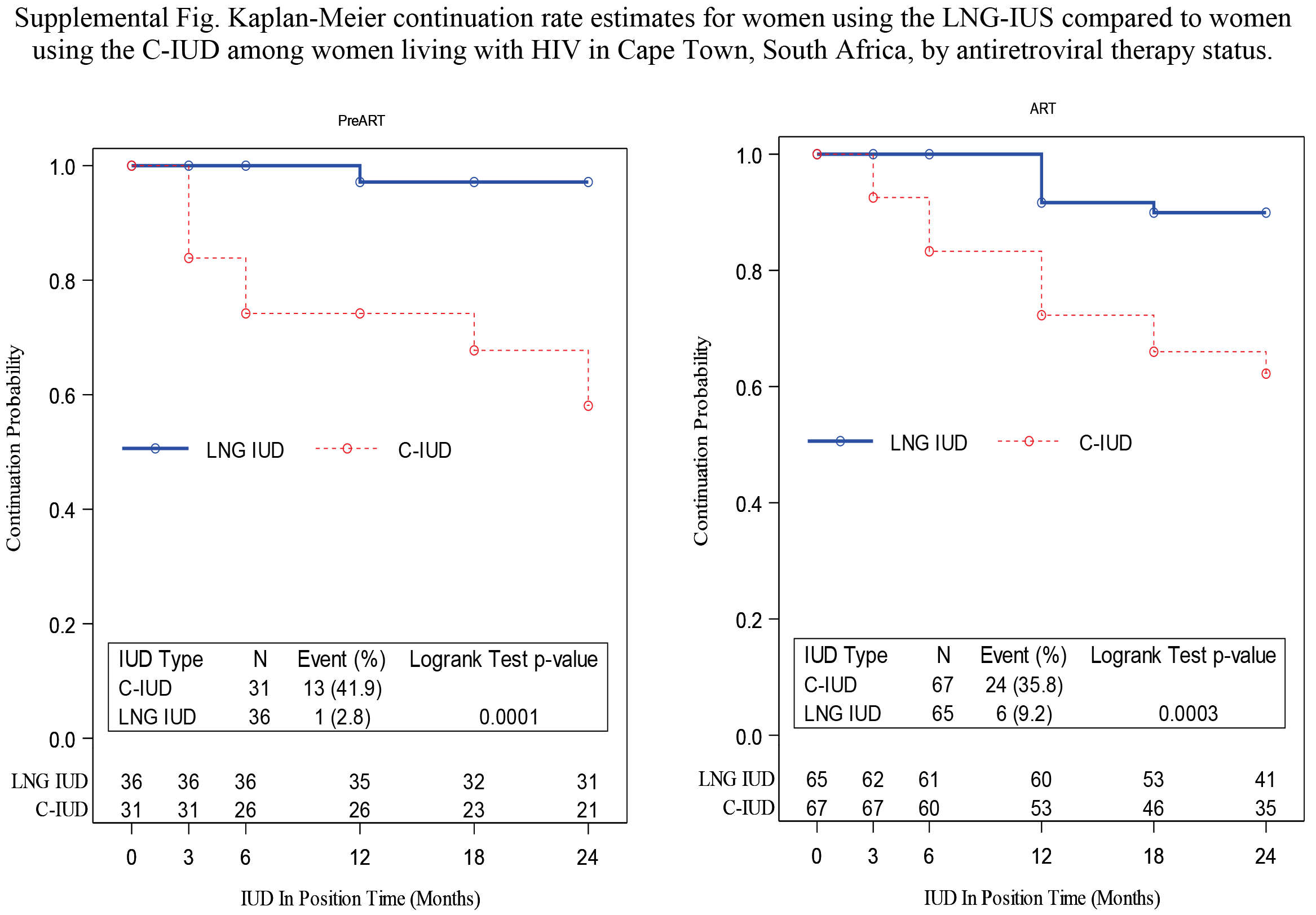

Supplement: S1 Fig — C-IUD, copper T-380 intrauterine device; LNG-IUS, levonorgestrel intrauterine system (TIF) [file pmed.1003110.s008.tif]
