# Supplementary material for: Safety and continued use of the levonorgestrel intrauterine system as compared with the copper intrauterine device among women living with HIV in South Africa: A randomized controlled trial
Source: PLoS Med. 2020 May 22;17(5):e1003110. doi: 10.1371/journal.pmed.1003110 (PMC7244096; doi:10.1371/journal.pmed.1003110)
Supplement: S1 Text — FHI 360 Study 10369, Version 10.0. (PDF) [file pmed.1003110.s009.pdf]

Comparison of Two IUDs among Cape Town HIV-positive Women: A Randomized Controlled Trial Assessing Safety of Registered Products in South Africa

FHI 360 Study 10369

Version 10.0

**Sponsor:**	FHI 360<br>P. O. Box 21059<br>Durham, NC 27703, USA
**Funded by:**	Eunice Kennedy Shriver National Institute of Child Health & Human Development of the National Institutes of Health; Preventive Technologies Agreement, United States Agency for International Development
**Principal Investigator (Site):**	Benjamin Landon Myer, PhD, MBChB<br>Associate Professor<br>School of Public Health & Family Medicine,<br>University of Cape Town<br>Anzio Road, Observatory 7925<br>Cape Town, South Africa<br>+27 21 406 6661
**Medical Monitor/<br>FHI360 Project Leader:**	Catherine Todd, MD, MPH<br>Scientist I, Clinical Sciences<br>FHI 360<br>P.O. Box 21059<br>Durham, NC 27703, USA<br>+1-317-372-3566
**Clinical Site:**	Gugulethu Community Health Centre (GCHC)<br>Cnr Ny1 & Ny 3, 7750<br>Gugulethu, Western Cape, South Africa
**Study Laboratory:**	National Health Laboratory Services<br>C18, Groote Schuur Hospital<br>Observatory 7925, Cape Town, South Africa<br><br>UCT Medical Microbiology Laboratory<br>Institute of Infectious Disease and Molecular Medicine,<br>University of Cape Town,<br>Anzio Road, Observatory 7925,<br>Cape Town, South Africa

Comparison of Two IUDs among Cape Town HIV-positive Women: A Randomized Controlled Trial Assessing Safety of Registered Products in South Africa

FHI 360 Study 10369/ IRBNet# 398733

I, the Principal Investigator/Medical Monitor, agree to conduct this study in full accordance with the provisions of this protocol, the regulatory requirements of South Africa, and in accordance with the principles of Good Clinical Practice described in the International Conference of Harmonization E6 (R1), the Declaration of Helsinki and the Statement of Investigator, which I have also signed. The trial will be conducted in accordance with the relevant laws and regulations relating to clinical research and the protection of subjects in the country in which the trial will be performed. I agree to maintain all study documentation until FHI 360 advises that it is no longer necessary.

I have read and understand the information in this protocol, including the potential risks and side effects of the study products, and will ensure that all associates, colleagues, and employees assisting in the conduct of the study are informed about the obligations incurred by their contribution to the study.

____________________________________ ____________________________________

Signature of the Site Principal Investigator Date
(Benjamin Landon Myer)

____________________________________ ____________________________________

Signature of Medical Monitor Date
(Catherine Todd)

TABLE OF CONTENTS

1.0 PROTOCOL TEAM ROSTER 7
2.0 LIST OF ABBREVIATIONS AND ACRONYMS 8
3.0 STUDY SUMMARY 10
4.0 INTRODUCTION 12
4.1 Rationale for trial 13
5.0 STUDY OBJECTIVES AND ENDPOINTS 14
5.1 Primary Objective 14
5.2 Secondary Objectives 14
5.3 Primary Endpoints 15
5.4 Secondary Endpoints 15
6.0 OVERVIEW OF STUDY DESIGN 15
6.1 Summary of Amendment 1 (V3.0/3.1) Changes: 15
6.2 Summary of Amendment 2 (V4.0) Changes: 16
6.3 Summary of Amendment 3 (V5.0) Changes: 16
6.4 Summary of Amendment 4 (V6.0) Changes 17
6.5 Summary of Amendment 5 (V 7.0) Changes 18
6.6 Summary of Amendment 6 (V 8.0) and Amendment 7 (v9.0) Changes 19
7.0 STUDY POPULATION 20
7.1 Eligibility Criteria and Rationale 20
7.2 Recruitment 23
7.3 Retention 24
8.0 STUDY PRODUCTS 24
8.1 Packaging and Labeling of Study Product 25
8.2 Product Storage and Temperature 25
8.3 Accountability 25
9.0 STUDY PROCEDURES 25
9.1 Screening visit 25
9.2 Enrollment and insertion visit 27
9.3 Follow-up visits 28
9.4 Closing visit (24 months) 29

9.5 Product and Study Discontinuation and Incident Pregnancy 30
10.0 SAFETY MONITORING AND ADVERSE EVENT (AE) REPORTING 32
10.1 AE Definitions 32
10.2 AE Grading 33
10.3 AE Relationship to Study Product 33
10.4 Medical Management of AEs 33
10.5 Serious Adverse Events 34
10.6 SAE Reporting 35
10.7 Data, Safety, Monitoring Board 35
10.8 Social Harm Events 35
11.0 SAMPLE SIZE JUSTIFICATION & APPROACH TO ANALYSIS 35
11.1 Sample Size Justification 36
11.2 Primary Outcome Analysis 37
11.3 Secondary Outcomes Analysis 38
11.4 Interim analysis 40
11.5 Qualitative analysis 40
12.0 RANDOMIZATION AND BLINDING 41
12.1 Randomization 41
12.2 Blinding 41
13.0 DATA MANAGEMENT PLAN SUMMARY 41
13.1 Study Instruments 42
14.0 HUMAN SUBJECTS CONSIDERATIONS 43
14.1 Institutional Review Board Review and Approval 43
14.2 Informed Consent 43
14.3 Risks 44
14.4 Benefits 45
14.5 Confidentiality 45
14.6 Alternatives 46
14.7 Community Involvement and Consultation 46
14.8 Research Integrity 46
14.9 Study Discontinuation 47

15.0 CLINICAL MONITORING SUMMARY 47
16.0 STEERING COMMITTEE 47
17.0 ADMINSTRATIVE 48
17.1 Protocol Violations 48
17.2 Study Records 48
17.3 Publication Policy 48
17.4 Research at External Sites 48
18.0 TIMELINE 49
19.0 REFERENCES 50
20.0 APPENDICES 58
Appendix 1: Schedule of Activities by Study Visit 58
Appendix 2: IUD Insertion Risk Screening Instrument 59
Appendix 3: Lab Considerations 61

1.0 PROTOCOL TEAM ROSTER

Benjamin Landon Myer, PhD, MBChB
Principal Investigator (Site)
School of Public Health & Family Medicine, University of Cape Town
Anzio Road
Observatory 7925
Cape Town, South Africa

Catherine Todd, MD, MPH
Medical Monitor/ FHI 360 Project Leader
FHI 360
359 Blackwell Street, Suite 200
Durham, NC 27701 USA

Linda-Gail Bekker, PhD, MBChB,
Co-Investigator
University of Cape Town, South Africa
2 Rhodes Avenue
Mobray, South Africa

Heidi Jones, PhD, MPH
Statistician,
Hunter College, New York
2180 Third Avenue
New York, NY 10035, USA

Donald Hoover, PhD
Biostatistics Consultant
Indicium Data Insight, LLC
150 Brittany Way
Bluebell, PA 19422, USA

2.0 LIST OF ABBREVIATIONS AND ACRONYMS

AE	adverse event
AIDS	Acquired Immune Deficiency Syndrome
ANCOVA	analysis of covariance
ART	antiretroviral therapy
ARV	antiretroviral
ASCUS	atypical squamous cells of undetermined significance
BV	bacterial vaginosis
CAB	community advisory board
CD4	cluster of differentiation 4
C-IUD	copper intrauterine device
COCs	combined oral contraceptives
CT	*Chlamydia trachomatis*
CUNY	The City University of New York
DAIDS	Division of Acquired Immunodeficiency Syndrome
DTHC	Desmond Tutu HIV Centre
DMPA	depot medroxyprogesterone acetate
DSMB	Data and Safety Monitoring Board
ECS	endocervical sampling
FWA	federal wide assurance
GCHC	Gugulethu Community Health Centre
GEE	generalized estimating equations
hCG	human chorionic gonadotropin
Hgb	hemoglobin
HIV	Human Immunodeficiency Virus
IC	informed consent
IDI	in-depth interview
IUD	intrauterine device
IRB	institutional review board
LARC	long-acting reversible contraceptive method
LNG IUD	levonorgestrel intrauterine device
LSIL	low-grade squamous intraepithelial lesion
MCC	Medicines Control Council
NetEn	norethisterone enanthate
NG	*Neisseria gonorrhoeae*
OB/Gyn	obstetrics and gynecology
ORTHO BC-SAT	birth control satisfaction assessment tool
PCR	polymerase chain reaction
PH	proportional hazards
PHSC	Protection of Human Subjects Committee

PI	Principal Investigator
PID	pelvic inflammatory disease
PMTCT	prevention of mother-to-child transmission
RCT	randomized controlled trial
RNA	ribonucleic acid
RPR	rapid plasma reagin
SAE	serious adverse event
SF-12	12-Item short form survey
STI	sexually transmitted infection
TP	*Treponema pallidum*
TV	*Trichomonas vaginalis*
UCT	University of Cape Town
μL	microliter
VL	viral load
WHO	World Health Organization

3.0 STUDY SUMMARY

Comparison of Two IUDs among Cape Town HIV-positive Women:
A Randomized Controlled Trial Assessing Safety of Registered Products in South Africa

Design: Single site, double-blind, randomized controlled trial

Population: HIV-positive South African women between the ages of 18 and 40 years

Study size: At least 166 women; at least 66 ineligible for antiretroviral therapy (pre-ART) and at least 100 women using ART at entry.

Study intervention: Levonorgestrel intrauterine device (LNG IUD) or the copper T-380 intrauterine device (C-IUD), which are registered devices in South Africa for this patient population.

Duration & Follow-up: Approximately 48 months in total. Recruitment will take approximately 38 months. After enrollment, each participant will be followed for up to 24 months, as allowed within the 48 month clinical period.

Primary Objectives: To compare LNG IUD safety to the safety of the C-IUD with respect to genital HIV shedding, a surrogate for potential for HIV transmission, overall and in the presence and absence of ART.

Secondary Objectives: 1) To compare LNG IUD safety to the safety of the C-IUD with respect to HIV progression as measured by changes in plasma viral load (VL) at 6 months between the two pre-ART study arms.

2) To explore LNG IUD safety with respect to non-HIV related outcomes, including hemoglobin change, incidence of sexually-transmitted infections (STIs) and pelvic inflammatory disease (PID).

3) To measure LNG IUD acceptability through device continuation and other measures for the LNG IUD as compared to the C-IUD.

4) To compare three genital tract sampling methods to determine which provides optimal recovery of HIV RNA and immune mediators over a series of three visits.

Primary Endpoints: Change in detection and quantity of HIV RNA genital VL measures between study arms at baseline and 6 months, and over 24 months following IUD insertion.

Secondary Endpoints: 1) Measures of HIV disease progression, including CD4 change, time to ART initiation, and mean plasma VL change from baseline to 6 and through 24 months among pre-ART women.

2) Hemoglobin change and incidence of STIs and PID for all participants through 24 months.

3) IUD continuation and expulsion rates between study arms comparing different IUDs over a 24-month period and acceptability measures through quantitative and qualitative methods.

4) Comparison of female genital tract sampling methods for recovery of HIV RNA as measured by VL and immune mediators (e.g. cytokines).

Study Site: Gugulethu Community Health Centre (GCHC), Cape Town, South Africa

4.0 INTRODUCTION

Family planning and preventing mother to child transmission (PMTCT) of Human Immunodeficiency Virus (HIV) through effective contraception are increasingly important. Analyses assessing various approaches to reducing mother to child HIV transmission conclude that preventing unplanned pregnancy is the most cost-effective PMTCT approach,[1-3] spurring recommendations to integrate HIV care and reproductive health services.[4] However, some studies have called into question the safety of systemic hormonal contraceptives for HIV-positive women and their partners.[5-8]

With the global roll-out of antiretroviral therapy (ART) and expanded eligibility criteria for ART use, population coverage of ART use is continuing to increase rapidly in South Africa and other settings where HIV is prevalent. In 2013, the World Health Organization (WHO) recommended initiation of ART in adults based on CD4≤500 cells/mm^3 and/or for all HIV-infected pregnant women regardless of CD4 cell count.[9] These guidelines were recently adopted by the South African Department of Health, and, by one modeling estimate, will see approximately 60% of HIV-infected women of reproductive age in Cape Town on ART by 2016 and 80% by 2020 (personal communication, L. Johnson).

Data indicate that viral shedding occurs among some women with suppressed plasma viral load (VL) on various ART regimens, with ranges of 5 to 45% of women having detectable genital tract VL in specimens from at least one measurement.[10-14] To that end, there are few available data regarding impact of exogenous progestins on genital tract shedding. Depot medroxyprogesterone acetate (DMPA) does not appear to increase genital tract shedding for women using ART in Kenya.[11] Two longitudinal cohort studies with the LNG-IUD did not detect increased viral shedding following IUD insertion; however, one study involved only ART-naïve women and the other had a mix of women using and not using ART and both had small sample sizes.[15,16] However, genital tract HIV RNA increases risk of HIV transmission to male partners and hormonal contraception appears to increase these odds based on data from a large observational analysis of serodiscordant couples.[8] With the expansion of ART coverage, the safety of hormonal contraceptive methods with regard to transmission to male partners is of increasing importance as a measure of contraceptive method safety.

HIV progression among pre-ART women is also an important issue as ART initiation has important logistical and cost challenges. ART-naïve HIV-positive women in Zambia using DMPA or combined oral contraceptives (COCs) progressed more quickly to death or CD4<200 cells/mm^3 than women using the copper intrauterine device (C-IUD).[6,7] Of note, though this is the only randomized controlled trial (RCT) published to date, the cohort had high method discontinuation and switching rates, that could potentially bias the results.[6,7] More broadly, data regarding impact of progestin-containing methods on HIV progression are mixed.[17-25] The posited mechanism is increased HIV-1 plasma VL with progestin exposure, based on human and macaque data.[18-20] However, analyses of HIV-positive cohorts have not found any association between reported systemic hormonal method use and progression to death or ART initiation.[21-25] While these data are reassuring, three articles and a recent WHO Technical Statement and Policy Implication publications conclude that more high-quality evidence concerning the safety of hormonal contraceptive use for HIV-positive women is needed to inform medical guidelines.[1,26-29]

Long-acting reversible contraceptive (LARC) methods potentially play an important role in preventing unplanned pregnancy for women in resource-challenged settings and efforts are underway to expand the availability of high quality methods, some of which contain progestin, at a sustainable cost. Among these LARC methods is the LNG IUD. Currently, the only available LNG IUD is manufactured by Bayer Health Care. However, a generic LNG-20 device, bioidentical to Mirena IUD, is in development and is poised to make the LNG-IUD affordable in resource-limited settings.[30,31] The LNG IUD is an excellent candidate method for HIV-positive women, as it has >1 log lower circulating progestin levels compared to systemic hormonal methods (COCs and DMPA), potentially making it a safer choice than DMPA.[32-34] The LNG IUD is likely to be safer than systemic hormonal methods like DMPA and COCs as the relatively higher levels of circulating progestin have been implicated in faster HIV progression and transmission to male partners, with potential indication of a dose-response relationship as DMPA is more consistently associated with transmission and progression outcomes than are COCs.[6-8] According to WHO Medical Eligibility Criteria, DMPA, COCs, and the LNG IUD are deemed safe for use by HIV-positive women (Classes 1,1, and 2, respectively).[35] However, DMPA and COC safety for HIV-positive women has been called into question; [5-8, 29] thus, any method utilizing progestin as part of its mechanism of action needs definitive evidence to ensure it may be safely used by HIV-positive women. LNG IUD safety data for HIV-positive women are limited to one case-control study and two small series, which indicate the device did not increase progression rates by CD4 count. [16,36-38] However, these studies involved small samples of women without disaggregation by ART use, and thus cannot definitively establish LNG IUD safety. Thus, the safety of the LNG IUD with respect to HIV progression and transmission is not clear.

The LNG IUD is an excellent method for many women and confers additional advantages to reliable contraception to HIV-positive women by reducing menstrual blood loss and medical visits compared to other hormonal methods.[38-40] While the C-IUD is available in the public sector of South Africa and many sub-Saharan African countries and considered safe with respect to HIV progression,[35,41] it is not widely offered or used.[42] A study of 205 women in two South African provinces found that, while 26% of women were aware of the C-IUD, 74% were interested in IUD use after method counseling.[43] This data, as well as a recent study assessing perceptions regarding LARC methods, including the IUD, among postpartum HIV-positive and negative women in Cape Town, suggests that the IUD is not a method for which many women receive counseling and many providers eschew due to potentially erroneous concerns about PID.[44,45] Due to acceptability of contraceptive-induced oligo-amenorrhea in South Africa, we hypothesize that the LNG IUD will be more acceptable than the C-IUD. In our preliminary work among 277 HIV-positive women in Cape Town, of whom 35% were not ART-eligible, 37% were aware of and 2% had ever used IUDs. However, upon receiving information about the IUD, 86% of women who had not undergone surgical sterilization previously were willing to consider the IUD for contraception.[46] Though these feasibility data are encouraging, measures of acceptability, with IUD continuation being the most robust measure, are needed to determine the practicality of IUD scale-up and counseling considerations for HIV-positive women.

4.1 Rationale for trial

The rationale for this study is the need for high-quality clinical evidence to determine whether the LNG IUD is safe for HIV-positive women with regard to HIV potential transmission to male partners and disease

progression.[29] The lack of evidence to suggest that adverse progestin-mediated effects might be associated with the LNG IUD, coupled with impending LNG IUD affordability, indicate that now is the time to establish LNG IUD safety and acceptability for HIV-positive women. Thus, this RCT is needed to document safety and acceptability of the candidate method (LNG IUD) compared to a non-hormonal method with documented safety with regard to HIV transmission and progression (C-IUD).

5.0 STUDY OBJECTIVES AND ENDPOINTS

This study will compare the LNG IUD to the non-hormonal C-IUD in a RCT comparing genital tract and plasma HIV RNA VL and method continuation among treatment arms each with at least 66 pre-ART HIV-positive women and at least 100 HIV-positive women taking ART over two years ($n \geq 166$, to a maximum of 288 total). The IUD devices for this study are registered in South Africa for use among this patient population.

5.1 Primary Objective

To compare LNG IUD safety to the safety of the C-IUD with respect to genital HIV RNA shedding, a surrogate for potential for HIV infection, overall and in the presence or absence of ART.

Hypothesis 1: The LNG IUD will not increase genital tract HIV shedding, as measured by genital tract VL and rates of genital tract HIV RNA shedding through 6 months, compared to the C-IUD among HIV-positive women in Cape Town, South Africa.

5.2 Secondary Objectives

To compare LNG IUD safety to the safety of C-IUD with respect to HIV progression as measured by changes in plasma viral load (VL) at 6 months between the two pre-ART study arms

Hypothesis 2: The LNG IUD will not increase HIV progression, as compared to the C-IUD at 6 months (measured by plasma VL), among pre-ART HIV-positive women in Cape Town, South Africa.

To measure LNG IUD acceptability through device continuation and other measures for the LNG IUD as compared to the C-IUD.

Hypothesis 3: The LNG IUD will be more acceptable, as measured by device continuation rates through 24 months, than the C-IUD among HIV-positive women in Cape Town, South Africa.

To compare three genital tract sampling methods to determine which provides optimal recovery of HIV RNA and immune mediators over a series of three visits.

Hypothesis 4: Menstrual cup (MC) specimens will provide greater recovery of genital tract HIV RNA and immune mediators than the other two methods.

To explore LNG IUD safety with respect to non-HIV related outcomes, including hemoglobin change, incidence of sexually-transmitted infections (STIs) and pelvic inflammatory disease (PID).

5.3 Primary Endpoints

- Change in detection and quantity of HIV RNA genital VL measures between study arms at baseline and 6 months and over 24 months following IUD insertion.

5.4 Secondary Endpoints

- Measures of HIV disease progression, including CD4 change, time to ART initiation, and mean plasma VL change from baseline to 6 and through 24 months among pre-ART women.
- Hemoglobin change and incidence of STIs and PID for all participants through 24 months.
- IUD continuation and expulsion rates between study arms comparing different IUDs over a 24-month period and acceptability measures through quantitative and qualitative methods.
- Comparison of female genital tract sampling methods for recovery of HIV RNA as measured by VL and immune mediators (e.g. cytokines).

6.0 OVERVIEW OF STUDY DESIGN

This study is a single site, double-blind, parallel RCT in Gugulethu, Cape Town, South Africa with at least 166 HIV-positive women with inclusion of women not yet eligible for ART and those using ART. Both pre-ART and ART-using participants will be randomized in a 1:1 ratio to (LNG IUD) and (C-IUD) arms and followed at 3, 6, 12, 18, and 24 months, as possible, to determine comparative safety in terms of HIV transmission, progression, and acceptability of the LNG IUD. Both groups will receive individual risk-reduction counseling, sexually transmitted infection (STI) screening, treatment for any diagnosed curable STI, and condoms free of charge. The anticipated total study duration is approximately 60 months, with recruitment taking approximately 38 months and participant follow-up of up to 24 months. A 24 month follow-up period was selected because the IUD is meant to be a LARC method and follow-up for a minimum of 24 months permits determination of whether the device is feasible as a LARC method for this population.[35]

6.1 Summary of Amendment 1 (V3.0/3.1) Changes:

Additional funding has been identified to compare three genital tract sampling methods with regard to efficacy for measurement of HIV RNA and immune mediators. The three methods to be compared are endocervical sampling with a flocked swab alone (ECS), swab-enriched lavage using the same ECS method followed by a 5 mL lavage of the genital tract, and menstrual cup (MC) collection over varying time periods before examination. All participants will be asked to insert a disposable MC prior to the enrollment/ insertion, 3 month, and 6 month examinations for collection of genital tract secretions. Women will also be asked to consent to having an extra ECS specimen and a lavage with 5 mL taken in addition to the originally-planned ECS specimens and STI testing. Additional *Neisseria gonorrhea* (NG) and *Chlamydia trachomatis* (CT) testing will be performed at the 3 and 6 month visits to assess the impact of concomitant genital tract infection on HIV RNA quantity and recovery between sampling methods, as permitted by funding.

Due to funding reductions by NICHD, the 9 month follow-up visit has been removed and the sample size has been reduced to 144 women/ arm, for a total of 288 participants. The sample size justification has been modified and the arm size of 144 women provides sufficient power to detect a 0.5 $\log_{10}$ difference in mean plasma VL change over time. Please see the modified sample size rationale in Section 11.1 for further details.

6.2 Summary of Amendment 2 (V4.0) Changes:

The interim analysis and outcome assessment of the first primary aim will be performed at completion of the 6- month VL measures. The reason for changing the time period for primary outcome analysis is two-fold: VL changes more rapidly than does CD4 lymphocyte count and is a more sensitive measure of HIV disease progression; thus, changes should become apparent by 6 months following IUD insertion. Second, WHO criteria for ART initiation have recently changed to recommend initiation at CD4 count of 500 cells/mm^3, where possible.[9] At this time, there are no plans to change the South African ART guidelines from an initiation level of 350 cells/mm^3, but limiting the outcome period to 6 months at analysis will ensure that, should the national guidelines change, women will be able to expeditiously enter into ART while still completing at least the first 6 months of study follow-up before being censored for the primary safety outcome.

The informed consent and questionnaire forms have been pilot-tested with resultant minor changes, primarily to make the questionnaire instruments more concise. Afrikaans has been removed as a language for study use as isiXhosa is the predominant language of the study population. The interval questionnaire has been revised and made specific to each follow-up visit for greater efficiency and questionnaire supplements for exit and IUD discontinuation visits have been added.

The initial CD4 count has been changed to the screening visit in lieu of the enrollment visit to ensure greater accuracy in screening.

The selection and entry of focus group discussion participants has been provided in greater detail.

The laboratory plan for testing has been articulated in greater detail, with change in assay for NG/CT testing.

Study clinical monitoring will be performed by Westat Corporation.

Unscheduled visits will be reimbursed to the value of 150 Rand (approximately US$18.75).

Prior ectopic pregnancy has been added as an exclusion criterion, at request of the South African Medicines Control Council (MCC).

Change in cervical cytology inclusion criterion to allow women with no record of pap smear and with no gross evidence of cervical neoplasia on screening examination to enter the study with cytologic assessment performed at screening examination.

6.3 Summary of Amendment 3 (V5.0) Changes:

With the change of performing primary outcome analysis at six months, the inclusion criterion for CD4 count for study entry is changed to >350 cells/mm^3; 350 cells/mm^3 continues to be the threshold for ART initiation in South Africa. Limiting the primary analysis to six months will ensure sufficient data collection for the plasma VL outcome, while maximizing eligible participants without compromising timing of ART initiation.

Eligibility is limited to women not desiring pregnancy for the next two years. This has been clarified.

The method for calculating sample size has been modified from t-test to ANCOVA, with the current sample size providing sufficient power to detect at least a 0.32 log_{10} difference in mean plasma VL change from baseline to six months.

The anticipated participant recruitment period has been increased to 18 months, based on observed recruitment rates during study start.

Women consenting to screening and who are ineligible or decline entry after screening is initiated will be requested to complete the ineligible/decliner questionnaire in the screening ICF with their screening ID number as the sole identifier. This change has been made to the screening ICF.

Contact information in the IC forms has been updated to include the MCC.

Questionnaires have been formatted and some questions/response options clarified with some questions transferred to clinical forms; clinical forms requesting data directly from participants have been submitted for approval.

6.4 Summary of Amendment 4 (V6.0) Changes

South Africa recently broadened lifelong ART eligibility to include all HIV-positive individuals with CD4<500 cells/mm^3, all women initiating ART during pregnancy, and all diagnosed with tuberculosis co-infection to start sometime in 2015. In light of this change and slow recruitment, two additional arms of ART-using women with documented viral suppression (<1000 copies/mL) have been added, who will be randomized 1:1 to the C-IUD and LNG IUD. Even with more women taking ART, genital tract shedding still occurs and potentially poses a risk for HIV acquisition to male partners, as reviewed in the literature cited in the Introduction (Section 4.0). The primary objective has thus been modified to compare genital tract HIV shedding, a proxy measure for potential for HIV transmission to uninfected male partners. This outcome is relevant to both women using ART and those who are not yet eligible for ART. Related modifications have also been made to the eligibility criteria, laboratory testing, sample size, analysis, and interim analysis sections. There is no change to planned biologic sampling at each visit and plasma and genital tract VLs will be measured in real time.

Due to minimal contribution to the primary or secondary outcome measures, funding constraints, and slow recruitment precluding group formation within a six month period, focus group discussions have been removed from the protocol.

Due to lower than anticipated eligibility among women screened for the study, screening estimates have been adjusted from 420 to 1000 women.

Menstrual diaries were originally included to provide another means of recording bleeding pattern not subject to recall bias, as an additional measure of acceptability. However, due to poor completion rates to date and difficulty reaching patients for one-month follow-up calls with limited staffing resources,

menstrual diaries have been removed as a data collection tool with accompanying edits to the analysis section.

The time periods during which screening visit STI test results are valid for enrollment have been updated, as the mismatch of 21 days from a negative test and 14 days following treatment for a positive test are causing confusion with no evidence to mandate these time periods. We have thus elected to require 28 days or less from screening test results and the need for re-consenting women who present outside their window for enrollment, which are now clarified in the protocol and Screening ICF.

Clarification that HIV status may be determined by rapid test, with on-site counseling and testing, for women with no other documentation of infection during screening.

Clarification regarding management of IUD expulsion has been added.

Timing of tuberculosis diagnosis has been clarified regarding the exclusion criterion for pre-ART participants.

Clarification that *N. gonorrhea* and *C. trachomatis* diagnosis and treatment at the time of follow-up visit will be by syndromic management, consistent with the national standard of care in SA.[55] Testing will be performed in real time as funding allows.

Clarification of conditions ranked as SAEs and definition of worsening of a pre-existing condition to be regarded as an AE.

6.5 Summary of Amendment 5 (V 7.0) Changes

The nested comparison between sampling techniques sampling and testing is complete. We have clarified the protocol that all further participants will only have menstrual cup samples taken for genital tract VL measures and one endocervical swab obtained for additional testing purposes at all visits.

Clarification that screening participants with reactive syphilis tests and measurable serologic titres may proceed with enrollment while receiving treatment for syphilis.

Clarification that all participants will have plasma HIV RNA VL measurement at the enrollment visit, regardless of ART status.

Clarification that women with ongoing AEs at the time of study exit/24 month visit will receive follow-up phone calls to assess status of AEs and arrange care as appropriate.

Clarification that genital tract specimen processing will occur in the Medical Microbiology laboratory at UCT.

Minor administrative and editorial changes were also incorporated.

6.6 Summary of Amendment 6 (V 8.0) and Amendment 7 (v9.0) Changes

Due to lower than expected enrollment figures, the recruitment period is extended through 2016 and the sample sizes and associated power calculations have been adjusted to accommodate potentially lower numbers, with a minimum of 166 participants (66 ART-ineligible and 100 ART-users) to a maximum of the previously-planned sample size of 288.

Updating of ART referral policy based on OCT 2016 change in Western Cape HIV care guidelines.

Addition of testing of stored samples for additional biomarkers (including but not limited to mucosal cytokine measures, HSV-2, and human papilloma virus (HPV) subtypes) for participants agreeing to supplemental testing of residual samples in the approved trial entry consent (this has always been present in the trial entry consent). Supplemental funding has been received from NICHD to test a portion of residual genital tract samples from ART-using women for mucosal cytokine markers and HPV and to test these and the residual specimens from the first 50 pre-ART participants for HSV-2. These tests will enhance primary outcome measure interpretation as well as provide insights to genital tract inflammatory differences in the presence of ART as well as between the two IUD types (after unblinding in final analysis).

Clarification of timeline and enrollment period length related to the above recruitment extension.

Clarification of follow up of adverse events that are still present at study exit.

6.7. Summary of Amendment 8 (V 10.0) Changes

Addition of study close unblinding visit: Due to budgetary necessity, the study will close activities at end-June 2018, at which time approximately 20 participants will not yet be eligible for their 24 month final visit. The amendment requests allowance of a final visit at which women are unblinded to IUD type and offered STI testing prior to completing the 24 month visit (those within the window for their 24 month visit will complete normal visit procedures up to June 1, 2018, after which time they will be provided the described modified final visit). These visits will be offered through June 21, 2018 with the days remaining in June for follow-up of STI results and any remaining AEs. Ongoing AEs noted at the time of unblinding visit will receive a referral form on that date and not have further follow-up through the study.

Addition of IUD continuation telephone calls to those women exiting prior to 24 months and electing to retain the IUD: For women who are not eligible for their 24 month visit at study clinical activity closure, those women that come for the previously-described final visit for unblinding and elect to retain their IUD will be asked if they would be willing to be called by telephone within the window of their 24 month visit to record whether they have continued using the IUD, their current level of method satisfaction, and, if they have had the IUD removed, when it was removed, the reasons for removal and the new method they are using, if any.

7.0 STUDY POPULATION

The study will include at least 66 HIV-positive South African women ineligible for ART at study entry based on CD4 count and lack of AIDS-defining illnesses and at least 100 women using ART with plasma VL<1000 copies/mL at the most recent VL measure. Please see Sections 10.4 and 11.2 for management of women transitioning to ART during the study period, accommodation of ART initiation, and approaches for pooled analysis with ART-using and pre-ART women in the analysis.

7.1 Eligibility Criteria and Rationale

To be eligible for inclusion in the study, the participant must meet all of the following criteria:

- Willing and able to provide written informed consent (IC) to be screened for and to participate in the trial
- Interested and willing to use the IUD as a family planning method.
- Between 18 to 40 years of age (inclusive): This age range includes women during their years of greatest fertility and 18 is the age of majority for research consent in South Africa.
- Willing to participate in all aspects of the study and to comply with study procedures and visits, for 24 months, including:
    - Be randomized
    - Adhere to follow-up schedule and willing to be contacted by site staff between study visits (by phone and/or in person)
    - Provide contact/locator information
    - Agree for site staff to review clinic chart to confirm HIV status
- Has documented HIV infection: We will be recruiting women from HIV care sites where patients seeking care at that site have documented HIV infection and know their HIV status. Patient and chart confirmation or, absent this, an on-site reactive HIV rapid test will be required for eligibility.
- For pre-ART entrants:
    - Not currently using ART and no history of ART use within the last six months. All HIV-infected individuals in Western Cape Province are eligible for ART initiation but ART implementation will be prioritized to individuals meeting prior criteria by CD4 count <500 cells/mm^3 or certain conditions, including pregnancy and tuberculosis. All women screened with documented HIV infection not currently using ART will be referred for ART initiation, at the discretion of their regular HIV care provider and in compliance with Western Cape Province guidelines.
    - Be at least 6 months post-delivery and not pregnant or desiring pregnancy for the next 30 months. Women receiving ARV prophylaxis for PMTCT in pregnancy who then elect to discontinue ART use will have enrollment deferred to at least 6 months post-ARV exposure before they may enter the pre-ART arm.[49,50] Though these parameters indicate the period by which resistance mutations subside, we expect this to be a very conservative measure

compared to the paucity of data on return to VL levels among women discontinuing ART within PMTCT regimens.

- For ART-using entrants:
    - ART-use demonstrated by clinical records reflecting laboratory measures consistent with ART use and evidence of viral suppression (plasma VL<1000 copies/mL) at VL measure within the last 12 months.
    - Be at least 6 weeks post-delivery and not pregnant or desiring pregnancy for the next 30 months.[1] Women with a recent spontaneous or elective first trimester abortion will be eligible for study entry 4 weeks following the event.

IUDs are intended to be long-acting reversible methods. For this reason, women currently pregnant or desiring pregnancy in the next 30 months will be excluded. Pregnancy tests will be performed at screening, insertion, and each follow-up visit to rule out pregnancy for all participants (see Appendix 1 for schedule of activities). For women using injectable contraception and experiencing secondary amenorrhea, pregnancy testing will be performed at screening and insertion will be scheduled within the same week that the next injection would be due.

- Intending residence in Cape Town area for next 30 months: Women with plans to move or whose employment causes seasonal migration will be excluded from the study.
- No documented or known history of infertility or sterilization: All participants should have no history of surgical sterilization or infertility to ensure that device satisfaction and discontinuation is informed from the perspective of avoiding unintended pregnancy.
- No gross evidence of cervical neoplasia on examination: HIV-positive women have increased susceptibility to cervical neoplasia and by South African care standards, receive cytologic screening at least every 36 months. For women with no cytology report, a Pap smear will be performed at screening examination unless there is evidence of a cervical lesion suspicious for neoplasia, in which event the woman will be referred for colposcopy and biopsy. A normal, ASCUS, or LSIL result reduces the chance that any abnormal bleeding is a symptom of advanced cervical neoplasia; all cytology results will be received and reviewed by study clinical staff with appropriate referral for abnormal results.
- No prior history of ectopic pregnancy: Though not an absolute contraindication to IUD use, this is stated as a relative contraindication on the C-IUD package insert.
- No history of or suspected hormonally-dependent neoplasm or undiagnosed abnormal vaginal bleeding: Both of these clinical scenarios are contraindications to IUD use. Any undiagnosed abnormal vaginal bleeding must be assessed prior to consideration for the study to rule out genital tract neoplasia. Any breast mass must be similarly evaluated to rule out carcinoma before opportunity for study entry.

[1] Though there are no data regarding LNG IUD expulsion following postpartum insertion, data on the C-IUD indicate that expulsion rates are significantly lower if insertion is performed at least 6 weeks postpartum.[48]

- Local language fluency and comprehension: Participants must speak one of the languages commonly used in Cape Town populations of recruitment (English, isiXhosa) and must be able to comprehend the risks, benefits, and obligations of the study to provide IC, as determined through the informed consent comprehension checklist score.
- Not participating in any other clinical trial with a biomedical intervention.
- Have no condition that, based on the opinion of the Site PI, would preclude provision of informed consent, make participation in the study unsafe, or complicate interpretation of data.

In addition to these criteria, women with any of the following conditions have medical contraindications to IUD use and are therefore ineligible: known congenital or acquired uterine anomaly including fibroids distorting the uterine cavity; known acute liver disease or hepatic neoplasm; known copper storage disease; and known hypersensitivity to any component of the LNG IUD or C-IUD. These conditions are specified on the package inserts of the Mirena® (Bayer Health Care Pharmaceuticals, Montville, NJ) LNG IUD and a copper T-380A IUD currently approved for use by the Western Cape Province, such as the SMB Copper T-380A IUD (SMB Corporation of India, Mumbai, India) or the Nova-T copper T-380 IUD (Bayer Pharmaceuticals, Germany) and WHO medical eligibility guidelines, thus are relevant to the clinical setting.[35]

Latex allergy is not a contraindication as nitrile gloves will be used during clinical procedures for participants with a latex allergy and no clinical product contains latex. Female condoms will be provided to participants with latex allergy as they are made of non-latex materials.

The criteria for which self-report is sufficient will be stated fertility intentions, intention to live/stay in the Cape Town area for the next 30 months, willingness to participate and ability to provide informed consent, local language fluency, participation in another clinical trial with a biomedical intervention, and history of infertility/sterility.

Clinical record review and/or participant report will be used to record last CD4 count or plasma VL result, confirm HIV diagnosis, and record last cervical cytology report within the last 3 years, per SA national care guidelines, as available.[49] For potential participants for whom HIV status cannot be confirmed in records, VCT services will be engaged for rapid diagnosis. Potential participants and participants with abnormal Pap smear results who have not received colposcopy will be counseled on need for this diagnostic examination and referred to the appropriate provider within the Desmond Tutu HIV Centre system. Potential participants with no recollection or record of cervical cytology will be evaluated for gross lesions at screening examination; those with lesions will be referred to the appropriate provider within the DTHC system. Women with no lesions on gross examination will receive a Pap smear at the time of a scheduled visit and abnormal results will be referred appropriately, regardless of their participation.

All potential participants, including those amenorrheic secondary to injectable hormonal contraception, will have urine pregnancy testing at both screening and enrollment/insertion visits to rule out current pregnancy.

Participant report with secondary record confirmation will be used for age, conditions contraindicating IUD use (e.g. hormone-dependent neoplasm), ART use, and recent pregnancy.

Enrollment will be delayed for PID or endometritis in the last 3 months or acute cervicitis or vaginitis. Postpartum women who initiated ART during pregnancy will be eligible for enrollment at 6 weeks postpartum if they have continued ART use and have a record of their most recent plasma VL<1000 copies/mL. Postpartum women electing not to continue ART will be deferred until 6 months post-ARV. Women using injectable contraceptives (DMPA or NetEn) at the time of recruitment may complete the chart eligibility screening but will have the final screening visit with testing and, if eligible, enrollment visit deferred until the time the next injection is due to minimize any baseline progestin effect. Women using oral contraceptive pills will have enrollment delayed to the placebo week of their pills for similar reasons and to minimize breakthrough bleeding symptoms.

A urine pregnancy test will be performed prior to IUD insertion for all women, including those with amenorrhea presumed secondary to injectable hormonal contraceptives. Pregnancy testing will be repeated at the insertion visit, which may be delayed until one week post-menses for women not using injectable hormonal contraceptives.

Though the package inserts of the study IUDs recommend that women have at least one child before IUD use, IUD use has been demonstrated to be safe and acceptable for nulliparous women.[51,52] As such, this study will not use parity as an eligibility criterion.

7.2 Recruitment

Women will be recruited from public sector clinics around Gugulethu and Nyanga where Desmond Tutu HIV Centre (DTHC) is active (including voluntary counseling and testing points, ART clinics, postnatal clinics following HIV-exposed infants, and other services), through community-based groups, and by provider referral over 38 months. Site recruiters will approach female patients aged 18-40 years after visit completion. Women agreeing to a brief study presentation will go to a private room for information and initial screening. Clinic patients routinely present for care at six-month intervals; we estimate 1400 women will be approached and 1000 will be screened.

Women declining to hear about the study will be simply enumerated with no additional information collected. Women who agree to receive information about the study in a private room from a study staff member and then declare lack of interest in the IUD or trial participation will be asked to complete a short verbal informed consent (IC) form and anonymous questionnaire for decliners able to provide informed consent. The decliners questionnaire will briefly assess socio-demographic characteristics, fertility intentions, and contraceptive use history and preferences. The IC process for decliners will thank potential participants for their interest and ask if they are willing to provide basic information about themselves like age and contraceptive method use history to allow investigators and collaborators to understand whether women declining trial entry differ significantly from women who proceed to enter the trial. The anonymous nature of this data collection will be explained during this session as will the use of verbal consent to ensure anonymity. The rationale for this assessment is to determine which groups should be targeted in efforts to expand IUD use, following compelling safety data. Women interested in trial entry

will receive the screening IC and a detailed study overview. This process will occur at outlying clinics only if a private space is available.

7.3 Retention

Once a participant is enrolled in the study, the study team will make every reasonable effort to retain her in follow-up, in order to minimize bias associated with loss to follow-up. The study team will track retention rates and address any issues related to retention.

To minimize loss to follow-up, as well as to improve adherence to visit schedules, participants may be contacted by study staff between follow-up visits. These contacts may be in many forms including: home visits, telephone, and in-person reminders of scheduled visits.

If a participant fails to appear for a scheduled visit, study staff will contact her as soon as possible. All attempts to contact a participant will be documented in the participant's study file. A participant will not be considered lost to follow-up until the 24 month eligibility period has finished, regardless of point at which last visit occurred. In addition, study staff must attempt to contact participant at a minimum of three times and a maximum of nine times since her last missed study visit before attempts to contact her for a specific visit are abandoned.

8.0 STUDY PRODUCTS

IUDs are T-shaped devices inserted directly into the uterus in a simple office procedure which act through a variety of mechanisms to prevent pregnancy for 5-10 years duration, depending on IUD type. Study devices will include the LNG IUD and the C-IUD. Both products are registered in South Africa. No ART medications will be provided through this study; participants will continue their routine care through the GCHC with ART initiation and/or provision through that established care program. Medications for treatment of STIs detected at screening and for treatment of incident STIs at interval measures for the participant and her partner(s) will be provided through the study and are donated by the South African government.

The Mirena® LNG IUD has been donated to UCT in South Africa from Bayer Healthcare, the manufacturers of Mirena®. The Mirena® IUD consists of a T-shaped polyethylene frame with a steroid reservoir containing 52 mg levonorgestrel packaged with a sterile inserter.

The C-IUD will be Model TCu 380A used in public sector services in Western Cape Province, such as that manufactured by the SMB Corporation of India or by Bayer Pharmaceuticals. The South African government has donated the C-IUDs needed for use in this study. Each T-shaped plastic frame has 176 mg of copper wire coiled around its vertical arm and a copper collar of 68 mg of copper on each of its transverse arms for a total copper surface area on the device of 380 mm^2.

Both the TCu 380A and Mirena® IUD will be sterile and will be inserted as described in the package insert.

The MC will be the Instead MC manufactured by EvoFem (San Diego, California) and is an FDA-approved menstrual management product. The Instead MC is made of plastic and is latex-free, packaged individually wrapped in boxes of 14 units. Participants will be shown the MC during the consent process and those enrolling in the study will be instructed in insertion and removal by study staff.

8.1 Packaging and Labeling of Study Product

Study product will be affixed with a label in accordance with regulatory requirements. Attention will be paid to the manufacturer's expiration date on each device.

8.2 Product Storage and Temperature

The study IUDs will be stored at ambient temperature 25˚C, with excursions permitted between 15-30°C. Packages will be protected from direct sunlight, water and mechanical damage.

8.3 Accountability

Complete records documenting receipt and dispensation, including dates and participant numbers, will be maintained. The study monitor will review the accountability logs. Disposition of unused supplies will be documented and completed only with permission from FHI 360.

For IUDs that are removed, they will be disposed of as biological waste through the system in place at GCHC. Participants will be counseled at study start that, should they experience a complete IUD expulsion, they should bring in the expelled device to GCHC within 72 hours of expulsion for proper disposal as described and for confirmation of type and device replacement, per participant preference.

The MCs used in this study are single use and will be treated as biological waste after completion of specimen processing.

9.0 STUDY PROCEDURES

The participants will make visits to the clinic for screening, enrollment, and up to five follow-up visits (3, 6, 12, 18, and 24 months post-insertion). A table summarizing study procedures is available in Appendix 1.

9.1 Screening visit

The potential participant will be provided introductory study information and the screening IC process will be conducted before any study procedures are begun. The screening process will be described to prospective participants, including potential risks and benefits of participation, in either an individual or a group information session initially. The session will also include a review of the study purpose and procedures and the major eligibility criteria. For group sessions, women interested in learning more about the study and potentially participating will be taken to a private room for an individual screening informed consent session with a trained study staff member at GCHC. If recruitment is performed at an off-site clinic, women will receive screening IC only if a private room is available. Those women consenting will then have chart review and complete the screening eligibility checklist with the staff member; if still eligible, they will be provided with a screening appointment at GCHC to complete the screening visit testing. If no private room is available at recruitment sites, potential participants will be provided appointment cards for GCHC and will complete screening IC there.

Staff will begin the IC process by assessing the literacy of the participant. Literacy will be assessed by asking the participant to read the introduction paragraph of the IC form. If a potential participant is unable to do this, an impartial observer not employed by the study will be asked to be present for the consent process

and sign as a witness of the consent. Comprehension of the study purpose and role and responsibility of the participant will be assessed with the informed consent comprehension checklist.

The screening visit will occur on the day of screening IC with enrollment visit and IUD insertion following within 28 days, according to participant preference and available clinical space. An exception to this interval will be for women using hormonal contraception. Women using injectable contraception (DMPA or NetEn) will be asked to return at the time that their next injection is due, to minimize effect of exogenous progestins without compromising contraceptive coverage. Similarly, women using oral contraceptive pills will be asked to return at the time of their monthly withdrawal bleed (placebo week) for IUD insertion. Also, women who are menstruating at the time of any appointment will be asked to return after bleeding has stopped to ensure accurate STI testing and ability to perform genital tract sampling for VL. To prevent this, participants will be called 24 hours prior to their appointment as a reminder and to also query whether they are experiencing bleeding. Women experiencing bleeding will be re-scheduled. Women will be asked to abstain from putting anything in the vagina for 3 days prior to a study visit. For eligible women who do not present for enrollment within the prescribed time period, repeat IC and eligibility screening will be performed; repeat CD4 count will only be done if the last count is >3 months from re-presentation for pre-ART women.

During screening, participants will complete a clinician and interviewer-administered eligibility form detailing relevant medical history, concurrent medications, fertility intentions, a chart review or, if unable to document with chart review, a rapid test for confirmation of HIV status and contraindicated conditions, and most recent CD4 and cervical cytology results. Cervical cytology examinations are performed every three years for HIV-positive women, per South African provincial clinical protocol. All women receiving care at the GCHC for HIV receive cervical cytologic screening and there is an algorithm in place for colposcopy referral as needed within the standard care system. Women who are eligible and consent to participation will receive cervical cytology screening per provincial guidelines as part of their routine HIV care outside the study. Participants deemed eligible to this point will then have an examination and testing for pregnancy, CD4 count (pre-ART only), and STIs (*T. pallidum* (TP), *N. gonorrhea* (NG), *C. trachomatis* (CT), *T. vaginalis* (TV), and *bacterial vaginosis* (BV)). The questionnaire will include a risk assessment tool for IUD insertion previously validated in Africa (Appendix 2).[53] Participants with mucopurulent cervicitis on exam, or with reactive STI (rapid TV, or NG/CT by PCR test) or BV test results will have the enrollment visit and IUD insertion deferred until two weeks but no more than four weeks following treatment, being the other exception to the 28 day interval.[54] Participants with evidence of active syphilis infection (reactive rapid test and RPR titre≥1:4 may proceed with enrollment but must initiate treatment following diagnosis. Treatment may be initiated at the time of enrollment visit.

The gynecologic examination will evaluate uterine position, PID signs (e.g. cervical motion tenderness), pelvic neoplasm (e.g. adnexal mass or inappropriately enlarged uterus), and specimen collection for STI laboratory tests. OSOM Trichomonas and BV Blue (Sekisui Diagnostics, San Diego, CA) point-of-care tests will be used for TV and BV.[54,55] TP will be tested with Determine Syphilis rapid test (Alere Inc, Waltham, MA) using whole blood, with *T. pallidum* hemaglutination assay (TPHA) for confirmation for a first reactive rapid test and rapid plasma regain (RPR) titre performed for clinical guidance. Baseline cases will be treated

with the protocol for TP of unknown duration for RPR≥1:4, while incident cases (determined based on trend in RPR measured every 3 months after successful completion of therapy) will receive single dose treatment; all participants treated for TP will be monitored quarterly for treatment failure as appropriate for HIV-TP co-infection.[54] The GeneXpert CT/NG® (Cepheid , Sunnyvale, California) or similar Polymerase Chain Reaction (PCR) assay will test for NG and CT. Participants with cervicitis or PID on exam or positive STI screening tests will be treated according to national guidelines.[55] Treatment for partner(s) will also be provided to the participant for NG, CT, or TV. This has been found acceptable with high rates of male partner medication use directly observed by the female patient in South Africa.[56]

At the screening visit and at every visit, study staff will provide individual risk reduction counseling to women to reduce risk of HIV transmission to male partners and reduce STI risk for both the participant and her partner. Women will be specifically informed that the IUD does not protect them from STIs, including HIV re-infection, and that condoms are the best source of protection both for them and for their partner(s). Women will be invited to ask questions regarding negotiating safer sex and will receive printed information resources already available to all patients at DTHC. Male and female condoms will be provided at every study visit during the trial. All women screened with documented HIV infection and not using ART will be offered referral for ART initiation with their regular HIV care provider, per local guidelines.

Women ineligible due to cervical dysplasia, suspected pelvic neoplasia, or pregnancy will be referred for care. Women with clinical signs of PID will not be immediately enrolled but referred, treated, and eligible for repeat screening 3 months after treatment.[46] Women desiring contraception but declining or ineligible to participate will be referred to affiliated public clinics where family planning services are provided at no cost. Women ineligible or declining to participate and not using ART will be referred for ART at neighboring clinics and encouraged to return at a later date when eligible for ART arm consideration, if still interested in the study and enrollment still open.

9.2 Enrollment and insertion visit

The enrollment and insertion visit will consist of review of screening laboratory results, repeat urine pregnancy testing including for those women experiencing amenorrhea secondary to injectable hormonal contraception, randomization, MC instruction and insertion, phlebotomy for VL and full blood count, speculum exam, genital tract sampling for VL and cytokine measures, IUD insertion, counseling regarding symptoms and warning signs of IUD expulsion and PID, and safer sex counseling. An opaque sealed envelope containing the coded study arm assignment will be opened by the study representative at the time of randomization. The envelope contents indicate arm assignment. A form will be kept to record dispensing the IUD designated by arm assignment by the study coordinator with secondary confirmation by a study nurse. For the ART-using study arms, the existing block randomization scheme, stratified by age and recent exposure to injectable contraceptives, will be maintained. However, women using ART will be given randomization envelopes sequentially from the last envelope (100th) moving backward numerically, while pre-ART women will continue to be given randomization envelopes in sequential order from the first. Block randomization was used, which will ensure that the treatment arms will be close to equally distributed between pre-ART and ART-using women.

Following urine pregnancy testing and randomization of eligible participants, participants will be provided with a disposable MC and asked to insert it in the restroom after explanation of the insertion process. Women will then be asked to attend a session for baseline questionnaire administration and undergo phlebotomy for plasma VL and blood count. This process will take approximately two hours. Following completion of these activities, the participant will have a pelvic examination where the MC is removed by the nurse practitioner. A waiting nurse will place the cup with the sample into a storage container in a refrigerator on site until transported to UCT medical microbiology laboratory. All samples will be stored on site at 4° C and then transported to the Medical Microbiology laboratory for processing and storage on the same day. Genital tract specimens will be stored at -70° C and testing performed in batches at regular intervals for efficiency. The nested study comparing sampling procedures was complete in April 2015; all participants with visits following approval of V7.0 will have MC sampling for HIV RNA VL and one endocervical swab taken for additional future testing (e.g. HSV2). Funding for some additional testing has been obtained and the investigators will proceed with the additional testing of residual specimens and the aforementioned endocervical swab samples. Residual specimens, including plasma from enrollment and remaining menstrual cup samples from all visits, will be stored at -70° for further testing (including but not limited to immune mediator and sexually transmitted pathogen testing) for participants who have consented to allow additional testing of stored samples in their Trial Entry ICFs, which has been present in all Trial Entry ICF versions used with participants. Funding has been obtained to conduct mucosal cytokine panel and HPV subtype testing on ECS (HPV) and residual MC samples from ART-using participants at enrollment, 3, and 6 month visits (enrollment and 6 month visits for HPV). For this testing, we will generate a list of ART-using participants based on having residual MC and ECS samples for the first three visits and having indicated in the Trial Entry consent that additional testing of stored specimens was permitted. It will not be necessary to recall participants for consent as permission to use residual specimens was marked at the time of Trial Entry ICF. We will randomly select 50 women from this list and test the selected samples. These samples and the first 50 pre-ART participants, who have already had cytokine and HPV analysis as part of the sample comparison sub-study, will all be tested for HSV-2 viral RNA with nucleic acid amplification assays. Based on results and availability of funds, this comparison between groups may be similarly extended within pre-ART and ART arms.

All IUD insertions will be performed by the study nurses and designated medical officers to preserve blinding of the PI and collaborators involved in data analysis and interpretation to IUD type. The FHI 360 Medical Monitor/Project Leader or designee will ensure clinical competency of the nurses for IUD insertion prior to participant recruitment.

Following insertion, participants will be counseled on infection or device expulsion symptoms and signs, need for continued condom use, and instructed to call the study coordinator with any questions.

9.3 Follow-up visits

Scheduled clinical follow-up visits (3, 6, 12, 18, and 24 months):

Participants will return for scheduled follow-up at 3, 6, 12, 18, and 24 months post-insertion. Visits will consist of: interval questionnaire completion (including partner information, general physical health, and contraceptive satisfaction using a modified version of the ORTHO birth control satisfaction assessment tool

(ORTHO BC-SAT), included as part of the interval questionnaire); symptom assessment; refresher counseling on STI/PID or expulsion symptoms and abnormal bleeding, including prolonged excessive bleeding (prolonged excessive bleeding, defined as more than 7 days soiling more sanitary products in one day than would be expected during one day of a normal menses); refresher safer sex counseling and need for continued consistent condom use; pelvic examination with IUD placement confirmation, pregnancy testing, genital tract sampling for HIV VL and ECS for storage and interval STI sampling (at 3, 6, 12, 18, and 24 months); and routine laboratory measures (Appendix 1). STI specimen collection will be done at 3, 6, 12, 18, and 24 months to determine impact of progestin exposure on incident STI and how STI relates to genital tract shedding with and without progestin exposure. Syndromic management, as per national guidelines, and, where possible, point of care tests, will be used at interval visits to diagnose and treat for vaginitis/cervicitis.[55] NG and CT test swabs will be collected and tested as possible, with asymptomatic infections treated, with the remainder frozen for testing at a later date . Endocervical sampling (ECS) will be obtained at each interval visit and frozen in media, which, along with residual MC and other specimens, will be reserved for future testing (including but not limited to HSV-2, HPV, and mucosal cytokine measures); the HSV-2, HPV, and cytokine testing will be undertaken with obtained supplemental funding for randomly chosen participants and may be extended pending results and receipt of additional funding. Participants with cervicitis or PID on exam or positive STI screening tests, where performed, and their partners will be treated as previously described for the screening visit.[55,56]

Study staff will meet participants at each visit for safer sex counseling and discussion of any concerns.

At each follow-up visit, study staff will review the participant's records for most recent plasma VL (ART-using) or CD4 count and new-onset AIDS-defining illnesses (e.g. tuberculosis) (pre-ART). Records of ART-using patients will similarly be reviewed for newly detectable VLs. The study will test plasma VL at every scheduled follow-up visit and, for pre-ART participants, CD4 count every 6 months. Pre-ART participants at any visit will be referred for free public sector HIV care and treatment services in the community operated by the provincial government with support from DTHC and according to local guidelines. Similarly, following local guidelines, ART-using participants with plasma VL>1000 copies/mL will be referred for clinical assessment and further management by their ART clinic. The Site PI and Site Co-Investigator are closely involved in the coordination of ART services in and around Gugulethu and routinely refer ART-eligible individuals for initiation of therapy. IUD removal will not be pursued in these participants unless there is evidence of pelvic infection.

9.4 Closing visit (24 months)

The final visit will comprise the interval questionnaire, pelvic examination and genital sampling, STI testing, and serology as described for the interval visits. Participants will also be asked whether they wish to retain the IUD and a brief exit questionnaire will be administered following completion of study activities regarding overall satisfaction with the IUD and/or the current contraceptive method, as applicable, and with the study. Participants wishing to retain the IUD will be counseled on the duration of efficacy for their assigned product and advised to return to a comprehensive clinic offering IUD services within the national health program with any problems or at the time removal is desired. The C-IUD is offered as part of the national formulary for contraception at no cost and removal visits will incur the same expenses as any other

health care visit within the public system. At this time, the LNG-IUD is not available within the public sector in South Africa. It is not within the financial capability of the study to provide the LNG-IUD at study exit.

Participants with any ongoing AEs at time of study exit, at the 24 month visit or by voluntary request to terminate participation or incident pregnancy, will receive appropriate care referrals and follow-up phone calls to 30 days post-study exit to ensure resolution and appropriate care receipt for AEs. Participants not within the 24 month visit window at study end and coming for an unblinding visit will have AE follow-up at that visit and receive a referral form at that time for any ongoing AE. Participants may also be asked to visit the study clinic post-study exit if reporting a new AE possibly related to the current/recent IUD use or experiencing worsening of pre-existing AE. Conditions not resolved after 30 days post study exit, defined as the last 24 month visit conducted on June 1, 2018, will be referred as appropriate.

Genital tract and plasma specimens of consenting participants with the sole identifier of study number will be stored at University of Cape Town (UCT) for use in future studies.

9.5 Pre-24 Month/Post Clinical Follow-up Exit Visit

Participants who are not yet eligible for or do not present for their 24 month visit at the time of site clinical activity completion (June 1, 2018) will be called to the site clinic to be unblinded to IUD type. Performing unblinding in the clinic will permit women who wish to discontinue their IUD to have the IUD removed at that visit. These women will also receive pregnancy testing, be offered STI testing and, for those discontinuing their IUD, request to complete an exit questionnaire and offer of a different contraceptive method or referral (for implant and surgical sterilization), as desired. Women who cannot come to the clinic by June 30, 2018 due to extenuating circumstances, such as temporary relocation to another part of the country, may be unblinded and appropriately counselled by telephone at the discretion of the study manager and with confirmation of their full name and date of birth to confirm their identity. As of June 5, 2018, we anticipate there are approximately 20 women who are not yet eligible for their 24 month visit and a further 10 women who are eligible but did not present to the study clinic for their 24 month visit by the end of regular follow-up/exit visit services on June 1, 2018. These women would be the subset of the study participants offered the above-described visit.

9.6 Contraceptive Continuation Call

Participants who are not eligible for or do not present in time for clinical completion of their 24 month visit but instead complete the above-described unblinding exit visit and elect to retain their IUD will be asked if the study manager or trained designee may call them at the time of their 24 month visit. This call will ascertain continued IUD use. Participants will be asked if they are willing to receive such a call and, if so, will sign an informed consent form and re-confirm their telephone contact information. Upon entering the eligibility window for the 24 month visit, the study manager or trained designee will call the consented participant based on time and number preferences specified at consent, confirm identity and then administer the questionnaire. For women who have continued using the IUD, they will be asked standard questions regarding method satisfaction from the call questionnaire. For women who report discontinuing the IUD, they will be asked about the date and reason for discontinuation and their new contraceptive method, if any.

9.7 Product and Study Discontinuation and Incident Pregnancy

Participants may elect IUD removal at any time. Participants will be counseled to call staff with concerns and for immediate appointment for evaluation or removal upon request, irrespective of scheduled visits. A participant desiring IUD removal will meet with staff for a counseling session and will be asked to complete a brief discontinuation questionnaire detailing discontinuation reason(s), changes in key life factors (e.g. partnership), and symptoms or side-effects attributed to the IUD. Staff will counsel the participant on other available methods and assist with selection. The participant will then have a clinical visit with the nurse practitioner, who will address any remaining participant concerns, remove the IUD, and arrange for new method provision as desired. Last, the participant will meet a trained interviewer for the discontinuation in-depth interview (IDI).

Participants discontinuing IUDs will be retained in the study unless requesting to withdraw, and complete remaining follow-up visits with scheduled testing until the end of the study or until becoming pregnant. They will not be included in safety outcome analyses after discontinuation, but their retention will provide insight into alternate method preference and effects of IUD discontinuation on HIV progression measures. Participants who are lost to follow up will not be assumed to have discontinued their IUDs, which require provider removal. Sensitivity analysis will be performed to determine consideration of loss to follow-up for acceptability outcomes.

For women who wish to discontinue the study and discontinue the IUD, they will be scheduled for an exit visit at which the IUD will be removed with the discontinuation interview requested. These women will be offered another contraceptive method and released to routine care at the clinic. For participants who elect to discontinue study participation but wish to retain their IUDs, the study arm assignment will be individually un-blinded by an unblinded staff member after notifying the site PI and FHI360 Medical Monitor/Team Leader. The participant will be advised on IUD type and duration of efficacy of the assigned IUD, as well as need for routine care and string checks. At this time, the LNG IUD is not available within the public sector in South Africa. It is not within the financial capability of the study to provide the LNG IUD to any participant discontinuing the study and requesting the LNG-IUD. Once discontinued, a woman will not be allowed to re-enter the study. If this departure occurs within the recruitment period or within the first 3 months of the follow-up period, a replacement may be recruited.

Incident pregnancy is not expected during this trial; however, the possibility exists, particularly for women who discontinue the IUD. The diagnosis of pregnancy will be approached in two ways: pregnancies occurring following IUD discontinuation and pregnancies occurring with the IUD in place (including unrecognized expulsions). Women who elect to have the IUD removed will have counseling from study staff for an alternate contraceptive method, which will be provided through the GCHC. Pregnancy will not be treated as an AE for this group of women and they will complete the study visit at which the pregnancy is diagnosed before exiting the study. However, women who are diagnosed with pregnancy while still recorded as having the IUD will be treated as SAEs; management of these cases is described in Section 10.4.

9.6 Unscheduled Visits and Complications/Side-Effects

Participants will be counseled extensively on possible side-effects or bleeding changes that are typical with the IUD during the IC process for enrollment, including possible menorrhagia or prolonged excessive bleeding with the C-IUD, and will also receive counseling regarding PID and ectopic pregnancy warning signs and about abnormal bleeding at the end of each scheduled study visit. Participants will be provided with the study emergency telephone number for urgent medical issues that may be used at any time, day or night. Should a participant require an unscheduled visit, the appointment will be made through the study emergency number and the participant will receive reimbursement to the value of 150 Rand (approximately US$9.90 - 18.75).

Participants will also be counseled on signs/symptoms of possible IUD expulsion. Women presenting within 72 hours of expelling a device may have the IUD replaced. Women presenting after 72 hours will have a menstrual history taken; those either menstruating or within 10 days of menstrual day 1 may have the IUD replaced following a negative pregnancy test.[35] Women who are menstrual day 11 or beyond will be counseled to wait until their next menses and provided with another contraceptive method until that time.

Participants will be asked to call the study emergency number should they experience bleeding changes of concern, including prolonged excessive bleeding, defined as more than 7 days soiling more sanitary products in one day than would be expected during one day of a normal menses; heavy or malodorous vaginal discharge; any pelvic pain or discomfort; or other symptoms they find concerning. The clinical nurse practitioner and an OB/GYN specialist will be available for any triage-related questions for participants who report symptoms concerning for PID, ectopic pregnancy, or anemia.

10.0 SAFETY MONITORING AND ADVERSE EVENT (AE) REPORTING

10.1 AE Definitions

An AE is defined as any untoward medical or social occurrence in a clinical research participant which may have a causal relationship with the study product or study procedures. Any medical condition or laboratory abnormality with an onset date before the enrollment date is considered pre-existing and should not be reported as an AE. Pre-existing events, which increase in frequency or severity during the use and are possibly or definitely related to use of the study product, will be considered as AEs.

An AE does not include:

- Medical or surgical procedures (e.g. surgery, endoscopy, tooth extraction, transfusion); the condition that leads to the procedure is an AE;
- Pre-existing diseases or conditions present or detected prior to start of study drug administration that do not worsen or are not possibly associated with IUD use;
- Situations where an untoward medical occurrence has not occurred (e.g. hospitalization for elective surgery, social and/or convenience admissions).

Study site staff will document on study forms all AEs reported by or observed in enrolled study participants presumed related to study product use, regardless of severity. All AEs will be collected from the time the participant signs the enrollment consent through study exit and will be collected at each visit on the AE

form. All participants reporting an AE will be followed clinically, until the AE resolves (returns to baseline) or stabilizes. AEs that are ongoing at the time of the 24 month exit visit will be followed for up to 30 days after study exit and then, if not resolved, will be referred to a health care provider for further follow-up. Participants coming for an unblinding visit who are not within the 24 month visit window will receive terminal follow-up of ongoing AEs at that visit and receive a referral as needed at that visit, due to termination of clinical care at site. Participants unblinded by phone due to relocation or other reason will be requested to follow with their regular care provider.

10.2 AE Grading

AEs will be graded according to the following scale:

- Mild (aware of all the time, but able to do all activities)
- Moderate (must discontinue some activities)
- Severe (incapacitating, not able to perform activities)

10.3 AE Relationship to Study Product

The site PI or designee will determine the relationship of each AE to the study product using the following scale:

- Possibly related: Onset of the AE has a reasonable temporal relationship to study product administration or to trial participation and a causal relationship is not biologically implausible;
- Probably related: Onset of the AE has a strong temporal relationship to administration of the study product or to trial participation that cannot be explained by the participant's clinical state or other factors and a causal relationship is not biologically implausible; or
- Definitely related: Onset of the AE shows a distinct temporal relationship to administration of the study product or to trial participation that cannot be explained by the participant's clinical state or other factors or the AE occurs on re-challenge or the AE is a known reaction to the product or chemical group or can be predicted by the product's pharmacology.

10.4 Medical Management of AEs

As some participants will experience concerns between scheduled visits, all participants will be provided with a 24-hour study emergency telephone number. Urgent medical issues (e.g. pelvic pain with fever) will be immediately triaged by study clinicians and appropriate care initiated, with an OB/Gyn available to facilitate timely management of gynecologic issues. Standard reimbursement will be provided for unscheduled visits and transportation costs provided for walk-in visits. With permission of the participant, records from all non-study medical providers related to AEs will be obtained. The participant's consent for study staff to obtain information from non-study medical providers must be documented in her study file.

Pregnancy rates with both IUDs are < 1/100 women-years, but an unrecognized expulsion would potentially lead to conception as fertility returns rapidly following IUD removal.[48,57] If the device has been expelled as recognized by the participant, an unscheduled visit will ensue with immediate urine pregnancy testing. Participants with positive results will be referred for immediate ultrasound; those with intrauterine

pregnancies will be referred for appropriate government-provided services of their choosing. Women with ectopic pregnancies will be referred in consultation with an OB/Gyn to the hospital center for management. Incident pregnancy with the IUD in place may present with positive pregnancy test at routine visits with no symptoms or with symptoms of possible ectopic pregnancy or irregular vaginal bleeding. For asymptomatic cases, examination and ultrasound will be performed to determine location of the pregnancy, whether the IUD is present, and (if so) its location relative to a gestational sac. In cases where the IUD is in the uterus and an intrauterine sac is seen, the participant will be counseled on options for IUD removal and risk of miscarriage vs. retention and risks of infection, preterm labor, and septic abortion. Participants desiring removal and pregnancy preservation will undergo IUD removal and be referred for obstetric follow-up. Those electing to continue both the IUD and the pregnancy will be referred in consultation with an OB/Gyn for obstetric follow-up; those electing to terminate the pregnancy will have the IUD removed and be referred for appropriate care.

10.5 Serious Adverse Events

A serious adverse event (SAE) includes any adverse event that results in any of the following outcomes, regardless of association with the study product:

- Death;
- An immediately life-threatening event;
- Inpatient hospitalization or prolongation of existing hospitalization;
- A persistent or significant disability/incapacity;
- A congenital anomaly/birth defect; or
- An important medical event that, based on medical judgment, may jeopardize the patient or participant and may require intervention to prevent one of the outcomes listed above.

A life-threatening SAE means that the participant was, in the view of the Investigator or designee, at immediate risk of death from the event as it occurred.

The determination of an SAE is one situation for which breaking the randomization code for an individual participant to determine arm assignment at the Investigator level is acceptable; this matter will be determined on a case by case basis with input from OB/Gyn and HIV medicine specialists.

Potential medical SAEs for this study include PID and incident pregnancy with IUD in place (including unrecognized expulsions). For incident PID cases, participants will be treated according to the severity of their illness, with possible hospitalization determined based on standard guidelines and the clinical input of the head of OB/GYN services at the major secondary referral hospital in this area.[54,55] Participants will be given the option of IUD removal; data indicate that the clinical course of PID is not significantly different for women with an already-placed IUD.[35] We believe these potential SAEs will be quite rare based on IUD data among HIV-positive women, but protocols will be in place to triage and expedite care for urgent medical issues.[6,16,41]

10.6 SAE Reporting

The site must report all serious adverse events, including death due to any cause, to FHI 360 within 24 hours of becoming aware of an outcome that classifies an AE as an SAE. The Site PI or designee should complete a FHI360 SAE Report Form and fax/email it to:

RAQA Regulatory Associate
FHI 360
359 Blackwell Street, Suite 200
Durham, NC 27701 USA
Phone: 1.919.544.7040
Fax: 1.919.544.1308
SAE@fhi360.org

In caes in which an SAE Report Form cannot be faxed or emailed within 24 hours, the Site PI or designee may report the SAE via telephone; however, an SAE Report Form must be completed as soon as possible after the verbal report.

A safety management plan will be developed and approved prior to participant enrollment. The safety management plan will outline the reporting requirements to NICHD, all IRBs, and other regulatory bodies, as appropriate.

10.7 Data, Safety, Monitoring Board

An independent DSMB for this study will be convened. The details for the operation and responsibilities of the DSMB will be defined in a separate DSMB Operational Plan. The Operational Plan will delineate the membership list, procedures of the DSMB, anticipated analysis plan, and proposed stopping criteria. The anticipated analysis plan includes one interim analysis at completion of 50% of 6 month visits and the DSMB will receive a report annually with enrollment, retention, and SAE statistics by study arm. The DSMB report will be submitted to the IRB with each annual renewal. Blinding of the DSMB report will be determined by the DSMB at time of interim analysis review. An observer from NICHD will be present on DSMB calls.

10.8 Social Harm Events

Participation in the study could lead to social harms that may include loss of privacy, stigmatization, relationship difficulties, physical or verbal abuse, interference with gainful employment, and coercion. Social harms may be identified in the IDIs or by other study staff, including recruiters, receptionists, nurses, physicians, pharmacist, and others. All social harms (i.e., events potentially related to study participation) will be brought to the attention of the Site PI. Participants who report social harms will be referred to speak with a study staff member. The Site PI or designee will report the event on the Social Harm Event form to FHI 360 within 24 hours of becoming aware of the event and to the local IRB if required.

11.0 SAMPLE SIZE JUSTIFICATION & APPROACH TO ANALYSIS

The FHI 360 Medical Monitor/Project Leader will perform baseline descriptive analysis and Dr. Jones will perform the remaining primary analyses, with assistance and input from Dr. Hoover. The FHI 360 Medical Monitor/Project Leader is responsible for coordinating the analyses; the UCT data manager will provide the encrypted database to the study collaborators. Co-investigators and other collaborators may propose

additional secondary analyses in the future, which will require approval from the Steering Committee and determination of whether the data will be de-identified.

If a participant elects to withdraw from the study, all data contributed to that point will be included in the analysis.

11.1 Sample Size Justification

The study design is an RCT with participants and primary analysts blinded to IUD arm assignment. This analysis will use an intent-to-treat approach for the acceptability measures and an as-treated approach for the primary safety outcome. Sample sizes conservatively assume alpha=0.05 for two-sided hypotheses testing (although one-sided testing may be used for safety outcomes at the discretion of the DSMB) and give the minimal differences detected with 80% power.

To evaluate impact of LNG-IUD use on HIV genital tract shedding, the power calculation is based on comparing baseline and 6 month measures between LNG IUD and C-IUD users. Genital viral shedding is variable within person. For example, ART treated women who are shedding at one visit are very likely to not be shedding at the next.[10,14] If the LNG-IUD were to increase likelihood of genital shedding, it would shift this within-person dynamic towards greater probability to be shedding when a woman returns to visits.

For the pre-ART group, women (n=33/arm) should provide at least **30 evaluable** participants per arm at six-months follow-up. The assumptions being used are a 5% IUD discontinuation rate and a 5% loss to follow-up rate at six months. Within this group of women, we assume that 60% of pre-ART women with the C-IUD will have viral shedding at the enrollment visit, based on MC collection data from measures of the first 47 participants in this study with sufficient sample volume. Based on a cross-sectional two-sample exact test, we can detect at least a 33.2% difference in presence of viral shedding between the two study arms in women not taking ART (power=80%, two-sided α=0.05).

For ART-using women, at least 100 women total (n=50 per arm) should provide at least **45 evaluable** participants at 6 months of follow-up, based on the same loss to follow-up and IUD discontinuation rates. We assume that 22% of women using ART will have genital HIV RNA detected at a given visit.[10] Based on a cross-sectional two-sample exact test, at least a 27.7% increase in viral shedding can be detected between the two study arms in women taking ART (power=80, two-sided α=0.05)

For a pooled sample analysis, **75 (45 + 30) evaluable** subjects per arm should be available at 6 months with an estimated presence of viral shedding in C-IUD recipients of 37.2% (60% * 30/75 + 22% * 45/75). Based on a cross-sectional two-sample exact test that conservatively does not adjust for ART use, a difference of at least 24.0% in the presence of viral shedding can be detected between the two study arms (power=80%, two-sided α=0.05).

For the secondary safety outcome of plasma HIV VL at 6 months in pre-ART participants, there will be sufficient power (>80%) to detect at least a 0.55 log difference in mean change in plasma VL between treatment arms (n=30 evaluable participants/arm) at 6 months using ANCOVA based on a standard deviation of $\log_{10}$ VL of 1 and an intraclass correlation of at least 0.5 between repeated measures from the same persons.[58-62] As the number of pre-ART participants has substantially decreased, current interim

analysis plans for this safety outcome will be discussed with members of the Data Safety and Monitoring Board (DSMB) and their input sought on the best approach to safety analysis.

Table 1. Minimal detectable differences in mean VL between arms of variable size assuming 5% loss to follow-up and 5% IUD discontinuation at 6 months (n=33-44/arm; two-sided α=0.05, Power=80%)

Proportion IUD discontinuation	Minimum detectable difference in $\log_{10}$ VL
30/arm at 6 months	0.55
35/ arm at 6 months	0.51
40/ arm at 6 months	0.48

For the acceptability outcome, IUD continuation through 24 months, we are considering this outcome as a pooled analysis regardless of ART use and believe a 50% minimum continuation rate is necessary for method feasibility in any setting. However, we hypothesize the LNG IUD continuation rate will be much higher and exceed that of the C-IUD, reflected by one-year continuation rates of 70-93% of the LNG IUD among HIV-negative populations in industrialized countries.[63-65] Table 2 presents minimal detectable differences in continuation of the LNG-IUD at 80% power based on 50 – 60 % continuation of the C-IUD and at least 67 evaluable participants at 24 months per arm. The 67 evaluable subjects assume a 10% annual dropout from the original sample of 83 for whom we will not know if they have continued using the IUD device. Thus, the proposed sample size for the primary safety outcome measure has the ability to detect a difference of at least 25% between continuation rates of the two IUDs with power of 80% (Table 2).

Table 2. Statistically detectable LNG IUD continuation for varying C-IUD continuation rates at 24 months at 80% power with loss to follow-up/censoring assumptions based on 10% annual loss to follow up and two sided testing with Type 1 error = 0.05

Arm size	C-IUD Arm Continuation (P0)	Detectable LNG IUD Arm Continuation (P1)	P1-P0	Power
67	.50	≥.75	≥.25	.811
67	.60	≥.84	≥.24	.836
77	.50	≥.73	≥.23	.797
77	.60	≥.82	≥.22	.815
86	.50	≥.72	≥.22	.806
86	.60	≥.81	≥.21	.820

11.2 Primary Outcome Analysis

The primary safety outcome, genital tract HIV shedding, will be measured by comparative change in genital tract VL detection from baseline to 6 months for women that use the LNG IUD vs. C-IUD over this time period. HIV RNA genital shedding, a surrogate for HIV infectivity at 6 months, will be measured as a binary outcome (Detectable, Undetectable). This will be compared separately for pre-ART and ART-using women

with Mantel-Hanszel stratified odds ratios of [IUD Arm Usage x genital HIV RNA detection], stratified in each group by any detectable genital VL (vs. undetectable) at baseline.[66] We will also combine these comparisons between ART and pre-ART participants by pooling all observations together in a Mantel-Haenszel odds ratio that stratifies by both baseline genital VL detection and ART use. Additional analyses of repeated post-baseline measures with detectable genital VL will use GEE with a logit link function, including baseline genital VL detection, current ART use, and treatment arm assignment as predictor covariates and an independent working correlation structure.[67,68] Time to first detected genital shedding will also be compared between study arms by Cox proportional hazards models (including other covariates such as time dependent ART use). If many specimens show detectable genital VL levels, then two stage models will be considered assessing shedding presence and quantity (on the $\log_{10}$ scale) of genital VL. The first stage will compare probability of having detectable genital VL between the treatment arms as a binary outcome (exact tests, logistic regression). The second stage will compare mean value of $\log_{10}$ genital VL among those with detectable levels as a continuous outcome (linear models). These models will be adjusted for presence of viral shedding prior to IUD insertion as a stratifying covariate.

11.3 Secondary Outcomes Analysis

For the secondary safety outcome of plasma HIV VL in pre-ART women, HIV progression will be measured by comparative change in $\log_{10}$ plasma VL from baseline to 6 months for women that use the LNG IUD vs. C-IUD over this time period. Unless women using ART are unexpectedly found to have significant levels of detectable plasma VL, they will not be included in this analysis. For a "baseline value only adjusted analysis", we will use an ANCOVA approach or equivalently constrained longitudinal data analysis (CLDA) that effectively incorporates the baseline (pre intervention delivery) level of plasma $\log_{10}$ VL and treatment arm assignment as predictor into a model with the 6 month VL as the outcome.[69,70] Expressed mathematically in the ANCOVA form

$$Y_i = a + bX_i + cT + e_i \qquad (*)$$

Where i denotes the study subject, Y is the $\log_{10}$ VL measure at 6 months, X is the $\log_{10}$ VL measure at baseline, T is the treatment arm assignment (0 = C-IUD, 1 = LNG IUD) and e is independent normal error while a, b and c are constants. The null hypothesis is that c = 0 or that once baseline VL is adjusted for, there is no association between treatment arm assignment and 6 month VL measures. To confirm the baseline only value adjusted analysis, the comparisons in (*) will then also be adjusted by age and hormonal contraceptive exposure at baseline, which are stratification factors at enrollment, and other patient characteristics that are related to the outcome by adding a vector of these covariates Z with a constant parameter vector d to the right-hand side of (*). Stepwise selection models with treatment arm age group and hormonal contraceptive usage forced in and (two-sided) p-value to enter and stay of 0.10 for other variables will be used to obtain the final adjusted model.

In sensitivity analyses that focus on longer term plasma VL outcomes, repeat measure VL load outcomes across all study visits will be compared by arm using log10 VL measures from all time-points out to 24 months with mixed models or generalized estimating equations (GEE). In ANCOVA formulation for these analyses, model (*) expands to

$Y_ij = a + bX_i + cT + dZ_i + e_ij$ (**)

where j denotes time of VL measure (3, 6, ….., 24 months), the covariate vector Z now includes indicators of which monthly follow up visit the measure is from, but otherwise is time invariant and within the same person i, error e_ij is allowed to be correlated. Stepwise selection models with treatment arm forced in and p-value to enter and stay of 0.10 for other variables will be used to obtain then final adjusted model. If other time dependent covariates are included into adjustment, GEE models will conservatively use a diagonal working covariance to prevent estimation bias from within person correlated predictor variables.[67] Otherwise, models that allow for informative censoring by reaching CD4 ART initiation thresholds will be fit as for this and all other efficacy outcome analyses.[70,71] No VL measurements taken after a woman initiates ART will be included in these analyses. While the LNG hormonal effect "c" will initially be fit as time invariant, if descriptive analysis suggests a time treatment interaction, we will modify (**) to allow for this. For other safety outcomes for Aim 1, we will use Cox Proportional Hazards (PH) models to compare incidence rate of STI and PID infections between the two arms. We will use mixed models or GEE as described above to compare repeat longitudinal CD4 and hemoglobin measures between arms.

The secondary acceptability outcome, IUD device continuation, will be measured by proportional differences in IUD continuation at 24 months between study arms first using exact tests and then adjusted for other covariates using logistic regression. We will also compare time to discontinuation first using Kaplan-Meier curves with log rank tests and then using Cox PH models for adjusted comparisons, particularly if there is high study dropout before the 24 month end of follow-up.[72] In these models for women who discontinue the IUD, the time of event will be [date of IUD discontinuation – the date of insertion] and the event status will be 1=Yes. For women who are lost to follow up (or die) before 24 months, the time of event will be [last date known to be using the IUD – date of entry] and the event status will be 0=no. For all other women the time of event will be [date of 24 months visit – date of insertion] and event status will be 0=no. PH models will also be fit to adjust these comparisons for other baseline patient characteristics that could potentially influence discontinuation. Several prognostic baseline variables will be used in these adjusted models, such as age, partnership status, pre study entry contraceptive use, and fertility history. Stepwise selection models with treatment arm forced in and (two-sided) p-values to enter and stay of 0.10 for other variables will be used to obtain the final adjusted model. Change in partnership status, mean bleeding days or a coded trimester (120 days) of amenorrhea, method satisfaction score, and incident ART use may also be included as time-varying covariates in PH models to identify any mediating relationship of LNG IUD with device discontinuation should a main effect of IUD type be found.

For other acceptability outcomes, method satisfaction BC-SAT scores will be descriptively compared between arms using means, medians, and standard deviations. Rank tests, t-tests, and linear models will evaluate unadjusted and adjusted associations between study arms for cross sectional levels and changes in these measures. Trends in scores over time will be described with analysis of covariance (ANCOVA) and other mixed model (or GEE) approaches as needed. Covariates included in adjusted models will include hemoglobin, mean bleeding days, parity, partnership status, and age. In prior studies, BC-SAT scores have been close to normally distributed but if non-normal distribution is detected, transformations of this

variable will be considered.[73,74] Change in mean bleeding days/month and patterns will be measured by questionnaire entries. Cross sectional levels and changes in bleeding days will be quantified and compared using the same approaches as for satisfaction scores. However, since number of bleeding days is a count variable, variable transformations or use of a negative binomial rather than linear function will be considered as needed. A separate variable will be created for women who experience amenorrhea throughout an entire follow-up period and these women will also be considered in the analysis, using bleeding pattern categories, as defined by WHO.[75,76] For women who are amenorrheic at study start, changes in their bleeding patterns will be recorded as for other women but consideration will be given to stratifying the analysis for women who started the cohort period with amenorrhea secondary to injectable contraception, particularly since we will purposively enroll an equal balance of participants with exposure to injectable contraceptives in the last four months between arms. Descriptive analyses of bleeding patterns from the interval questionnaires since insertion of the devices will be made using median and mean days per month and trimester. Bleeding patterns by trimester since insertion will be quantified and compared between study arms using standard approaches and WHO definitions for amenorrhea and irregular, prolonged, frequent and infrequent bleeding.[75,76] While this analysis is exploratory, unadjusted and adjusted comparisons of levels and changes in these outcomes over trimesters will be made using exact tests, trend tests, Mantel-Haenszel models, logistic/conditional logistic and mixed or GEE models. Two other bleeding measures are included in this study: hemoglobin and the bleeding sub-section of the BC-SAT instrument. Trends in hemoglobin and BC-SAT bleeding scores will be similarly quantified with repeated measures ANCOVA, mixed or GEE models and compared to self-reported bleeding days or reported amenorrhea using the same procedures described for satisfaction.

11.4 Interim analysis

The originally-planned interim analysis was waived due to the change in primary outcome measure and reduced sample size. This change was reviewed and approved by the DSMB in July, 2016.

11.5 Qualitative analysis

Transcripts for discontinuation IDIs will be entered into Atlas-ti (ATLAS-ti Center, Berlin) for coding and analysis. Coding and categorization will be undertaken throughout data collection to allow a process of 'progressive focusing' to occur through interview topic guide revision as needed. Field-notes, based on recorder observations, will: inform interview technique and content revision, identify emergent areas of analytic interest, and be analyzed with interview data. Key areas for qualitative study include: family and community; sexuality; fertility intentions; sexual safety; health and perceived health risks; relationships; power; coercion; self and community perception; and economic situation.

A glossary of key terms and concepts will be generated. Interview transcripts will be coded with emergent themes systematically 'charted' by interview and line number as interviews proceed using a grounded theory approach.[77] Transcript coding will occur in three stages. Open coding will break the data into meaningful concepts and assign codes and categories. Axial coding will identify connections between categories. Finally, interpretive coding will identify emergent patterns between concepts and across accounts.[78,79] Quotes will be extracted to provide evidence for each theme and category. Qualitative analysis will adopt an inductive approach where emergence of key findings throughout inform the focus of

further investigation and analysis, including considerations based on different contexts of decision-making (e.g. age, infection duration).

Data from the IDIs are only collected at one time point and thus will be analyzed across participants.

12.0 RANDOMIZATION AND BLINDING

12.1 Randomization

Randomization will be done in six strata to ensure equal proportions in each arm. One stratum will be reserved for women recently (<120 days) exposed to injectable contraceptives (DMPA and norethisterone enantate (NET EN)) and those not exposed. These groups will be further divided into three age groups: ages 18-23, 24-31, and 32-40 years for 2 x 3 = 6 stratification groups total. Envelopes will be prepared with block randomization of randomly selected permuted blocks of 4 or 6. As we have already generated 600 randomization envelopes (100 per stratification group), we can maintain our current block randomization scheme, stratified by age and recent exposure to injectable contraceptives. However, women using ART will be given randomization envelopes sequentially from the last envelope (100th) moving backward numerically, while pre-ART women will continue to be given randomization envelopes in sequential order from the first. Block randomization was used, which will ensure that the treatment arms will be close to equally distributed between pre-ART and ART-using women.

12.2 Blinding

Participants and outcomes assessors will be blinded to IUD type received. None of the primary outcome assessors (e.g. the site PI, collaborators performing the analysis, or data collection or entry staff) will know which IUD was inserted in any specific participant. The study nurses will perform all IUD insertions and are separate from the study manager and coordinator; the study coordinator is a non-clinician who will not be performing pelvic examinations or IUD insertions. A list with arm assignment and IUD inserted will be kept in a locked file cabinet in a secure location accessible only to unblinded study staff and will be used at study closing visit to inform counseling on length of efficacy for women electing to retain the device. The PI and collaborators will not have access to this code unless breaking the randomization code is required based on SAE patterns or at the time of safety analysis, if one group is found to have a pronounced deleterious effect as compared to the other group. The PI and other collaborators involved in outcomes assessment (Drs. Jones and Hoover) will not perform any pelvic examinations on participants. The decision for breaking the code and potentially stopping the study will lie with the DSMB.

13.0 DATA MANAGEMENT PLAN SUMMARY

The FHI 360 Medical Monitor/Project Leader will lead questionnaire development and she or her delegate will be present at staff training at the data collection site to ensure accurate, consistent instrument completion. The FHI 360 Medical Monitor/Project Leader will be assisted by Dr. Jones of City University of New York (CUNY) on data quality assurance through site visits and development of a data management plan. Data entry and cleaning will be performed at the University of Cape Town. All data will be entered into password-protected databases and cleaned in South Africa and sent as an encrypted file by secure electronic transfer to the FHI 360 Medical Monitor/Project Leader and Dr. Jones. Equipment needed for

data transfer is limited to password-protected computers with secure internet connection and encryption software for electronic transfer of an encrypted database.

Data accuracy will be checked in three phases. First, the study coordinator or data manager will review all completed forms at the end of each business day for completion and inaccuracies requiring clarification. Cross-visit data checks will be performed routinely on participant charts as described in the data management plan. Data from questionnaire instruments will be entered in Cape Town, with checking and reconciliation of incorrect entries by the data manager. Laboratory results will also be entered into the database, and linked by the unique study number assigned to each participant at time of entry. A master list linking study number to participant name and contact information will exist in hard copy and encrypted electronic format. This list will be kept in a locked cabinet in the University of Cape Town study office with access limited to the site PI and the study coordinator. To protect participant confidentiality, the sole unique identifier will be the study ID number, which will not be associated with any UCT or South African national identifier or with the randomized treatment arm. The FHI 360 Medical Monitor/Project Leader will review the database for completeness with codebook and simple descriptive statistics. The Site PI and the study coordinator will be contacted if missing data or data believed to be erroneous are detected for cross-checking. The handling of missing data, except in cases of simple failure to complete electronic entry, will be determined on a case-to-case basis to ensure recall bias is not introduced.

Data collection forms used for this study may have data that are directly recorded on them and will, in cases, be considered source documents. Other source documents include, but are not limited to, staff journals/notes, medical notes, screening and enrollment logs, laboratory results, IC forms, participant reimbursement logs, in-depth interview transcripts and audiotapes. A detailed description of which documents are considered source documents for the trial will be developed.

13.1 Study Instruments

Separate questionnaire instruments for the screening, enrollment, each interval, and exit interviews will be developed and placed into a standardized format consistent with site standards. Questionnaire instruments will be developed in English and undergo study team and community advisory board (CAB) review. The CAB is a standing committee that meets at regular intervals to discuss all ongoing DTHC studies. The study and questionnaires will be presented to the CAB at this meeting for their input. The resulting pre-test version will then be translated into isiXhosa and back-translated by a different person to ensure fidelity. Instruments will be pre-tested on 20 HIV-positive women who are not participants but volunteers who will provide input to assess instrument comprehension and whether any contraceptive decision-making aspects are missing. Results from the pre-test and final CAB inputs will be integrated into instruments. This activity will precede recruitment and enrollment and is integral to ensuring instrument quality.

Hemoglobin measures will be used to validate bleeding pattern responses from questionnaires.

14.0 HUMAN SUBJECTS CONSIDERATIONS

14.1 Institutional Review Board Review and Approval

Before contact with potential participants, the study protocol and informed consents must be approved in writing by the Institutional Review Boards (IRBs) listed below:

FHI360 PHSC: FWA00000025
University of Cape Town: FWA00001637

No modification or amendment to the protocol will be implemented without prior IRB approvals. The study will be conducted in accordance with all conditions of approval by the IRBs. Written re-approval of the research by the IRBs is required at least annually.

Please refer to section 17.1 for procedures for emergency departures.

14.2 Informed Consent

Prior to initiating the IC session, study staff will assess the literacy of the potential participant by asking the woman to read the introduction paragraph of the consent. If the potential participant is not able to read this section, an impartial observer not employed by the study will be asked to be present to witness the consent process and will sign the IC form attesting to their role. At the beginning of the IC session, the study staff will describe the portion of the study involved in that specific consent, outlining all procedures and associated time commitments, duration of participation, risks and benefits of participation, and staff contact information. Following this description, each participant will complete an informed consent comprehension checklist and attain a score greater than 80% with no essential knowledge questions incorrect before providing written informed consent for screening and trial enrollment participation. Potential participants who fail the literacy assessment will have the comprehension checklist read to them and will provide answers verbally. Potential participants who do not achieve a passing score on the first attempt will be counseled on areas represented by the incorrect answers and permitted to repeat the comprehension checklist up to two more times. Women unable to achieve a passing score after three attempts will be excused from participation.

At screening, women interested in participation after receiving information about the study will be asked to complete IC for all screening procedures, including in-depth medical screening with medical record review and pelvic examinations with STI and pregnancy testing performed at the screening visit. Women completing the screening examination will be asked to return for results at the enrollment visit, as needed. Women will be provided the risks and benefits of the screening visit by trained study staff in a private room. The study coordinator will be available to address any questions or concerns. The screening IC session will be limited to screening only. We estimate that 1000 women will need to be screened to enroll the planned sample size of 166 women; recruitment and screening will continue through 2016 to allow at least 6 months active time in the study for all women while also providing maximal time to reach the desired sample size.

Women declining study consideration after private counseling during the screening IC process will be asked to complete verbal witnessed IC to preserve the anonymous nature of this component and the previously-described data collection form querying reasons for declining/ ineligibility and demographic and contraceptive use information. Women declining study entry after consenting to and completing screening and determined to be eligible, or women who are medically ineligible as determined at the time of the screening visit will be asked to complete the decliner/ineligible questionnaire, with the data linked to their screening ID number only. This session will take approximately 20 minutes and will occur at either the screening or enrollment visit.

All women who complete the screening examination will be scheduled for enrollment; however, those deemed ineligible by laboratory result before the enrollment visit will be contacted by telephone; those deemed temporarily (e.g. STI detected) ineligible will be provided treatment and invited to return in two weeks for continued evaluation for study participation. Medically eligible women who remain interested in study entry will be invited to the enrollment/insertion visit, at which point they will be asked to complete the informed consent comprehension checklist, and upon receipt of a satisfactory score, provide IC for study participation, undergo randomization, and proceed with study activities. Women declining trial entry, despite being medically eligible, will be thanked for their time and asked to complete a decliner/ineligible questionnaire; data limited to the screening visit only will be retained for analysis with regard to screening-specific outcomes, like accuracy of the screening instrument for IUD insertion. Women presenting for the enrollment visit will be asked to join a study staff member in a private room to review study information and their screening results. The IC process will review the purpose, the risks and benefits, and the required activities of the study, with emphasis on the equal chance of receiving either type of IUD and not being informed which type is provided unless the study collaborators or DSMB determine that unblinding is warranted based on interim analysis results. The potential participant must satisfactorily complete an IC comprehension checklist as mentioned previously prior to providing written IC.

For all IC forms, one copy of the consent will be stored in a secure and locked filing cabinet separate from other study documentation, and a second copy given to the participant to bring home.

At the time of IC, the trained study staff member will provide counseling on contraception, safer sex, and condom negotiation tools and will provide male and female condoms, information pamphlets, and referrals for reproductive health services, as needed. Women interested in receiving contraception but either not interested in study participation, withdrawing from the study at any point, or deemed ineligible during the recruitment/enrollment process will be referred for safer sex counseling, including provision of condoms, and contraceptive method provision within the DTHC clinic system.

14.3 Risks

The chief risks to participating in this study are: the potential for accelerated HIV shedding or progression with the LNG IUD (although we strongly believe this will not happen based on the evidence to date as explained in the background and rationale section); risks associated with IUD insertion, including uterine perforation, device expulsion, incident pregnancy (contraceptive failure), and PID; loss of confidentiality regarding HIV status or other personal information; discomfort with the personal nature of some questions;

possible physical discomfort with pelvic examination and IUD insertion; and physical discomfort and possible injury from venipuncture. Of these risks, potential serious adverse medical events are the most concerning but their risk is quite low. Participants will be notified of all possible risks during the IC process. An interim analysis will be performed to assess any negative effects of either IUD on HIV shedding or progression and/or excessive adverse events associated with either device, and this analysis will be reviewed by the independent DSMB.

Risk mitigation for complications associated with IUD insertion (e.g. uterine perforation) will involve the FHI 360 Medical Monitor/Project Leader, a board certified gynecologist, performing an IUD insertion training session for the study nurse-practitioner who will then be proctored for IUD insertions until clinical competency is achieved.

There is no reported risk associated with MCs. There may be slight discomfort if the MC is not inserted correctly. Clear instructions for insertion will be provided.

14.4 Benefits

Benefits to the participants include regular medical follow-up that provides STI evaluation and treatment, counseling and support, and ongoing laboratory monitoring to augment clinical care provided according to DTHC protocol. Study staff will also provide safer sex counseling and condoms, written health promotion material on safer sex, contraception, and, in event of IUD discontinuation, method options or birth preparedness; and referrals for appropriate obstetric care, gynecologic care including contraceptive provision, or to support groups for women interested in having more children, as applicable. This service will be provided at all study encounters, providing individual benefits to participants that may not be available in a busy clinic environment. The risks of the study are balanced by provision of the contraceptive device and counseling and referral services that may improve quality of life for participants.

Participants will be reimbursed to the value of 150 Rand (approximately US$18.75) at the completion of each scheduled study visit for compensation of travel and time and for unscheduled visits. Participants completing discontinuation IDIs will be reimbursed to the value of 50 Rand (approximately US$6.25).

14.5 Confidentiality

Every effort will be made to protect participant privacy and confidentiality. To safeguard confidentiality, each participant will be assigned a unique number code, which will be the only identifier on questionnaires and specimens. At the research site, the key to participants' code numbers and names will be kept in an encrypted file and a hard copy which will be locked in the project office separate from other study materials, and as a password-protected file for the screening and enrollment logs. Only the Site PI or study coordinator will be able to access this information. There will be an identification sheet with an individual participant's name and contact information, which is stored separately from the study chart. This information will be used to contact participants with lab results and for follow-up appointments. All other study documents will use the assigned code as the sole identifier. All study files, when not in use during interview or as previously described, will be kept in the locked file cabinet in the study office.

All computer files will be identifiable only by the participant's code number. Participants' names will never be used.

The following systems established for previous Division of Acquired Immunodeficiency Syndrome (DAIDS) funded trials will be used. All source documents will be maintained in locked study cabinets in locked rooms at the research site or the UCT. Data from questionnaires, clinical assessments and laboratory reports will be entered into a custom-designed Microsoft Access database by a data clerk. The study database will be maintained in a firewall password protected SQL-server with nightly backups. All study records will contain anonymous participant identification numbers and no participant names or identifiers will be recorded in the study database. All electronic files will be encrypted and password-protected.

For qualitative data, all interviews will be digitally recorded and uploaded to a password protected study computer for transcription and translation. The qualitative study team will systematically review transcripts throughout the collection period to ensure common interpretation of key terms and concepts. Experienced translators fluent in isiXhosa and English will transcribe all IDI data verbatim. To maintain confidentiality, access to all qualitative data will be restricted to researchers directly responsible for data collection and analysis. All personal identifying information emerging in interview accounts will be removed from final transcripts, with hard copies secured in locked cabinets. Electronic access to related computer files will be password protected, with files deleted following completion of qualitative analysis.

14.6 Alternatives

Women deemed ineligible or declining study entry will be referred for contraception and any condition requiring further investigation, as needed. Participation is completely voluntary, and the alternative is not to participate; non-participation will in no way impact the clinical care women receive at the clinic.

14.7 Community Involvement and Consultation

DTHC operates a DAIDS-recognized Community Advisory Committee (CAB) available for recruitment material and study instrument input. Established community mobilization efforts will incorporate messages about the IUD and, following IRB approval, the clinical trial.

14.8 Research Integrity

Concern in the United States about the quality of biomedical and behavioral research has led to the establishment of regulations and guidelines for handling the allegations of scientific misconduct. As a recipient of U.S. government funding, FHI 360 is required to develop policies and procedures related to scientific misconduct that conform to these regulations. The regulations define "misconduct" in science as a "fabrication, falsification, plagiarism, or other practices that seriously deviate from those that are commonly accepted within the scientific community for proposing, conducting, or reporting research. It does not include honest errors or honest differences in interpretations or judgments of data".

FHI 360 has specific obligations under these regulations with respect to the handling of information related to scientific misconduct that may come to its attention. In addition, the regulations require that FHI 360 establish procedures related to initiating an inquiry, pursuing an investigation, and informing a specified circle of authorities should the situation warrant it. FHI 360 will pursue all allegations of misconduct in

research or questionable academic conduct that may raise legitimate suspicions of misconduct, and will conduct inquiries and investigations to resolve questions regarding the integrity of research. In conducting inquiries during an investigation, FHI 360 will focus on the substance of the issue and will be vigilant not to permit personal conflicts between colleagues to obscure the facts.

14.9 Study Discontinuation

The study may be discontinued or suspended temporarily at any time by the funders, IRBs, local regulatory authority or the Protocol Team (e.g., in response to recommendations from the DSMB). Stopping rules will be determined by the DSMB; however, probable stopping criteria include the following:

The stopping criteria we propose for futility include: 1) Inability to recruit all planned participants into each study arm within a 36 month period, and 2) Rates of IUD discontinuation and/or loss to follow up and withdrawal above 50% in the LNG-IUD arm or in both arms combined prior to 12 months. The benchmark for recruitment will be cohort completion within a 24-month period.

The stopping criteria we propose for safety are: 1) a statistical difference in mean $\log_{10}$ VL change of HIV viral RNA shedding between study arms at the interim analysis using the Pocock procedure, but the unadjusted p-value of 0.05 will be used for the final analysis; 2) a significant difference in mean CD4 count decrement between study arms at interim analysis; 3) a significant difference in number progressing to ART eligibility between study arms at the interim analysis; and 4) number of serious adverse events thought to be possibly, probably, or definitely associated with participation occurring in one or both study arms to warrant stopping in expert opinion or by IRB decision.

If the study is stopped before the planned end, site staff will make every attempt to contact each participant remaining in the study at the time of study closure for a final follow-up visit.

15.0 CLINICAL MONITORING SUMMARY

Site monitoring will be performed by Westat as contracted by the donor, NIH. Site visits by clinical monitors will be made in accordance with Westat policy and SOPs. The purpose of clinical monitoring is to assure the quality and accuracy of data collected and entered in the database, to determine that all regulatory requirements surrounding clinical trials are met, and to ensure that the study protocol is being followed as written.

The Site PI will allow the Westat clinical monitors and FHI 360 designated persons to inspect study documents (e.g., informed consent forms, product distribution forms, etc.) and pertinent clinic records for confirmation of the study data. A site visit log will be maintained at the study site in which all site monitoring visits made by authorized individuals are recorded.

A detailed clinical monitoring plan will be developed for this study and will be used by all staff who monitor the study sites. This plan specifies the responsibilities and qualifications of the clinical monitors, back-up provisions and site monitoring visit procedures. All monitoring visits will be documented.

16.0 STEERING COMMITTEE

To ensure ongoing quality, a steering committee comprised of Drs. Todd, Myer, Jones, and the study coordinator will be responsible for oversight. The Steering Committee will meet monthly by teleconference

to review study protocols, progress, staffing, and findings. The Committee will be responsible for allocating tasks to select consultants or collaborators.

17.0 ADMINSTRATIVE

17.1 Protocol Violations

A "protocol violation" is broadly defined as any deviation from the procedures described in the study protocol. Protocol violations exist in a range from extremely minor to those that place the study participants in jeopardy. Protocol violations may be identified by the PI, collaborators, study staff or the clinical monitor. The procedures for capturing, recording and reporting protocol violations will be specified in the monitoring plan.

Emergency departures from protocol that eliminate an apparent immediate hazard to participants and are deemed crucial for the safety and well-being of that participant may be instituted for that participant only by the Site PI or designee. In those cases, the Site PI or designee will notify the IRB and FHI 360 in writing as soon as possible and document on the reasons for the violation and ensuing events.

Reporting of protocol violations (including those by participants) will be described in the monitoring plan.

17.2 Study Records

After study closure, the signed original informed consent documents for each participant, data forms, and originals of all study and source documentation will be retained by the Site PI for a minimum of 3 years after the study report/publication is completed, whichever comes last. No records may be destroyed without prior written permission from FHI 360.

17.3 Publication Policy

In determining authorship for publications, FHI 360's Authorship of Publications Reporting Data from FHI 360 Sponsored Studies (Policy and Procedure No. 40.01) will be followed (available from the FHI 360 Medical Monitor/Project Leader upon request). FHI 360 policy regarding authorship will essentially follow the guidelines of the International Committee of Medical Journal Editors.[80] The main publication will follow the CONSORT guidelines.[81]

17.4 Research at External Sites

All study visits with exception of some initial screening evaluations will be conducted at the GCHC located in Cape Town, South Africa. No patient care activities will occur at institutions inside the United States. FHI360 will conduct overall study coordination, which will include but is not limited to activities in questionnaire development, site training, site progress assessments, and manuscript preparation. B. Landon Myer (South Africa Site PI) will oversee site activities and provide daily clinical back-up to on-site staff. Dr. Myer is an epidemiologist and licensed HIV clinician with the Desmond Tutu HIV Centre. The University of Cape Town will be submitting to their own IRB, which will serve as the local entity responsible for review and approval of the protocol. Monitoring at the study site will be performed semi-annually by Westat Corporation.

18.0 TIMELINE

The following chart depicts the proposed timeline. Cohort recruitment will begin within nine months of study initiation, with completion of recruitment anticipated within a further nine months. Based on these estimations, study activities will be completed by the end of the fourth year. The fifth year will be used for data analysis and preparation of manuscripts, policy recommendations, and any follow-on studies.

**Timeline**<br>Project task	Yr. 1	Yr. 2	Yr. 3	Yr. 4	Yr. 5	Yr. 6
Obtain IRB and other approvals	X X X					
Hire and train study coordinator and interviewers	X X					
Develop, translate, and test study instruments	X X X					
Database Development and Training	X X					
Trial participant pre-screening & recruitment drives	X X					
Trial participant recruitment and enrollment	X	X X XX	XXXX	XXXX	XX	
Participant follow-up visits		X X X X	X X X X	X X X X	XXXX	XX
Routine laboratory testing (urine HCG, HIV-1 VLs, CD4, Hgb, STI		X X X X	X X X X	X X X X	XXXX	X
Discontinuation Visits/ Interviews		X X X X	X X X X	X X X X	XXXX	X
Data entry in South Africa and electronic transfer of data		X X X X	X X X X	X X X X	XXXX	XX
Final Statistical Analysis						XXXX
Dissemination and reporting of results					XX	XXX

X=denotes activity is occurring in specified time period.

*Contingent on DSMB determination.

19.0 REFERENCES

1. Sweat MD, O'Reilly KR, Schmid GP, Denison J, de Zoysa I. Cost-effectiveness of nevirapine to prevent mother-to-child HIV transmission in eight African countries. *AIDS*. 2004;18:1661-71.
2. Reynolds HW, Janowitz B, Homan R, Johnson L. The value of contraception to prevent perinatal HIV transmission. *Sex Transm Dis*. 2006; 33:350-6.
3. Hladik W, Stover J, Esiru G, Harper M, Tappero J. The contribution of family planning towards the prevention of vertical HIV transmission in Uganda. *PLoS One*. 2009; 4:e7691.
4. Lindegren ML, Kennedy CE, Bain-Brickley D, Azman H, Creanga AA, Butler LM, et al. Integration of HIV/AIDS services with maternal, neonatal and child health, nutrition, and family planning services. Cochrane Database Syst Rev 2012;9:CD010119.
5. World Health Organization. Technical statement: Hormonal contraception and HIV. WHO/RHR/12.08. WHO, Geneva, 2012.
6. Stringer EM, Kaseba C, Levy J, Sinkala M, Goldenberg RL, Chi BH, Matongo I, Vermund SH, Mwanahamuntu M, Stringer JS. A randomized trial of the intrauterine contraceptive device vs hormonal contraception in women who are infected with the human immunodeficiency virus. Am J Obstet Gynecol. 2007;197:144.e1-8.
7. Stringer EM, Levy J, Sinkala M, Chi BH, Matongo I, Chintu N, Stringer JS. HIV disease progression by hormonal contraceptive method: secondary analysis of a randomized trial. AIDS. 2009;23:1377-82.
8. Heffron R, Donnell D, Rees H, Celum C, Mugo N, Were E, de Bruyn G, Nakku-Joloba E, Ngure K, Kiarie J, Coombs RW, Baeten JM; Partners in Prevention HSV/HIV Transmission Study Team. Use of hormonal contraceptives and risk of HIV-1 transmission: a prospective cohort study. Lancet Infect Dis. 2012;12(1):19-26.
9. World Health Organization. Consolidated guidelines on the use of antiretroviral drugs for treating and preventing HIV infection: recommendations for a public health approach June 2013. WHO, 2013, Geneva, Switzerland.
10. Low AJ, Konate I, Nagot N, Weiss HA, Kania D, Vickerman P, Segondy M, Mabey D, Pillay D, Meda N, van de Perre P, Mayaud P; Yerelon Study Group. Cervicovaginal HIV-1 shedding in women taking antiretroviral therapy in Burkina Faso: a longitudinal study. J Acquir Immune Defic Syndr. 2014 Feb 1;65(2):237-45

11. Herold BC, Keller MJ, Shi Q, Hoover DR, Carpenter CA, Huber A, Parikh UM, Agnew KJ, Minkoff H, Colie C, Nowicki MJ, D'Souza G, Watts DH, Anastos K. Plasma and mucosal HIV viral loads are associated with genital tract inflammation in HIV-infected women. J Acquir Immune Defic Syndr. 2013 Aug 1;63(4):485-93.
12. Day S, Graham SM, Masese LN, Richardson BA, Kiarie JN, Jaoko W, Mandaliya K, Chohan V, Overbaugh J, Mcclelland RS. A Prospective Cohort Study of the Effect of Depot Medroxyprogesterone Acetate on Detection of Plasma and Cervical HIV-1 in Women Initiating and Continuing Antiretroviral Therapy. J Acquir Immune Defic Syndr. 2014 May 4. [Epub ahead of print]
13. Mitchell C, Balkus JE, Fredricks D, Liu C, McKernan-Mullin J, Frenkel LM, Mwachari C, Luque A, Cohn SE, Cohen CR, Coombs R, Hitti J. Interaction between lactobacilli, bacterial vaginosis-associated bacteria, and HIV Type 1 RNA and DNA Genital shedding in U.S. and Kenyan women. AIDS Res Hum Retroviruses. 2013 Jan;29(1):13-9.
14. Cu-Uvin S, DeLong AK, Venkatesh KK, Hogan JW, Ingersoll J, Kurpewski J, De Pasquale MP, D'Aquila R, Caliendo AM. Genital tract HIV-1 RNA shedding among women with below detectable plasma viral load. AIDS. 2010 Oct 23;24(16):2489-97.
15. Coleman JS, Mwachari C, Balkus J, Sanguli L, Muliro A, Agnew K, Coombs RW, Cohen CR, Hitti J. Effect of the levonorgestrel intrauterine device on genital HIV-1 RNA shedding among HIV-1-infected women not taking antiretroviral therapy in Nairobi, Kenya. J Acquir Immune Defic Syndr. 2013 Jun 1;63(2):245-8.
16. Heikinheimo O, Lehtovirta P, Aho I, Ristola M, Paavonen J. The levonorgestrel-releasing intrauterine system in human immunodeficiency virus-infected women: a 5-year follow-up study. Am J Obstet Gynecol. 2011 Feb;204(2):126.e1-4.
17. Baeten JM, Lavreys L, Sagar M, Kreiss JK, Richardson BA, Chohan B, Panteleeff D, Mandaliya K, Ndinya-Achola JO, Overbaugh J, Farley T, Mwachari C, Cohen C, Chipato T, Jaisamrarn U, Kiriwat O, Duerr A. Effect of contraceptive methods on natural history of HIV: studies from the Mombasa cohort. J Acquir Immune Defic Syndr. 2005; 38 Suppl 1:S18-21.
18. Trunova N, Tsai L, Tung S, Schneider E, Harouse J, Gettie A, Simon V, Blanchard J, Cheng-Mayer C. Progestin-based contraceptive suppresses cellular immune responses in SHIV-infected rhesus macaques. Virology. 2006; 352:169-77.

19. Benki S, Mostad SB, Richardson BA, Mandaliya K, Kreiss JK, Overbaugh J. Cyclic shedding of HIV-1 RNA in cervical secretions during the menstrual cycle. J Infect Dis. 2004; 189:2192-201.
20. Stringer E, Antonsen E. Hormonal contraception and HIV disease progression. Clin Infect Dis. 2008; 47:945-51.
21. Morrison CS, Chen PL, Nankya I, Rinaldi A, Van Der Pol B, Ma YR, Chipato T, Mugerwa R, Dunbar M, Arts E, Salata RA. Hormonal contraceptive use and HIV disease progression among women in Uganda and Zimbabwe. J Acquir Immune Defic Syndr. 2011;57:157-64.
22. Richardson BA, Otieno PA, Mbori-Ngacha D, Overbaugh J, Farquhar C, John-Stewart GC. Hormonal contraception and HIV-1 disease progression among postpartum Kenyan women. AIDS. 2007;21:749-53.
23. Cejtin HE, Jacobson L, Springer G, Watts DH, Levine A, Greenblatt R, Anastos K, Minkoff HL, Massad LS, Schmidt JB. Effect of hormonal contraceptive use on plasma HIV-1-RNA levels among HIV-infected women. AIDS. 2003;17:1702-4.
24. Stringer EM, Giganti M, Carter RJ, El-Sadr W, Abrams EJ, Stringer JSA; for the MTCT-Plus Initiative. Hormonal contraception and HIV disease progression: a multicountry cohort analysis of the MTCT-Plus Initiative. AIDS. 2009;23 Suppl 1:S69-S77
25. Polis CB, Wawer MJ, Kiwanuka N, Laeyendecker O, Kagaayi J, Lutalo T, Nalugoda F, Kigozi G, Serwadda D, Gray RH. Effect of hormonal contraceptive use on HIV progression in female HIV seroconverters in Rakai, Uganda. AIDS. 2010;24:1937-44.
26. Folger SG, Curtis KM, Tepper NK, Gaffield ME, Marchbanks PA. Guidance on medical eligibility criteria for contraceptive use: identification of research gaps. Contraception. 2010;82: 113-18.
27. Curtis KM, Nanda K, Kapp N. Safety of hormonal and intrauterine methods of contraception for women with HIV/AIDS: a systematic review. AIDS. 2009;23 Suppl 1:S55-67.
28. Morrison CS, Nanda K. Hormonal contraception and HIV: an unanswered question. Lancet Infect Dis. 2012;12:2-3.
29. WHO. Policy Implications: Programmatic and research considerations for hormonal contraception for women at risk of HIV and women living with HIV. PHP/RHR/12.09. WHO, 2012.
30. Gordenne V, Wijzen F, Foidart J-M, Marmon T, Creinin MD, Frankenne F. Plasma levonorgestrel levels in women using LNG-20, a new levonorgestrel intrauterine system, and Mirena. Abstract P16 for Reproductive Health 2010. In: Contraception. 2010;82:193.

31. Gordenne V, Wijzen F, Foidart J-M, Marmon T, Creinin MD, Frankenne F. Comparison of LNG-20, a new levonorgestrel intrauterine system, and Mirena for treatment of menorrhagia. Abstract P92 for Reproductive Health 2010. In: *Contraception*. 2010,82: 213.
32. Mirena Extended CCDS Bayer Schering Pharma AG, Berlin, Germany. 7th October 2008. [http://www.mirena.com/en/professional/contraception/pharmacokinetics/index.php].
33. Diaz S, Pavez M, Miranda P, Johansson ED, Croxatto HB. Long-term follow-up of women treated with NORPLANT® implants. Contraception. 1987;35:551–67.
34. Kuhnz W, al-Yacoub G, Fuhrmeister A. Pharmacokinetics of levonorgestrel in 12 women who received a single oral dose of 0.15 mg levonorgestrel and, after a washout phase, the same dose during one treatment cycle. Contraception. 1992; 46:443-454.
35. World Health Organization. Improving access to quality care in family planning: medical eligibility criteria for contraceptive use, 3rd edition. World Health Organization, Geneva, Switzerland, 2004.
36. Nilsson CG, Lahteenmaki PL, Luukkainen T, Robertson DN. Sustained intrauterine release of levonorgestrel over five years. Fertil Steril 1986; 45:805-807.
37. Lehtovirta P, Paavonen J, Heikinheimo O. Experience with the levonorgestrel-releasing intrauterine system among HIV-infected women. Contraception. 2007;75:37-9.
38. Heikinheimo O, Lehtovirta P, Suni J, Paavonen J. The levonorgestrel-releasing intrauterine system (LNG IUD) in HIV-infected women--effects on bleeding patterns, ovarian function and genital shedding of HIV. Hum Reprod. 2006;21:2857-61.
39. Jensen JT. Noncontraceptive applications of the levonorgestrel intrauterine system. Curr Womens Health Rep. 2002;2:417-22.
40. Baumgartner JN, Morroni C, Mlobeli RD, Otterness C, Myer L, Janowitz B, Stanback J, Buga G. Timeliness of contraceptive reinjections in South Africa and its relation to unintentional discontinuation. Int Fam Plan Perspect. 2007;33:66-74.
41. Sinei SK, Morrison CS, Sekadde-Kigondu C, Allen M, Kokonya D. Complications of use of intrauterine devices among HIV-1-infected women. Lancet. 1998;351:1238-41.
42. Wesson J, Olawo A, Bukusi V, Solomon M, Pierre-Louis B, Stanback J, Janowitz B. Reaching providers is not enough to increase IUCD use: a factorial experiment of 'academic detailing' in Kenya. J Biosoc Sci. 2008;40:69-82.

43. Gutin SA, Mlobeli R, Moss M, Buga G, Morroni C. Survey of knowledge, attitudes and practices surrounding the intrauterine device in South Africa. Contraception. 2011;83:145-50.
44. Credé S, Hoke T, Constant D, Green MS, Moodley J, Harries J. Factors impacting knowledge and use of long acting and permanent contraceptive methods by postpartum HIV positive and negative women in Cape Town, South Africa: a cross-sectional study. BMC Public Health. 2012;12:197
45. Stanback J, Shelton JD. Pelvic inflammatory disease attributable to the IUD: modeling risk in West Africa. Contraception. 2008;77:227-9
46. Todd CS, Jones HE, Garber TC, Afnan-Holmes H, Woolgar H, Bekker LG, Myer L. Awareness and Interest in Intrauterine Contraceptive Device Use among HIV-Positive Women in Cape Town, South Africa. Infect Dis Obstet Gynecol. 2012;2012:956145
47. Department of Health, Republic of South Africa. The South African Antiretroviral Treatment Guidelines 2011. Pretoria, South Africa, 2011.
48. Kapp N, Curtis KM. Intrauterine device insertion during the postpartum period: a systematic review. Contraception. 2009;80:327-36.
49. National Department of Health, South Africa; South African National AIDS Council. CLINICAL GUIDELINES: PMTCT (Prevention of Mother-to-Child Transmission). 2010. Department of Health, Republic of South Africa.
50. Stringer JS, McConnell MS, Kiarie J, et al. Effectiveness of non-nucleoside reverse-transcriptase inhibitor-based antiretroviral therapy in women previously exposed to a single intrapartum dose of nevirapine: a multi-country, prospective cohort study. PLoS Med. 2010;7(2):e1000233.
51. Brockmeyer A, Kishen M, Webb A. Experience of IUD/IUS insertions and clinical performance in nulliparous women--a pilot study. Eur J Contracept Reprod Health Care. 2008;13(3):248-54.
52. Bayer LL, Jensen JT, Li H, Nichols MD, Bednarek PH. Adolescent experience with intrauterine device insertion and use: a retrospective cohort study. Contraception. 2012 May 4. [Epub ahead of print]
53. Morrison CS, Murphy L, Kwok C, Weiner DH. Identifying appropriate IUD candidates in areas with high prevalence of sexually transmitted infections. Contraception. 2007 Mar;75(3):185-92.
54. Centers for Disease Control and Prevention, Workowski KA, Berman SM. Sexually transmitted diseases treatment guidelines, 2006. MMWR Recomm Rep. 2006; 55(RR-11):1-94.

55. Department of Health, Republic of South Africa. First Line Comprehensive Management and Control of Sexually Transmitted Infections. Pretoria, South Africa, 2007. Available at: http://www.nicd.ac.za/units/stirc/First_Line_Comprehensive_Management_&_Control_of_Sexually_Transmitted_Infections_STIs.pdf
56. Young T, de Kock A, Jones H, Altini L, Ferguson T, van de Wijgert J. A comparison of two methods of partner notification for sexually transmitted infections in South Africa: patient-delivered partner medication and patient-based partner referral. Int J STD AIDS. 2007;18:338-40.
57. Rose S, Chaudhari A, Peterson CM. Mirena (Levonorgestrel intrauterine system): a successful novel drug delivery option in contraception. Adv Drug Deliv Rev. 2009;61:808-12.
58. Dupont WD, Plummer WD: Power and sample size calculations. A review and computer program. Control Clin Trials 1990, 11(2):116-128.
59. Gurunathan S, Habib RE, Baglyos L, Meric C, Plotkin S, Dodet B, Corey L, Tartaglia J. Use of predictive markers of HIV disease progression in vaccine trials. Vaccine. 2009; 27:1997–2015.
60. Kupka R, Mugusi F, Aboud S, Msamanga GI, Finkelstein JL, Spiegelman D, Fawzi WW. Randomized, double-blind, placebo-controlled trial of selenium supplements among HIV-infected pregnant women in Tanzania: effects on maternal and child outcomes. Am J Clin Nutr. 2008;87:1802-8.
61. Kumwenda JJ, Makanani B, Taulo F, Nkhoma C, Kafulafula G, Li Q, Kumwenda N, Taha TE. Natural history and risk factors associated with early and established HIV type 1 infection among reproductive-age women in Malawi. Clin Infect Dis. 2008;46:1913-20.
62. Fawzi WW, Msamanga GI, Spiegelman D, Wei R, Kapiga S, Villamor E, Mwakagile D, Mugusi F, Hertzmark E, Essex M, Hunter DJ. A randomized trial of multivitamin supplements and HIV disease progression and mortality. N Engl J Med. 2004;351:23-32.
63. Backman T, Huhtala S, Blom T, Luoto R, Rauramo I, Koskenvuo M. Length of use and symptoms associated with premature removal of the levonorgestrel intrauterine system: a nation-wide study of 17,360 users. BJOG. 2000;107:335-9.
64. Daud S, Ewies AA. Levonorgestrel-releasing intrauterine system: why do some women dislike it? Gynecol Endocrinol. 2008;24:686-90.
65. Hidalgo M, Bahamondes L, Perrotti M, Diaz J, Dantas-Monteiro C, Petta C. Bleeding patterns and clinical performance of the levonorgestrel-releasing intrauterine system (Mirena) up to two years. Contraception. 2002;65:129-32.

66. Szklo M, Nieto J. Epidemiologu, Beyond the basics 2nd edition Jones & Bartlett, Boston 2007
67. Pepe MS, Anderson GL. A cautionary note on inference for marginal regression models with longitudinal data and general correlated response data. Comm Stat Simula 1994;23:939-51.
68. Rosenbaum PR, Rubin DB. Estimating the effects caused by treatments. J Am Stat Assoc 1984;79:26-28.
69. Liang KY, Zeger S: Longitudinal data analysis of continuous and discrete responses for pre-post designs. Sankhy.a: The Indian Journal of Statistics, Series B 2000, 62:134–148.
70. Diggle PJ, Heagarty PJ, Ling KY, Zeger SL. Analysis of Longitudinal Data. Oxford Press. New York (2002).
71. Lyles RH, Lyles CM, Taylor DJ. Random Regression Models for Human Immunodeficiency Virus Ribonucleic Data Subject to Informative Drop Out Subject to Left Censoring and Informative Dropout, Applied Statistics 49(3) 2000 485-97.
72. Klein JP, Moeschberger ML. Survival Analysis, Techniques for Censored and Truncated Data. Springer-Verlag, 2nd Edition London (2007).
73. Colwell HH, Mathias SD, Cimms TA, Rothman M, Friedman AJ, Patrick DL. The ORTHO BC-SAT--a satisfaction questionnaire for women using hormonal contraceptives. Qual Life Res. 2006; 15:1621-31.
74. Mathias SD, Colwell HH, Lococo JM, Karvois DL, Pritchard ML, Friedman AJ. ORTHO birth control satisfaction assessment tool: assessing sensitivity to change and predictors of satisfaction. Contraception. 2006; 74:303-8.
75. Belsey EM, Farley TM. The analysis of menstrual bleeding patterns: a review. Contraception. 1988; 38:129-56.
76. Suvisaari J, Lahteenmaki P. Detailed analysis of menstrual bleeding patterns after postmenstrual and postabortal insertion of a copper IUCD or a levonorgestrel-releasing intrauterine system. Contraception. 1996; 54:201-8.
77. Glaser B. Basics of grounded theory analysis. Mill Valley, CA: Sociology Press. 1992.
78. Strauss AL, Corbin J. Grounded theory methodology: An overview. In N.K. Denzin & Y. S. Lincoln (eds.), Handbook of qualitative research (pp. 273-285). Thousand Oaks, CA: Sage Publications. 1994.
79. Miles MB, Huberman AM. Qualitative data analysis: An expanded sourcebook (2nd edition). Thousand Oaks, CA: Sage Publications. 1994.

80. Guidelines on authorship. International Committee of Medical Journal Editors. Br Med J (Clin Res Ed) 1985;291(6497):722.

81. Moher D, Schulz KF, Altman DG. The CONSORT statement: revised recommendations for improving the quality of reports of parallel-group randomised trials. Lancet 2001;357(9263):1191-4.

20.0 APPENDICES

Appendix 1: Schedule of Activities by Study Visit

Activity	Baseline Screen	Enrollment / Insertion	3 M	6 M	12 M	18 M	24 M	Unblinding Visit‡	IUD Continuation Calls	Unscheduled Visits
Questionnaire		X	X	X	X	X	X	X	X‡	X*
Exit Questionnaire							X			X*
Device Insertion		X								X*
STI testing/samples taken†	X		X	X	X	X	X	X*		X*
Syphilis testing	X		X	X	X	X	X			X*
Urine pregnancy testing	X	X	X	X	X	X	X	X		X*
Plasma VL		X	X	X	X	X	X			
CD4 Lymphocyte Count**	X			X	X	X	X			
Full Blood Count		X		X	X	X	X			
Pelvic Exam/ Genital VL Sampling	X (exam only)	X	X	X	X	X	X	X*		X*
Supplemental testing (select residual and ECS samples)		X	X	X	X	X	X			
Device Removal			X*	X*	X*	X*	X*	X*		X*
Unblinding and counseling								X		

*As needed based on participant request/problem

† Testing performed for screening visits; samples taken and frozen for follow-up visits with testing performed as funding available.

‡ For participants not yet eligible for 24 month visit (IUD continuation calls only) or presenting after follow-up period closed (June 8, 2018).

Appendix 2: IUD Insertion Risk Screening Instrument

**2IUDnCT IUD Risk Assessment Screening Tool**			
**#**	**Question**	**Answer**	**Score**
1	Are you less than 25 years old?	0…. No<br>1…. Yes	____
2.	Do you currently live apart from your husband or partner?	0…. No<br>1…. Yes<br>0….No partner in last three months	____
3.	During the last year, have you had bleeding between periods (at least 2 days before or 2 days after your menstrual cycle) and/or bleeding or spotting within 24 hours after sex?	0…. No<br>1…. Yes	____
4.	Have you passed the Matric exam?	0…. No<br>1…. Yes	____
5.	How many (different) sexual partners have you had during the last 3 months?	No partners<br>1 partner<br>>1 partner	Go to 6.<br>Go to 7.<br>Go to 8.
6.	Have you had any sexual partner in the last 3 months.	0…. No<br>Yes (go back to 5)	____<br>Finish and go to scoring
**Please note difference in coding for 1 partner (Q7) vs. more than 1 partner (Q8).**			

7.	With your sexual partner, how often did you use condoms in the last 3 months?	0….One partner, never used condoms<br><br>1….One partner, sometimes used condoms<br><br>0….One partner, always used condoms	___
8.	How often did you use a condom when you had sex with any partner during the last 3 months?	1….More than one partner, never used condoms<br><br>1….More than one partner, sometimes used condoms<br><br>0….More than one partner, always used condoms	___
**SCORING (Maximum is score of 5)**		**Add points in far right column for cumulative score.**	___

Nurse signature: ______________________________ Date:_____________________

Appendix 3: Lab Considerations

The study laboratory plan will include the procedures for specimen management (e.g. chain of custody, handling, labeling and transport), assay procedures, proficiency testing and quality assurance procedures and specimen storage procedures.

Laboratory Specimens

The following types of specimens will be collected for testing:

- Urine for pregnancy testing
- Blood for hematology (full blood count [FBC])
- Blood for HIV viral load testing by PCR assay
- Blood for syphilis testing
- Blood (plasma and serum) for archive
- Genital specimens for gonorrhea and Chlamydia testing
- Genital specimens for HIV viral load testing
- Genital specimens for archive

All the above specimens will be collected with Good Clinical and Laboratory Practice principles and as described in the SOPs for collection of specimens.

On site testing

The study laboratory plan will detail the procedures to be followed for on-site testing as well as proficiency testing for all on-site testing (i.e. urine pregnancy tests and syphilis, bacterial vaginosis, and trichomonas rapid tests).

Collection and shipping of specimens

All specimens (blood, urine, and genital) will be collected according to methods described in the study laboratory plan and SOPs for proper collection, processing, labeling, and transport of specimens to the laboratories conducting the assays or sites storing the specimens. All specimens retained for quality assurance or future testing will be stored at the University of Cape Town.

Specimen Storage for Quality Assurance and Potential Future Research Testing

Serum, plasma, and genital specimens will be stored for potential post-trial assessments for markers of safety and product adherence at the University of Cape Town. Where possible, stored specimens will be re-tested to assess the validity of unusual or unexpected assays results. For those participants who do not consent to long-term storage of their specimens, any residual specimens will be destroyed at the end of the study after all protocol-required and quality assurance testing has been completed.

Laboratory Quality Control and Quality Assurance Procedures

The laboratories involved in the study will follow the quality assurance and quality control procedures outlined in the study laboratory plan. For the on-site tests, the quality assurance personnel from the laboratory will conduct periodic visits to the site to assess the implementation of on-site quality control procedures, including maintenance of laboratory testing equipment, use of appropriate reagents, proficiency testing records and quality checks of on-site testing procedures.
