# Supplementary material for: Safety and continued use of the levonorgestrel intrauterine system as compared with the copper intrauterine device among women living with HIV in South Africa: A randomized controlled trial
Source: PLoS Med. 2020 May 22;17(5):e1003110. doi: 10.1371/journal.pmed.1003110 (PMC7244096; doi:10.1371/journal.pmed.1003110)
Supplement: S2 Text — Statistical Analysis Plan, Version 9.0. (PDF) [file pmed.1003110.s010.pdf]

Comparison of Two IUDs among Cape Town HIV-positive Women: A Randomized Controlled Trial Assessing Safety of Registered Products in South Africa

Statistical Analysis Plan

Version 9.0

September 30, 2016

FHI 360

I. Study Objectives (Primary / Secondary) 3
II. Design Plan 3
III. Analysis Population 3
A. Screened Population 4
B. Intent-to-Treat (ITT) Population 4
C. As Treated (AT) Population / Time 4
IV. Masked Review of the Data 4
V. Analysis of Baseline Data 4
VI. Analysis of Participant Follow-Up 5
A. Analysis Populations 5
B. Presentation of Participant Disposition Characteristics 5
C. Statistical Methodology 5
VII. Analysis of Acceptability and Effectiveness (Safety) Data 6
A. Analysis Population 6
B. Pooling of Data 7
C. Definition of Outcome Variables 7
D. Outcome Variable Transformations 8
E. Covariates or Stratifying variables 8
F. Statistical Methodology 9
1. Primary Efficacy (Safety) Outcome 9
2. Secondary Comparison of HIV Recovery By Genital Sampling Method 9
3. Secondary Acceptability Outcome 10
4. Secondary Comparison of HIV Recovery by Genital Sampling Method 10
5. Other Secondary Safety Outcomes 10
6. Other Secondary Acceptability Outcomes 10
7. Pregnancy Incidence Following IUD Discontinuation 11
8. Other Adverse Events and Concomitant Treatment 11
G. Missing Data 12
H. Study Visit Windows 12
I. Subgroup Analyses 12

IX. References 14

I. Study Objectives

Primary Objective:

To measure levonogestrel intrauterine device (LNG IUD) safety as compared to the copper IUD (C-IUD) with respect to genital HIV RNA shedding, a surrogate for HIV infectivity. The null hypothesis being one-sided non-inferiority that LNG-IUD is no worse than C-IUD.

Secondary Objectives:

1) To compare the safety of the LNG IUD to that of C-IUD in HIV infected women not yet ART-eligible (Pre-ART) at enrollment in terms of impact on HIV progression as indicated by HIV-1 plasma viral load. This is a one-sided noninferiority hypothesis test that that LNG-IUD is no worse than C-IUD.

2) To measure LNG IUD acceptability through device continuation and other measures as compared to the C-IUD. This is a two-sided hypothesis test.

3) To compare the recovery rate of HIV RNA and immune mediators from the female genital tract between three different sampling methods. This involves two-sided hypothesis tests.

II. Design Plan

The main study is a single site, double-blind, and parallel Randomized Clinical Trial (RCT) in Gugulethu, Cape Town, South Africa with 206 HIV-positive women drawn from two groups: 70 women not yet eligible for ART by CD4 count>500 cells/mm^3 at screening (Pre-ART) and 136 women using ART at screening. Both Pre-ART and ART-using participants will be randomized in a 1:1 ratio to LNG IUD and C-IUD arms and followed at 3, 6, 12, 18, and 24 months to determine comparative safety in terms of HIV transmission potential and progression, as well as comparative acceptability. The randomization will be blocked and stratified on ART use (yes vs. no), age (18-23, 24-30, 31-40), and depot medroxyprogesterone acetate (DMPA)/norethesterone enanthate (Net-En) use (yes vs. no) within 4 months prior to study entry. Both Treatment Arms will receive individual risk-reduction counseling, sexually transmitted infection (STI) screening, treatment for any diagnosed curable STI, and condoms free of charge. In a nested substudy to compare HIV RNA recovery using different methods of genital sampling, endocervical swab (ECS), enriched cervicovaginal lavage (eCVL), and menstrual cup (MC) vaginal samples each will be taken at baseline, then at the 3, and 6 month follow up visits with each of the three samples being quantified for genital HIV VL.

III. Analysis Populations

A. Screened Population

This group will consist of all women screened for study participation and properly consented both for the screening visit and, if eligible, for trial entry. From this screened population, a total of 206 HIV-positive South African women from Gugulethu, Cape Town, South Africa and between the ages of 18 and 40 years inclusive will be enrolled from public sector clinics around Gugulethu and Nyanga. Descriptive analyses will be performed to compare those enrolled (with the IUD inserted) versus those eligible, but declining enrollment or those ineligible using exact or trend tests for categorical characteristics and t-tests or rank tests for continuous characteristics.

B. Intent-to-Treat (ITT) Population

This group will consist of all participants who are properly consented, enrolled, and randomized to treatment and have the IUD inserted. Women who are consented or randomized but do not have the IUD inserted are censored from analysis. ITT comparisons will be made between the randomly assigned treatment arms. In particular, for analysis of acceptability of the IUD devices, all women will be included and analyzed consistent with intent to treat.

C. As Treated (AT) Population / Time

In this grouping, participants will contribute analysis time only during time of IUD (either C-IUD or LNG IUD) utilization and, for impact of IUD type on genital viral shedding and plasma VL, and for those initially recruited not using ART only while they remain not using ART. While the AT population consists of all (ITT) participants as they will all have the IUD device inserted, it excludes any time period in these participants that occurs after the device has been removed or, for impact of IUD type on genital viral shedding and plasma VL, and for those recruited not using ART, any time after ART had been initiated. The safety analyses will be performed on the AT cohort-time.

IV. Masked Review of Data

All personnel involved in determination of inclusion in or exclusion from the various subsets of the ITT or AT population will be fully masked to the treatment assignment of the participants. Any key decisions regarding the timing of outcomes, the appropriateness of test statistics or model assumptions, changes to this analysis plan, or any other statistical issues will be made under such masked review of the data. One analyst will be partially unmasked to treatment groups reported as A vs. B (with any specific treatment assigned to a particular arm not specified) during the final analysis. While annual DSMB reports will report aggregate data for AEs, SAEs, and protocol deviations, this analyst will be available to provide these data by group A or B should the Data Safety and Monitoring Board (DSMB) request them. These results will be reported to the Study Coordinator who will know the true identity of A and B and also be available to the DSMB Chair for disclosure to inform discussion during closed DSMB meetings for the safety outcomes. Other members of the team will be fully masked to the identity of the treatment groups. This masking will be maintained until the statistical conclusions regarding the relative safety of the masked treatment groups have been obtained and verified. Unmasking of all study personnel with respect to the true treatment groups will only be done for the final

interpretation of the results or as directed by the DSMB. The dates of partial unmasking and complete unmasking will be documented in the final study report.

V. Analysis of Baseline Data

Baseline data will be summarized for all those who were; i) Screened, ii) Randomized, iii) Nonrandomized, iv) Enrolled and v) Included in each Treatment Arm Group. Descriptive analysis of characteristics of all recruits will be made using means (±standard deviations), medians (with interquartile ranges), and proportions as appropriate to ascertain representativeness of the target population and success of the recruitment plan. Comparisons between study arm assignment (LNG-IUD vs. C-IUD) at enrollment will be made using t-tests, rank tests or exact tests, as appropriate to ascertain if in spite of randomization, there are qualitative differences between the study arms. These comparisons will be also be stratified by ART use at enrollment. In particular, the treatment arms will be compared (both overall and stratified by ART use at baseline) by baseline age, CD4 counts, plasma HIV VLs, presence of genital tract HIV RNA shedding, prior contraception use history (DMPA/Net-En exposure within 4 months of study entry), civil status, education, number of prior pregnancies, sexually transmitted infection (STI) history, screening STI status, and other relevant medical and demographic characteristics collected at the screening and enrollment visits.

VI. Analysis of Participant Follow-up or Participant Disposition

A. Analysis Populations

Participant disposition will be summarized for the i) Screened, ii) ITT, iii) AT, iv) subsequent ART initiation (among those initially ineligible for ART) and v) dropout "populations" over time. The ITT and AT populations will be summarized for the total study, as well as separately by ART use at baseline.

B. Presentation of Participant Disposition Characteristics

The number of screened and randomized participants and number of post-baseline ART initiators (from Pre-ART arms), number of study visits completed, number of person-years completed and participant end of study status (including completed study not initiating ART for those not using ART at entry, lost to follow-up, or discontinued IUD or participation before study completion) will be summarized over study time by treatment group (both overall and stratified by ART use at enrollment). Crude incidence rates of IUD/study discontinuation and loss to follow-up and estimates of cumulative IUD/study discontinuation, ART initiation (for Pre-ART group), and loss to follow-up probabilities at each study interval (e.g., 3, 6, 12, 18, and 24 months) will be provided with 95% confidence intervals by treatment group (both overall and stratified by ART use at enrollment). Listings of participant disposition will include masked treatment assignment, age, other relevant baseline and study factors (e.g., baseline / interval plasma VL), and final study status. A diagram showing the flow of participants through the trial and the relationship of the Screened, ITT, and AT populations will be provided.

As the study progresses, the portions of randomized participants that; i) remained in the study in the AT population and still using the IUD (AT population), ii) have remained in the study but have discontinued using the IUD, iii) (for the Pre-ART group) have initiated ART and iv) have dropped out of the study at 3, 6, 12, 18, and 24 months will be calculated both overall and by study arm (both overall and when appropriate stratified by ART use at enrollment) using competing risk survival models or life table based equivalent approaches.[1,2] This will include comparisons to losses at each stage that we had projected and statistical comparisons by study arm for composition of i), ii), iii) and iv) above.

C. Statistical Methodology

Comparability of Treatment Arms at end of study status will be evaluated using Mantel-Haenszel tests both overall, and stratified by; ART use at enrollment, age group (18-23, 24-30, 31-40 years) and DMPA/Net-En exposure within 4 months prior to study entry. Unadjusted Treatment Arm similarity in IUD/study discontinuation, ART initiation (among the Pre-ART group), and loss to follow-up rates will be statistically assessed with log rank tests. Cumulative IUD/study discontinuation probabilities and their confidence intervals will be based on the Kaplan-Meier method or competing risk analogs and Greenwood's formula for standard errors.[1] The population for these analyses will be the ITT Population. Among the Pre-ART group, cumulative ART initiation for the ITT population will be calculated and compared using competing risks (with IUD discontinuations as the competing risk) methods.[1] All tests noted for this section (Section VI) will be conducted at a two-sided $\alpha=0.05$ significance level.

VII. Analysis of Safety and Acceptability Study Data

The primary study outcome will be potential HIV transmission safety, using genital tract HIV shedding measured through VL as a binary yes/no outcome measured at 6 months in the AT population. Initiation of ART per current South African Guidelines may result in ART being used after the 6 month visit; changes in ART initiation criteria during the course of the study will result in variable inclusion of women with enrollment CD4 lymphocyte counts between 350 and 500 cells/mm^3 inclusive. Subsequent ART use among Pre-ART participants should drastically reduce HIV plasma VLs and thus genital tract HIV RNA shedding; however, genital tract HIV RNA shedding remains present in many women even when plasma VL is suppressed with ART use.[3-5] We believe that detectable genital tract HIV RNA response should occur by 6 months, based on response of plasma HIV VL to herpes simplex 2 virus suppressive therapy within 8 to 12 weeks by ART-naïve populations.[6-10] Therefore, a comparison of 6 month plasma VL and viral shedding measures should capture any effect from the LNG IUD without being influenced by ART initiation CD4 threshold biases.

This and other IUD safety outcomes will be tested at a one-sided $\alpha = 0.05$ for the LNG IUD to be inferior to the C-IUD arm. The secondary study outcomes will be: i) for plasma VL measured values at the 6 month visit in the AT population among Pre-ART participants as a one-sided noninferiority hypothesis and ii) IUD acceptability (i.e. continuation of using the device at 24 months as a binary and censored binary outcome) in the ITT population. The IUD device acceptability outcome will be tested at a two-sided $\alpha=0.05$ and the IUD safety outcomes at a one-sided $\alpha=0.05$. Repeated measures analyses of all study genital tract HIV RNA values

measures will also be employed as described in the next paragraph as secondary analyses of the potential HIV safety effect of the LNG IUD on these outcomes, with a one-sided $\alpha=0.05$.[1]

The full range of time over the 24 months of follow-up is available for evaluation of IUD acceptability and safety. As previously noted, for the Pre-ART group assessment of change in HIV RNA shedding, ART initiation will reduce HIV plasma VLs. Therefore, for Pre-ART participants, if ART initiation (based on CD4 count decline) happens more frequently in one study arm, because that arm is more rapidly progressing or by chance due to timing of changes in ART initiation clinical practice guidelines, then the effect of having more post-ART initiation measures in this arm will (in naïve analyses) make that arm appear to be protective, not harmful. While more sophisticated analyses that use ART initiation at a CD4 threshold (or censoring at ART initiation by falling below a CD4 threshold) can and will be fit to account for such biases,[11, 12] these are all to some degree assumption dependent. This will be exploratory and not used to make primary conclusions.

A. Analysis Population – Identify the primary population that will be used for these analyses. Define the circumstances under which analyses will be repeated with a different population

1. For IUD HIV related safety analyses (genital tract HIV viral shedding, the primary outcome of this study, and HIV Plasma VL), an AT approach will be used with those women who remain "as treated" still using the randomized devices at 6 months as the primary outcome. This will exclude any Pre-ART women that subsequently initiate ART prior to 6 months, which is unlikely due to the 6 month interval between CD4 measures, consistent with monitoring advised in clinical guidelines. As described elsewhere in the plan, repeated measure analyses over the full 24 month follow-up period of all randomized women with HIV VL measures from all pre device discontinuation time points will be included in longitudinal analysis for genital tract HIV shedding. These analyses will be run for the total population as well as stratified by ART use at baseline.

2. For plasma VL change, the AT population will be limited to women who are Pre-ART at enrollment until the point of ART initiation, a censoring event. This will exclude any Pre-ART women that subsequently initiate ART prior to 6 months, which is unlikely due to the 6-month interval between CD4 measures, consistent with monitoring advised in clinical guidelines. For IUD acceptability outcomes, all women randomized to each arm (ITT) will be the analysis population with the outcome of interest being continued use of the IUD that they were randomized to receive.

B. Pooling of Data – Describe how summary data will be presented e.g., by district, hospital, gender and/or pooled

For the vast majority of analyses, all data will be pooled together as there is only one gender and catchment area. As noted elsewhere in the proposal, we will undertake stratified analyses by important characteristics (i.e. age groups, Pre-ART vs. ART using at enrollment, and pre-study DMPA/Net-En exposure) and, if the results indicate strong differential main effects and/or

particular interactions with the study outcomes, then stratified or subgroup analyses on these characteristics will be done.

C. Definitions of Outcome Variables

The following outcomes are most critical to the analysis:

- Detectable qualitative genital VL (yes, no) as a binary variable is the primary HIV progression safety outcome. While this variable is dichotomous, a portion is expected to be undetectable with additional skewness at the upper end of the range. This will also be analyzed as quantitative genital tract VL with $\log_{10}$ values used in analysis.
- Plasma HIV VL as a continuous variable is a secondary HIV progression safety outcome. This will be transformed to $\log_{10}$. Before $\log_{10}$ transformation, those with undetectable levels will be assigned the midpoint between 0 and the lower limit of detection for the test.
- Time to discontinuation of the IUD device as a binary outcome with right censoring (survival analysis) as the secondary outcome of acceptability.
- For Pre-ART participants, CD4 count as a continuous variable, both as an important study mediator and potentially as a secondary HIV progression safety outcome. Due to the fact that initiation of ART now occurs at a CD4 threshold of $\leq$ 500 cells/mm^3 censors women from the analysis of plasma VL change, we believe that this outcome will not be skewed or otherwise need transformation.
- Hemoglobin as a continuous variable as a secondary safety outcome.

D. Outcome Variable Transformations

When taking the logarithm of plasma VL, those below the lower limit of detection (currently 40 copies) will be assigned half of that limit (i.e., 20). As genital viral shedding is expected to be a very skewed variable with a point mass mode at 0 (i.e. undetectable), it is anticipated that this will be analyzed mostly as a binary variable (detectable vs. undetectable). However, a two stage approach will also be considered where the treatment arms are first compared with respect to detectable vs. undetectable viral shedding and then, among those with detectable shedding, the level of $\log_{10}$ transformed HIV VL in the genital compartment will be compared between the treatment arms. It is not anticipated otherwise that continuous variables will be categorized in particular for $\log_{10}$ plasma HIV VL, in pre-ART subjects as few (<10%) are expected to be undetectable. Continuous predictor variables with highly skewed distributions may be categorized according to existing conventions or otherwise to form ordered quartiles or tertiles of the analyzed study population.

E. Covariates or Stratifying Variables

This is a RCT (stratified randomized by ART use at enrollment, age group (18-23, 24-30, 31-40) and DMPA/Net-En usage in the four months prior to study entry) so the two study arms should be balanced with respect to potential confounders, particularly for ART use at enrollment, age, and pre-study recent DMPA/Net-En exposure. To increase power by controlling for regression to the mean, baseline levels of genital and plasma HIV $\log_{10}$ VL will always be adjusted for in

analyses, respectively, for post intervention change in these outcomes either as a covariate in Analysis of Covariance (ANCOVA) linear (for baseline log_{10} VL when predicting post-intervention log_{10} VL) or logistic regression models (using detectable baseline genital VL when predicting detectable post-intervention genital VL), or as a linked outcome in mixed or generalized estimation equation models.[2,13] For acceptance of the LNG IUD vs. the C-IUD as an outcome, the main comparisons will be unadjusted exact tests or discrete Kaplan-Meier analysis of time to discontinuation as described later. Other covariates that may be related to outcomes such as parity, partnership status, concomitant medication use, STIs at baseline, and/or reported vaginal practices will be included either as predictors or as stratification variables in exploratory sensitivity analyses of linear, logistic or proportional hazards models.[1,2]

F. Statistical Methodology

1. The Primary Outcome of HIV RNA Genital Shedding, The primary safety outcome, genital tract HIV shedding, will be measured by comparative change in genital tract VL detection from baseline to 6 months for women that use the LNG IUD vs. C-IUD over this time period. HIV RNA genital shedding, a surrogate for HIV infectivity at 6 months, will be measured as a binary outcome (Detectable, Undetectable). This will be compared separately for pre-ART and ART-using women with Mantel-Hanszel stratified odds ratios of [IUD Arm Usage x genital HIV virus detection], stratified in each group by any detectable genital VL (vs. undetectable) at baseline.[14] We will also combine these comparisons between ART and pre-ART participants by pooling all observations together in a Mantel-Haenszel odds ratio that stratifies by both baseline genital VL detection and ART use. Additional analyses of repeated post-baseline measures with detectable genital VL will use GEE with a logit link function, including baseline genital VL detection, current ART use, and treatment arm assignment as predictor covariates and an independent working correlation structure.[15] Time to first detected genital shedding will also be compared between study arms by Cox proportional hazards models (including other covariates such as time dependent ART use). If many specimens show detectable genital VL levels, then two stage models will be considered assessing shedding presence and quantity (on the log_{10} scale) of genital VL. The first stage will compare probability of having detectable genital VL between the treatment arms as a binary outcome (exact tests, logistic regression). The second stage will compare mean value of log_{10} genital VL among those with detectable levels as a continuous outcome (linear models). These models will be adjusted for presence of viral shedding prior to IUD insertion as a stratifying covariate.

2. The Secondary safety outcome*,* For the secondary safety outcome of plasma HIV VL in Pre-ART participants, HIV progression will be measured by comparative change in log_{10} plasma VL from baseline to 6 months for Pre-ART participants that use the LNG IUD vs. C-IUD over this time period. For an “enrollment value only adjusted analysis”, we will use an ANCOVA approach or equivalently constrained longitudinal data analysis (CLDA) that effectively incorporates the enrollment (pre intervention delivery) level of plasma log_{10} VL and treatment arm assignment as predictors into a model with the 6 month VL as the outcome.[2,13] Expressed mathematically in the ANCOVA form:

$$Y_i = a + bX_i + cT + e_i \qquad (*)$$

Where i denotes the study participant, Y is the $\log_{10}$ VL measure at 6 months, X is the $\log_{10}$ VL measure at enrollment, T is the treatment arm assignment (0 = C-IUD, 1 = LNG IUD) and e is independent normal error while a, b and c are constants. The null hypothesis is that c = 0 or that once baseline VL is adjusted for, there is no association between treatment arm assignment and 6 month VL measures. To confirm the enrollment only value adjusted analysis, the comparisons in (*) will then also be adjusted by age and hormonal contraceptive exposure at baseline, which are stratification factors at enrollment, and other patient characteristics that are related to the outcome by adding a vector of these covariates Z with a constant parameter vector d to the right-hand side of (*). Stepwise selection models with treatment arm age group and hormonal contraceptive usage forced in and (two-sided) p-value to enter and stay of 0.10 for other variables will be used to obtain the final adjusted model.

In sensitivity analyses that focus on longer term plasma VL outcomes, repeat measure VL load outcomes across all study visits will be compared by arm using $\log_{10}$ VL measures from all time-points out to 24 months with mixed models or generalized estimating equations (GEE). In ANCOVA formulation for these analyses, model (*) expands to

$$Y_{ij} = a + bX_i + cT + dZ_i + e_{ij} \qquad (**)$$

where j denotes time of VL measure (3, 6, ….., 24 months), the covariate vector Z now includes indicators of which monthly follow up visit the measure is from, but otherwise is time invariant and within the same person i, error e_{ij} is allowed to be correlated. Stepwise selection models with treatment arm forced in and p-value to enter and stay of 0.10 for other variables will be used to obtain the final adjusted model. If other time dependent covariates are included into adjustment, GEE models will conservatively use a diagonal working covariance to prevent estimation bias from within person correlated predictor variables.[15] Otherwise, models that allow for informative censoring by reaching CD4 ART initiation thresholds will be fit as for this and all other efficacy outcome analyses.[11. 12] No VL measurements taken after a woman initiates ART will be included in these analyses. While the LNG hormonal effect "c" will initially be fit as time invariant, if descriptive analysis suggests a time treatment interaction, we will modify (**) to allow for this. For other safety outcomes, we will use Cox Proportional Hazards (PH) models to compare incidence rate of STI and PID infections between the two arms. We will use mixed models or GEE as described above to compare repeat longitudinal CD4 and hemoglobin measures between arms.

3. The Secondary acceptability outcome, IUD device acceptability, will be measured by proportional differences in IUD continuation at 24 months between study arms first using exact tests and then adjusted for other covariates using logistic regression. We will also compare time to discontinuation first using Kaplan-Meier curves with log rank tests and then using Cox proportional hazards models for adjusted comparisons, particularly if there is high study dropout before the 24 month end of follow-up.[1] In these models for women who discontinue the IUD, the time of event will be [date of IUD discontinuation – the date of insertion] and the censor status will be 1=Yes. For women who are lost to follow up (or die) before 24 months, the time of event will be [last date known to be using the IUD – date of entry] and the event status will be

0=no. For all other women the time of event will be [date of 24 months visit – date of insertion] and event status will be 0=no. Proportional hazards models will also be fit to adjust these comparisons for other baseline patient characteristics that could potentially influence discontinuation. Several prognostic baseline variables will be used in these adjusted models, such as age, partnership status, pre-study entry injectable contraceptive and ART use, and fertility history. Stepwise selection models with treatment arm forced in and (two-sided) p-value to enter and stay of 0.10 for other variables will be used to obtain the final adjusted model. Change in partnership status, mean bleeding days or a coded trimester (120 days) of amenorrhea, method satisfaction score, and incident ART use may also be included as time-varying covariates in proportional hazard models to identify any mediating relationship of LNG IUD with device discontinuation should a main effect of IUD type be found.

4. Secondary Comparison of HIV Recovery by Genital Sampling Method: Matched statistical methods will compare outcomes from endocervical swab (ECS) to swab-enriched cervicovaginal lavage (eCVL) sampling, ECS to Menstrual Cup (MC) sampling, and MC to eCVL sampling within the same person and across participants for each of the three time points. For these matched within person dichotomous outcomes (detectable, undetectable), statistical models will include McNemar's test of discordant pairs and conditional logistic regression models to adjust for potential covariates for analyses restricted to a single visit per person (i.e., only baseline). For repeated measure analyses of dichotomous outcomes over time, both mixed effects (GLIMMIX) and generalized estimating equations with robust covariance models will be used to adjust standard errors for within-person correlations. For continuous outcomes, such as total $\log_{10}$ genital VL, paired t-tests or Wilcoxon rank sum tests, linear models of differences, and repeated measures linear models with participant as a random effect will be used. All results from months 3 and 6 will further be stratified by presence of STIs and/or bacterial vaginosis (BV) and by LNG IUD exposure to assess whether inflammatory or hormonal conditions impact performance of sampling method. To evaluate within-person correlation of the different sampling methods, Pearson's and Spearman's correlations will be estimated within each sampling visit for continuous outcomes and kappa coefficients for binary or ranked outcomes.

5. Other Secondary Safety Outcomes Analysis:. We will compare pre-ART changes in CD4 between treatment arms. However, for Pre-ART participants, due to censoring by ART initiation at CD4 below 500/uL, we do not anticipate such comparisons to be informative. We will use mixed models (i.e. as described in Section F.2) including GEE to compare repeat longitudinal CD4 and hemoglobin measures between arms.

6. Other Secondary Acceptability Outcome Analysis: Method satisfaction BC-SAT scores will be descriptively compared between arms using means, medians, and standard deviations. Rank tests, t-tests, and linear models will evaluate unadjusted and adjusted associations between study arms for cross sectional levels and changes in these measures. Trends in scores over time will be described with analysis of covariance (ANCOVA) and other mixed model (or GEE) approaches as needed similar to equations (*) and (**). Covariates included in adjusted models will include hemoglobin, parity, partnership status, ART usage, and age. In prior studies, BC-SAT scores which we are able to reliably measure have been close to

normally distributed but if non-normal distribution is detected, transformations of this variable will be considered.[16, 17] Cross sectional levels and changes in bleeding days are included through two measures in this study: hemoglobin and the bleeding sub-section of the BC-SAT instrument. But more detailed bleeding diaries were not being kept reliably by participants and we believe including any data from this measure would have resulted in substantial bias; thus, protocol Version 6.0 has removed use of bleeding diaries. As described earlier, trends in hemoglobin and BC-SAT bleeding scores will be similarly quantified with repeated measures ANCOVA mixed or GEE models and compared to self-reported bleeding days or reported amenorrhea using the same procedures described for satisfaction.

7. Pregnancy incidence following IUD discontinuation will be computed for each treatment arm by life table methods using monthly intervals and corresponding 95% confidence intervals (using Greenwood's method for standard errors) will be calculated by treatment group.[1] No hypothesis tests regarding the pregnancy outcome are planned.

8. Other Adverse Events and Concomitant Contraceptive Medications Other adverse events (AE's) occurring during the study will be summarized in frequency tables (including both the number of each type of adverse event and the number of distinct participants with each type of adverse event), by body system and by treatment group. Listings of all AE's will include information on duration, outcome, severity, and any relation to IUD use. Approximately quarterly, AEs will be coded in a collaborative effort by the study clinician and data management staff. AEs will then be summarized by body system, pooled over treatment arms. Incidence and prevalence rates, pooled over treatment arms, will be calculated for certain important or interesting AEs or groups of AEs (to be determined in collaboration with project leader and study clinician). These summaries will be used for monitoring purposes only, and unless requested will not be included in any interim or final reports.

G. Missing Data

No imputation is planned other than replacing non-detectable HIV plasma viral load with half the detection limit. If longitudinal data is missing through informative dropout (for example, due to persons with CD4 $\leq$ 500/uL who initiate ART and thus are at severely reduced risk for elevated plasma VL levels), then, as described earlier, random regression models that adjust for informative censoring will be fit.[11, 12]

H. Study Visit Windows

Follow-up visits are scheduled for 3, 6, 12, 18 and 24 months. The visit windows will be per the following table:

Visit	Target Date	Window Period	How calculated
3-month visit	3 months post-enrollment	1.5 – 4.5 months post-enrollment	Calculated from date of enrollment
6-month visit	6 months post-enrollment	4.5 – 9 months post-enrollment	Calculated from date of enrollment

12-month visit	12 months post-enrollment	9 – 15 months post-enrollment	Calculated from date of enrollment
18-month visit	18 months post-enrollment	15 – 21 months post-enrollment	Calculated from date of enrollment
24-month visit	24 months post-enrollment	21 months post-enrollment to study completion	Calculated from date of enrollment

Should any dates fall outside the window, they will most likely occur after rather than before the scheduled visit date. Values collected outside the windows may be included in analysis along with an indicator variable that the value was after (or before) the window period.

I. Subgroup Analyses

Except for possible differential LNG effect on HIV plasma load in ART naïve (vs. ART using) participants as ART suppresses plasma HIV viral load, no subgroup analyses are proposed for this study. However, we cannot rule out the possibility that, should initial analyses indicate unanticipated differential effects in certain subgroups (for example, age group (18-23, 24-30, 31-40), or exposure to DMPA/Net-En within four months of study entry (yes, no)), follow-up analyses restricted to those subgroups will be done. Most likely such analyses would be done by restricting the population to the subgroup in question and/or including an interaction term that involves the subgroup.

IX. References

1. Klein JP, Moeschberger ML. Survival Analysis, Techniques for Censored and Truncated Data. Springer-Verlag, 2nd Edition London (2007).

2. Diggle PJ, Heagarty PJ, Ling KY, Zeger SL. Analysis of Longitudinal Data. Oxford Press. New York (2002).

3. Low AJ, Konate I, Nagot N, Weiss HA, Kania D, Vickerman P, Segondy M, Mabey D, Pillay D, Meda N, van de Perre P, Mayaud P; Yerelon Study Group. Cervicovaginal HIV-1 shedding in women taking antiretroviral therapy in Burkina Faso: a longitudinal study. J Acquir Immune Defic Syndr. 2014;65(2):237-45

4. Herold BC, Keller MJ, Shi Q, Hoover DR, Carpenter CA, Huber A, Parikh UM, Agnew KJ, Minkoff H, Colie C, Nowicki MJ, D'Souza G, Watts DH, Anastos K. Plasma and mucosal HIV viral loads are associated with genital tract inflammation in HIV-infected women. J Acquir Immune Defic Syndr. 2013;63(4):485-93.

5. Day S, Graham SM, Masese LN, Richardson BA, Kiarie JN, Jaoko W, Mandaliya K, Chohan V, Overbaugh J, Mcclelland RS. A Prospective Cohort Study of the Effect of Depot Medroxyprogesterone Acetate on Detection of Plasma and Cervical HIV-1 in Women Initiating and Continuing Antiretroviral Therapy. J Acquir Immune Defic Syndr. 2014; 66(4):452-6.

6. Mitchell C, Balkus JE, Fredricks D, Liu C, McKernan-Mullin J, Frenkel LM, Mwachari C, Luque A, Cohn SE, Cohen CR, Coombs R, Hitti J. Interaction between lactobacilli, bacterial vaginosis-associated bacteria, and HIV Type 1 RNA and DNA Genital shedding in U.S. and Kenyan women. AIDS Res Hum Retroviruses. 2013;29(1):13-9.

7. Cu-Uvin S, DeLong AK, Venkatesh KK, Hogan JW, Ingersoll J, Kurpewski J, De Pasquale MP, D'Aquila R, Caliendo AM. Genital tract HIV-1 RNA shedding among women with below detectable plasma viral load. AIDS. 2010;24(16):2489-97.

8. Nagot N, Ouedraogo A, Foulongne V, Konate I, Weiss HA, Vergne L, Defer M-C, Djagbare D, Sanon A, Andonaba J-B. et al. Reduction of HIV-1 RNA levels with therapy to suppress herpes simplex virus.[see comment] *New England Journal of Medicine*. 2007;356(8):790–799. doi: 10.1056/NEJMoa062607.

9. Dunne EF, Whitehead S, Sternberg M, Thepamnuay S, Leelawiwat W, McNicholl JM, Sumanapun S, Tappero JW, Siriprapasiri T, Markowitz L. Suppressive acyclovir therapy reduces HIV cervicovaginal shedding in HIV- and HSV-2-infected women, Chiang Rai, Thailand. *J Acquir Immune Defic Syndr*. 2008;49(1):77–83. doi: 10.1097/QAI.0b013e3181831832.

10. Zuckerman RA, Lucchetti A, Whittington WLH, Sanchez J, Coombs RW, Zuniga R, Magaret AS, Wald A, Corey L, Celum C. Herpes simplex virus (HSV) suppression with valacyclovir reduces rectal and blood plasma HIV-1 levels in HIV-1/HSV-2-seropositive men: a randomized, double-blind, placebo-controlled crossover trial. *Journal of Infectious Diseases.* 2007;196(10):1500–1508. doi: 10.1086/522523.

11. Schluchter MD (1992) Methods for the analysis of informatively censored longitudinal data. *Stad Med* 11; 1861-70

12. Lyles RH, Lyles CM, Taylor DJ. Random Regression Models for Human Immunodeficiency Virus Ribonucleic Data Subject to Informative Drop Out Subject to Left Censoring and Informative Dropout, *Applied Statistics* 49(3) 2000 485-97.

13. Liang KY, Zeger S. Longitudinal data analysis of continuous and discrete responses for pre-post designs. *Sankhy.a*: *The Indian Journal of Statistics, Series B* 2000; **62**:134–148.

14. Szklo M, Nieto J. Epidemiologu, Beyond the basics 2nd edition Jones & Bartlett, Boston 2007

15. Pepe MS, Anderson GL. A cautionary note on inference for marginal regression models with longitudinal data and general correlated response data. *Comm Stat Simula* 1994;23:939-51.

16. Colwell HH, Mathias SD, Cimms TA, Rothman M, Friedman AJ, Patrick DL. The ORTHO BC-SAT--a satisfaction questionnaire for women using hormonal contraceptives. *Qual Life Res.* 2006; 15:1621-31.

17. Mathias SD, Colwell HH, Lococo JM, Karvois DL, Pritchard ML, Friedman AJ. ORTHO birth control satisfaction assessment tool: assessing sensitivity to change and predictors of satisfaction. *Contraception.* 2006; 74:303-8.

18. Friedman, Lawrence M., Furberg, Curt D., DeMets, David L. Fundamentals of Clinical Trials 4th ed. 2010, Springer-Verlag New York,
