# Supplementary material for: Safety and continued use of the levonorgestrel intrauterine system as compared with the copper intrauterine device among women living with HIV in South Africa: A randomized controlled trial
Source: PLoS Med. 2020 May 22;17(5):e1003110. doi: 10.1371/journal.pmed.1003110 (PMC7244096; doi:10.1371/journal.pmed.1003110)
Supplement: Attachment — Submitted filename: ResponseLetterTodd_2IUDnCT_26MAR20.pdf [file pmed.1003110.s012.pdf]

fhi360
THE SCIENCE OF IMPROVING LIVES

March 26, 2020

Richard Turner, PhD, Senior Editor
PLOS Medicine Special Issue: Maternal and Child Health & Nutrition
1160 Battery Street, Suite 225, San Francisco, CA 94111
plosone@plos.org

RE: Second revision of “Comparison of safety and continuation of the levonorgestrel intrauterine system versus the copper intrauterine device among women living with HIV in South Africa: a randomized controlled trial”

Dear Dr. Turner:

Thank you and the reviewer for this last round of comments. Please find below our responses as well as the revised manuscript and submission information.

Requests from Editors:
Please finalize the arrangements for data deposition.

The de-identified database and variable specifications have been deposited on the Harvard Dataverse and are available at this link:
https://dataverse.harvard.edu/dataset.xhtml?persistentId=doi:10.7910/DVN/NTN7KY
This information is provided within the metadata.

As a general observation, we suggest mentioning the non-inferiority design (line 154) in the abstract. In places (e.g. line 65, "did not increase") the wording might suggest to some readers that a superiority trial was done, and we suggest some judicious rewording (e.g., "there was no significant difference").

We have adjusted the wording as suggested in the abstract in lines 32 for non-inferiority trial. All comparisons in the abstract have been changed from “did not differ” to “there was no significant difference” in lines 48, 51, 53, 54, and 56.

On this same point, around line 250 the wording seems to imply that your aim was to "detect a difference", and we suggest amending this to "... gave 80% power to exclude a difference of 27.7% or more", for example. Are you able to state a non-inferiority margin for the primary endpoint assessment in your methods section?

fhi360
THE SCIENCE OF IMPROVING LIVES

We have changed the wording to that suggested for describing power to detect a difference in lines 260-262. However, the study sample size calculation was not based on the non-inferiority test so we are not able to state a margin for the primary endpoint.

We suggest amending the title to "Safety and continued use of the levonorgestrel intrauterine system as compared with the copper intrauterine device among women living with HIV in South Africa: a randomized controlled trial" or similar.

The title has been changed to the one suggested.

Please trim the discussion of limitations in your abstract; for example, the comment about change of assay could be compressed to "... and a change in the assay used for viral load testing during the study" or similar.

We have reduced the limitations section wording in lines 67 – 71: "Limitations included not achieving non-ART group sample size following change in ART treatment guidelines and truncated 24 months outcome data as 17 women were not yet eligible for their 24-month visit at study closure. Also, a change in VL assay during the study may have caused some discrepancy in VL values due to different limits of detection."

Where you make claims such as "the first" (e.g., at line 98) please add "to our knowledge" or similar.

We have made the recommended edit.

Please remove the "TM" symbol at line 199 and any other instances (including in the metadata).

We gave removed the TM symbol in the manuscript and metadata.

Please refer to the attachments (protocol, statistical analysis plan and CONSORT checklist) at suitable points in the methods section of your main text.

We have added the supplemental data references in places where this was deemed appropriate in the Methods and Results sections.

We suggest harmonizing the study start and end dates quoted in your abstract and results section.

The dates have been harmonized in the two sections.

Please note that the abstract in the metadata section is not correct and was reduced to accommodate the 500 word limit. Please kindly have editorial staff replace the text in the metadata with the abstract in the manuscript, which reflects peer review.

We have also responded to the production issues below:
FIGURES and GRAPHICS:

1. Please ensure that all main figure files are cited in ascending numerical order in the main text of the article.

We have done this and confirm this is the case.

2. Please ensure that all main figures are referenced in the manuscript as Fig 1, Fig 2, etc. (including capitalization), rather than Figure 1, Figure 2, etc. Please note, however, that the file names themselves must not include the space, i.e. Fig1.tif, Fig2.tif, etc.

We have Referenced the figures in the manuscript appropriately and the accompanying files are labeled as specified.

3. Please ensure that all main figure legends are placed after the paragraph containing their first in-text citation.

We have added the legends in the manuscript and removed the embedded figures.

4. Please remove embedded figures from your manuscript file and ensure the most up to date versions of your figures are uploaded to our submission system as separate TIF or EPS files. Please do not remove the figure legends and captions.

We have done this as above.

TABLES and BOXES:
1. Please cite main tables in ascending numerical order in your main article file.

We confirm that the main tables are cited in ascending numerical order.

FHI 360 HEADQUARTERS
359 Blackwell St, Suite 200
Durham, NC 27701 USA
T 1.919.544.7040
F 1.919.544.7261

WASHINGTON DC OFFICE
1825 Connecticut Ave
NW, Washington DC
20009 USA
T 1.202.884.8000
F 1.202.884.8400

ASIA-PACIFIC REGIONAL OFFICE
19th Floor, Tower 3,
130-132 Sindhorn Building
Wireless Rd, Lumpini, Phatumwan
Bangkok, 10330 Thailand
T 66.2.263.5200
F 66.2.263.2114

REGIONAL TECHNICAL HUB
Chancery Building, 2nd Floor
Valley Road
Nairobi, Kenya
T 254.20.271.3911
F 254.20.282.4171

2. Please ensure that all main tables in their entirety (including titles and footnotes), are placed after the paragraph containing their first in-text citation.

We confirm that the main tables are placed after the paragraph containing their in-text citation.

3. Tables 1-6 use returns to control text wrapping. This is indicated by a [¶ or ↵] symbol when "Show Paragraph Marks" is turned on in Word. Please either split those cells into separate cells to achieve alignment or remove the returns.

We have removed the returns in the attached manuscripts.

METADATA:
1. Please ensure that the funders and grant numbers match between the Financial Disclosure field and the Funding Information tab in your submission form. Note that the funders must be provided in the same order in both places as well.

We have checked this and confirm that the information matches in both places.

2. In accordance with PLOS' data policy, please ensure that your Data Availability Statement in the submission form clearly identifies how readers can access your data. Data cannot be available on request, or only accessible by contacting one of the authors. Note that your Data Availability Statement will be typeset as it is written, so please ensure it is in complete sentences and appears as you would like it to in the published version.

We have added the following description to access the data: "The de-identified database and variable specification files are available on DataVerse at the following link: https://dataverse.harvard.edu/dataset.xhtml?persistentId=doi:10.7910/DVN/NTN7KY"

3. At this time, you must make your data available via the repositories you mention. Please provide the direct link in your data availability statement.

Please see the above response.

ARTICLE FILE:
1. The name for your co-author Landon Myer does not match across the article file and the submission form. Please ensure that all author names match verbatim, including the use of a middle name/initials and any special characters (accents, umlauts, etc.), as correct spelling and formatting of author names is vital for accurate indexing in PubMed.

We have checked this and Dr. Myer's name appears as "Landon Myer" in both the article file and the submission form, if by submission form, you mean the information that shows up as "Manuscript Data" in the Editorial Manager. Please advise if there is a discrepancy we have not detected and please provide more explicit details. Please also advise why some of the authors' affiliations do not appear in the "Manuscript Data" section for Author in formation yet are entered in the individual description for each person.

2. Please remove the Competing Interests section from your manuscript file. The published version is pulled automatically from the "Competing Interests" field in the submission form and must not be duplicated. Please therefore take this opportunity to check that the information in this field is correct and completed in full.

We have removed this section from the manuscript and confirm the section in the metadata is correct.

3. Please ensure that your Ethics statement is available in the Methods section of your manuscript in its entirety.

The Ethics statement is provided in its entirety in the Methods section, inclusive of ethical review numbers. Please advise if there is concern that something is missing.

ACKNOWLEDGMENTS:
1. Please remove the funding information from the Acknowledgments section of your manuscript file. The published version is pulled automatically from the "Financial Disclosure" field in our submission form and must not be duplicated. Please therefore take this opportunity to check that the information in this field is correct and completed in full.

We have removed the funding information from the Acknowledgements section and confirm that the information in the Financial Disclosure section is correct. However, please advise whether the donation of kits and Mirena intrauterine systems belongs in the financial disclosure or funding section – it is currently in the funding section.

REFERENCES:
1. Please ensure your references are in the style of PLOS Medicine (ICMJE/Vancouver style). The reference style guide can be found here: http://journals.plos.org/plosmedicine/s/submission-guidelines#loc-references. You can also find a downloadable EndNote file in these guidelines to assist you. Please do a manual check as well, as the EndNote file does not catch everything.

Please note that the references will be typeset as they appear so please ensure the format is consistent.

We have reviewed the references and confirm they are in PLoS Medicine style.

2. Please put all reference citations in square brackets separated by either commas or dashes with no spaces, e.g. [1,2,3] or [1-3].

We have checked the manuscript and edited the reference citations to be compliant.

3. Please make sure all references are cited in ascending numerical order in the text.

We confirm all references are cited in numeric order.

SUPPORTING INFORMATION:
1. Please ensure your supporting information files are individually uploaded to the File Inventory as file type 'Supporting Information' and are not present in the manuscript file.

The Supporting Information files are individually uploaded as Supporting Information and are not present in the manuscript file.

2. Please upload your supporting information as individual files, and use the following naming format: S1 Text; S1 Fig; S1 Table, S1 Data, etc. Each file type should start as S1 and increase numerically: S1 Text, S2 Text, etc. Note that all relevant in-text citations and captions will need to be updated accordingly.

We have used the file naming system denoted in the below point and in the manuscript text.

3. Please ensure that all Supporting Information files use the following format verbatim throughout the manuscript (including legends and all in-text citations): S1 Fig, S1 Table, S1 Text, S1 Data, etc. Each file type should start at 1 and increase numerically. Please note, however, that the file names themselves must use an underscore rather than a space, i.e. S1_Fig.tif, S1_Table.xlsx, S1_Text.doc, S1_Data.xlsx, S1_CONSORT_Checklist.docx, etc.

The file names have been updated as directed.

4. Please ensure that each supporting information file has a legend at the end of the manuscript file, after the Reference list.

We have added legends for each of the supporting files at the end of the manuscript.

5. Please note that your submission cannot contain any references to generic "supporting information" files and instead must refer to specific files using our naming convention (S1 Fig, S1 Table, S1 Text, S1 Data, SI CONSORT Checklist, etc.)

We have named the files in the manner most consistent with instructions. Please advise if further editing is needed.

6. Please replace any page numbers in your CONSORT statement with paragraph numbers per section (e.g. "Methods, paragraph 1") since the page numbers of the final published paper may be different from the page numbers in the current manuscript.

All page numbers have been removed from the CONSORT checklist.

Thank you for your consideration and we look forward to your response.

Sincerely,

Catherine Todd

Catherine Todd

FHI 360 HEADQUARTERS
359 Blackwell St, Suite 200
Durham, NC 27701 USA
T 1.919.544.7040
F 1.919.544.7261

WASHINGTON DC OFFICE
1825 Connecticut Ave
NW, Washington DC
20009 USA
T 1.202.884.8000
F 1.202.884.8400

ASIA-PACIFIC REGIONAL OFFICE
19th Floor, Tower 3,
130-132 Sindhorn Building
Wireless Rd, Lumpini, Phatumwan
Bangkok, 10330 Thailand
T 66.2.263.5200
F 66.2.263.2114

REGIONAL TECHNICAL HUB
Chancery Building, 2nd Floor
Valley Road
Nairobi, Kenya
T 254.20.271.3911
F 254.20.282.4171
