# Supplementary material for: Safety and continued use of the levonorgestrel intrauterine system as compared with the copper intrauterine device among women living with HIV in South Africa: A randomized controlled trial
Source: PLoS Med. 2020 May 22;17(5):e1003110. doi: 10.1371/journal.pmed.1003110 (PMC7244096; doi:10.1371/journal.pmed.1003110)
Supplement: Attachment — Submitted filename: ResponseLetterTodd_2IUDnCT_24FEB20.docx [file pmed.1003110.s011.docx]

February 24, 2019

Richard Turner, PhD, Senior Editor

PLOS Medicine Special Issue: Maternal and Child Health & Nutrition

1160 Battery Street, Suite 225, San Francisco, CA 94111

plosone@plos.org

RE:	Revision of “Comparison of safety and continuation of the levonorgestrel intrauterine system versus the copper intrauterine device among women living with HIV in South Africa: a randomized controlled trial”

Dear Dr. Turner:

We thank you and the reviewers for the constructive review and respectfully submit our revised manuscript. Please find in the following text point-by-point responses to the critiques with the amended text and page number in the Track Changes version of the manuscript in normal font, preceded by the reviewer comments in italicized font. Please also kindly note that the abstract required revisions that cause it to exceed 500 words. We uploaded a shorter version into the online system to allow the system to process the revision submission. Please see the tracked and clean versions of the manuscript for the abstract responsive to editor and reviewer comments.

*Requests from the editors:*

*1. Please finalize the arrangements for data deposition.*

We will deposit the de-identified database with codebook and study instruments at a FHI 360 Harvard Dataverse web folder on acceptance of the manuscript. The weblink will be provided at the time of manuscript acceptance to update the metadata.

*2. If devices used in the study were donated by commercial entities, please state this in the financial disclosure (metadata).*

We have added the following information to the Financial Disclosure section of the metadata:

“We received in-kind donations from Bayer Pharmaceuticals (Mirena® LNG-IUS), the Western Cape Government (C-IUDs), Sekisui Diagnostics (a portion of Osom® BV Blue and Trichomonas), Cepheid Inc (a portion of Xpert CT/NG cartridges), and Alere-Abbott (Determine Syphilis RDTs).”

*3. Please add the start and end dates of participant recruitment to your abstract, and identify the primary endpoint early in the "methods and findings" subsection.*

We have added this information to lines 36 – 40 of the Methods and Findings section of the abstract in the Tracked version:

“*Between October 18, 2013 and December 31, 2017*, we randomized consenting women within ART groups, using 1:1 permuted block randomization stratified by ART use, age (18–23, 24–31, 32–40), and recent injectable progestin contraceptive (IPC) exposure, and provided the allocated IUC. At all visits, participants provided specimens for gVL (*primary outcome*), pVL, RTI, and pregnancy testing.”

*4. In the abstract, please add a few words to clarify "related SAEs".*

We have clarified what we mean by related SAEs in lines 60 - 62 of the Tracked version:

“There were 39 serious adverse events (SAEs); SAEs *believed directly related to IUC use* (n=7) comprised three pelvic inflammatory disease (PID) cases and four pregnancies with IUC in place with no discernible trend by IUC arm.”

*5. Please quote 1-2 further study limitations in the abstract.*

We have added two further limitations in lines 64-69 of the Methods and Findings section of the abstract in the Tracked version:

“Limitations included not achieving our planned non-ART group sample size due to new ART treatment guidelines during recruitment *and 17 women were not yet eligible for their 24-month visit at study closure, resulting in truncated 24-month visit outcome data. Also, the assay used for VL testing changed during the course of the study and may have caused some discrepancy in VL values as the assays have different limits of detection.*”

*6. In the "conclusions" subsection of your abstract, please adapt the wording of the first sentence to use the past tense, e.g., "In this study, we found that ... did not increase ... and had ...".*

We have changed the wording as advised in lines 70-72:

“*In this study, we found that* the LNG-IUS did not increase gVL or pVL and had low levels of contraceptive failure and associated PID *compared with the C-IUD among WLHIV*.”

*7. In the abstract and throughout the paper, please add p values alongside 95% CI where available.*

This information has been added in the abstract and throughout the paper.

*8. After your abstract, please add a new and accessible "author summary" section in non-identical prose. You may find it helpful to consult one or two recent research papers in PLOS Medicine to get a sense of the preferred style.*

We have added this section in lines 97 – 132 in the Tracked version.

*9. Please remove the final sentence of the introduction; you may wish to include this element of discussion later in the paper.*

We have removed this sentence and have moved it to lines 1022 - 1025 in the Discussion section of the Tracked version.

*10. Please remove the "role of the funding source" information from the text (this information will be included in the article metadata in the event of publication).*

This content has been removed.

*11. Throughout the ms, please quote p values as p<0.001 or exact values.*

We have made the requested change.

*12. Please remove trade marks, throughout the article.*

We have made the requested change.

*13. Please add additional access details to reference 22, as needed.*

Fpr what is now reference 30, we note that this particular journal does not use page numbers and we have added additional identifiers from the publisher’s website to line 1308 of the Tracked version.

*14. Please include a completed CONSORT checklist as a supplementary document, referred to in the methods section of your main text. In the checklist, individual items should be referred to by section (e.g., "Methods") and paragraph number rather than by page or line numbers, as the latter generally change in the event of publication.*

A CONSORT checklist has been completed and is included as S1 Checklist.

*Comments from the reviewers:*

**** Reviewer #1:*

*1) Abstract: it would helpful to report the allocation ratio here as well*

We have added this information to the Methods and Findings section in lines 36 - 40 in the Tracked version:

“*Between October 18, 2013 and December 31, 2017,* we randomized consenting women within ART groups, *using 1:1 permuted block randomization stratified by ART use, age (18–23, 24–31, 32–40), and recent injectable progestin contraceptive (IPC) exposure*, and provided the allocated IUC.”

*2) Introduction: From the introduction, it appears from the literature of other small observational there is some uncertainty in either LNG-IUS compared to C-IUD. I just want to understand the hypotheses better, that given some uncertainty of previous studies described in the introduction. Are the authors saying that null is no difference in primary outcomes with the alternative being "there is a difference between primary outcomes" - typical superiority design OR Are they suggesting that the null is treatments differ and the alternative hypothesis is the treatments are same within a tolerable margin? - non-inferiority design. It would good if the authors can clarify this.*

We thank the reviewer for raising this point and have clarified the Introduction to specify that the null is that there is no difference between LNG-IUS and C-IUD regarding change in genital tract viral load, hence a non-inferiority study. Please see the edited statement in lines 196 - 197:

“Our hypothesis was that the LNG-IUS was no different from C-IUD regarding change in gVL (non-inferiority trial).”

*3) Introduction: Last sentence - this should go in the discussion as a limitation instead of the introduction.*

We agree and have moved it to the Discussion, per response 9 to the Editor’s comments.

*4) Methods - statistical analysis: As the authors will probably appreciate, at-treated analysis is aimed to estimate the effect of the treatment "as delivered" or "as received" opposed to ITT "as assigned" to account for treatment non-adherence. Non-ITT or as-treated analysis usually presented as secondary analysis but in this paper it is presented the other way around. I can understand why this was done given the authors captured adherence parameters but I think the authors should give some rationalisation towards this decision in the analysis section to help understanding.*

The primary and several of the secondary outcomes were safety measures; as such, we believe that as-treated analysis provides the most accurate picture of the effect rather than intent to treat, which would have a more conservative bias that is not desirable for safety outcomes. Please see the Methods section, lines 351 - 356 for this additional rationale:

“We selected AT analysis during study design and analysis planning as the VL outcomes, pVL and gVL (the latter becoming the primary outcome measure), are both safety measures reflective of potential accelerated disease progression and transmission to partners, respectively. As safety outcomes, we believed it was essential to reflect actual exposure to IUCs with the AT approach, rather than the more conservative ITT. We conducted and present both AT and ITT results.”

*5) Methods -statistical analysis: Sentence beginning with "adjusted models for both ART groups..." referring to pre-specified confounders - can you refer to this in the pre-specified analysis plan.*

Yes, these confounders are mentioned in the analysis plan, which is attached as supplemental material.

*6) Methods - statistical analysis: Statement on participants with "undetectable" or lower than LoD results being assigned values...this needs some justification or reference associated with this decision.*

We have added the justification for this approach in lines 375 – 395 of Methods:

“*For analyses of VL as a continuous measure with log_10_ values, participants* with ‘undetectable’ or lower than LoD results were assigned the value of 20 copies/mL, representing the lowest LoD *of the two commercial assays used for VL measures and the mid-point between 0 and LoD of the assay with the higher LoD. Previous studies have used either approach (the manufacturer’s LoD to quantify undetectable measures [18, 32-34] or the value between 0 and the LoD for undetectable values [35-37]). As VL tests were run through the national laboratory and assays were changed mid-way through the study, we believe using the value of 20 copies/mL represents the best assigned value for undetectable VL measures.*”

*7) Methods - statistical analysis: On methods regarding Cox models for discontinuation - please indicate if these adjustment for covariates were pre-specified and proportional hazards assumptions were checked (i.e. Schoenfeld residuals).*

The adjustment by co-variates was pre-specified per response 5 and the proportional hazards assumptions were checked by Schoenfeld residuals, which do not reject the assumption of proportionality with p=0.134. We have added this information on lines 400 - 402 of the Analysis sub-section:

“We also estimated crude and adjusted hazard ratios of discontinuation, adjusting *for pre-specified co-variates of* age, ART status, and concurrent RTI, using Cox proportional hazards models. *The proportional hazard assumptions of Cox models were examined using Schoenfeld residuals, which do not reject that assumption with p=0.134*.”

*8) Results - CONSORT flow diagram: indicate in flow diagram during follow-up boxes in each arm that the numbers included for the primary analyses were analysed in as-treated or non-ITT analysis either as * or footnote.*

We have adjusted Fig. 1 to include an asterisk for participants retained in analysis with a legend at the bottom of the figure on p. 16 of the Tracked version.

*9) Results - statement on Page 14 regarding clinical activity closures - It's unclear what this means - is this study closure and the participants were unmasked, therefore they could not be eligible? Is this the correct interpretation?*

We thank the reviewer for raising this and have clarified the text to state that study activities closed prior to some of the last recruited participants being eligible for their 24 month visit. Thus, the participants weren’t eligible based on need to close the study clinical activities and were unmasked at an exit visit at time of site closure, rather than being ineligible due to being unmasked. Please see lines 425 – 451 in the Tracked version:

“Due to clinical activity closure in *July 2018*, *17 women (16 ART users, one non-ART user)* were not eligible for the 24-month visit; *as such, we did not conduct a 24 month visit with VL and RTI measures for these participants. Instead, to ensure informed contraceptive decision-making,* we conducted exit visits to unmask these participants *and ask if they wished to retain their IUC. We also did telephone follow-up with these participants at the time of 24 month visit eligibility to assess IUC retention in women who elected to continue their allocated IUC at exit visit.*”

**** Reviewer #2:*

*1. The manuscript would be strengthened by additional attention to the reason gVL was chosen as the primary outcome, the clinical significance of detectable gVL, and the significance of the lack of a difference in gVL for the question of HIV transmission among women using IUD.*

We selected gVL as a proxy measure for transmission risk as available literature links measurable female gVL to female to male transmission; these papers were available at the time of the decision to change the primary outcome measure in 2014.^[1]^ We posit, as others have,^1,^^[2]^ that detectable gVL has associated risk of transmission to male partners, particularly in this setting where women reporting ART use frequently had detectable pVL and gVL, with viral shedding potentially exacerbated by reproductive tract infections. We acknowledge that there is no defined “level” at which transmission is more likely and there is no standard, to our knowledge, of difference in viral load associated with increased risk of infection. At study conception, power calculations were based on the original primary outcome, pVL, where a difference of 0.5 log_10_ in VL was considered clinically relevant and we adopted the same standard for gVL. Changes in the text are conveyed in response 4, in which this reviewer has requested further information.

*2. An inclusion criterion was to be screened with cervical cytology within the last year. Given the poor prevalence of cervical ca screening in SA, how does this impact the generalizability of the findings?*

We do not believe this criterion impacts generalizability of the sample as we screened for recent (within the last year) cervical cytology reports at entry and if none were found, performed the smear during the screening visit to allow the interested woman the option for study entry. We have amended the Methods section to reflect that a cervical cytology report was required at enrollment and women without recent cervical cytology had Pap smears done at the screening visit. Please see the following text in lines 209 - 214 in Methods of the Tracked version:

“Eligible women were ages 18–40 years; confirmed as HIV-infected; not pregnant or desiring pregnancy for the next 30 months; at least six months postpartum; not planning relocation within 30 months; screened with cervical cytology within the last year (*women without a documented cervical cytology report within the last year were offered cytology smear during the pelvic examination at the screening visit in an opt-out fashion to ensure appropriate triage for evidence of neoplasia and eligibility at enrollment visit*); without history of ectopic pregnancy, tubal sterilization, or other conditions contraindicating IUC use; and interested in IUC use for contraception.”

*3. Another inclusion criterion was interest in IUC use for contraception. How unique is this group?*

We conducted a cross-sectional assessment to measure interest in potential IUC use among a similar population in the Cape Town area, which suggested high (86%) levels of interest and receptivity among young WLHIV.^[3]^ However, when faced with actually entering a trial and receiving the IUC, we found less interest. That said, we noted distinct differences in non-ART vs. ART using women as we were able to fill the ART group sample size within 15 months, whereas the non-ART group did not fill. We attribute this mainly to there being fewer ART-naïve women as ART guidelines became more inclusive. However, we also note that the leading reason for ineligibility among non-ART women was very low CD4 count and the clinic where study clinical activities were conducted was known for having studies only open to WLHIV. Thus, it is our conjecture that non-ART women who feared public knowledge of their status may have avoided the study clinic similar to apparent lapses in HIV monitoring and care among many in this group. We have added the following sentences to the limitations section of the Discussion to relay this theory in lines 1166 – 1171 in the Tracked version:

“We also note that among non-ART women screened, many were not eligible due to low CD4 lymphocyte counts constituting ART eligibility by guidelines in effect at study start in 2013. We hypothesize that some non-ART WLHIV preferred not to disclose their status and hence avoided any clinical setting related to HIV care, including the clinic where this study’s clinical activities were conducted.”

*4. Please provide additional information about why detectable gVL was chosen as an outcome and what is known about what absolute levels are required for efficient transmission. There are a lot of data re. genital HIV shedding in the presence of plasma HIV-RNA suppression - and yet the data re plasma undetectable = uninfectious are compelling and now accepted by most as "trumping" information re. genital VL. What are the data that suggest that gVL matters for this question? And what were the cut-offs and what data inform that? And for power, what is the meaning of a 19% difference in detectable gVL? And why is the assumed pooled baseline proportion or women with detectable gVL estimated to be 42.8%? Overall, given that gVL is the primary outcome, it would be helpful to say a bit more about this measure and the existing data that should inform interpretation of these data in the intro/discussion.*

In addition to the response provided to this reviewer’s first critique, we add that while an absolute gVL level for efficient transmission is not known and the risk of female to male heterosexual transmission is low as modeled among untreated populations,^[4]^ there is consensus that viral shedding does occur even in the presence of plasma viral suppression and thus risk of transmission is still theoretically possible. One review has found no events of heterosexual transmission events where the infected partner had pVL<400 copies/mL, while another concluded that transmission risk where the infected partner had undetectable pVL was very low but added the caveat that multiple factors, including RTIs, may modify this relationship.^[5]^ In our cohort, pVL values frequently exceeded 400 copies/mL and often 1000 copies/mL among women using ART, indicating that transmission risk with typical ART use in this context requires concern and our findings suggest it is unlikely to be exacerbated by use of either IUC. Further, a recent commentary co-authored by the lead author of the HPTN 052 trial notes the paucity of data surrounding reasons for and implications for infectivity with persistent gVL in the presence of viral suppression and questions whether “U still =U” in the presence of RTIs.^[6]^ In light of this mixed picture and the context of frequent episodes of detectable pVL in our study population, we have added the following text to lines 154 - 156 in the Introduction:

“*Genital tract viral load (gVL) has been implicated in HIV transmission to male partners and a host of factors are noted to impact gVL, potentially including exogenous progestins* [12-14].”

And lines 176 – 184:

“*Whether IUCs are safe for use among WLHIV and, specifically, whether IUCs impact gVL are germane given expanded global ART use and findings of persistent detectable gVL among WLHIV virally suppressed with very low to undetectable plasma viral load (pVL) [21-23]. There are emerging concerns that HIV acquisition and transmission risk may increase in the presence of reproductive tract infections (RTIs), non-Lactobacillus dominant microbiota, and hormonal contraceptives due to local interaction with ART agents [24-26]. Thus, evidence regarding gVL change with exposure to hormonal and non-hormonal IUC use among WLHIV with typical ART use patterns in a context with high background RTI prevalence is timely and may help inform this debate.”*

And to lines 1026 – 1033 in the Discussion to frame our choice of gVL as a relevant primary outcome vis a vis IUC safety among WLHIV:

“*We selected gVL as a proxy measure for transmission risk given association between gVL and sexual transmission to male partners in discordant couples [12,38]. There are limited data regarding quantity at which gVL substantively increases transmission risk, though one publication suggests transmission is unlikely below 400 copies/mL in ART-using populations [39].* Our safety findings *for odds* *of having detectable gVL* did not change *with exposure to either IUC or between study arms* across six and 24 months even after adjusting for ART use, RTI, and pVL, and in sensitivity analyses adjusted for disproportionate IUC discontinuation between arms.”

And in lines 1057 – 1069:

“There was no significant detectable effect of levonorgestrel from the LNG-IUS on pVL measures between study arms within each ART group, consistent with previous studies [10,11]. Additionally, we found that more than 15% of ART users had pVL>1000 copies/mL at any visit after enrollment, consistent with trends attributed to ART non-adherence in Cape Town *and the association of detectable gVL with detectable pVL in other studies assessing impact of contraception on genital tract shedding [34, 37, 40, 42]. This finding* was associated with increased proportions of women with detectable gVL, *irrespective of IUC type and potentially augmented by RTI incidence. Given frequent pVL levels that reflect lack of clinical viral suppression and thus increased probability of transmission among our participants and thus reflective of “real world ART use”, that neither IUC increased detectable gVL is clinically important.* Further, these pVL trends emphasize the need to ensure WLHIV are counseled *regarding condom use* to prevent transmission to partners when their HIV is not fully suppressed*.*”

We have provided information about cut-offs and supporting evidence in response 6 to Reviewer 1. Regarding the pooled assumed shedding value, this proportion was determined at the time of changing gVL to the primary outcome measure and adding the ART-user group in 2015. We calculated the shedding assumption by using the measured proportion of non-ART participants enrolled by July 2015 with detectable gVL (61%; n=39) and the reported proportion of women using ART with detectable gVL from an African cohort^[7]^ (pooled sample size estimates 116 (62 + 54) evaluable subjects per arm with an estimated presence of vial shedding in C-IUD recipients of 42.8% (61% * 62/116 + 22% * 54/116)). We have provided this information in the Methods section, lines 322 - 324:

“The pooled gVL value was calculated by using non-ART gVL of 61% * 62/116 + estimated ART-user gVL of 22% * 54/116); this was calculated in 2015 at the time of primary outcome change and ART group addition with available evidence [22].”

*5. Methods state, "Due to clinical activity closure, 19 women (17 ART users, two non-ART users) were not eligible for the 24-month visit." Please clarify what this means.*

Please see response 5 to the Editor’s queries and response 9 to Reviewer 1 for clarification and correction of the numbers.

*6. An important point for methods and discussion - were women exited when they discontinued IUD? If so, that would likely alter (increase) continuation rates given 2dary benefits of being in a clinical trial in RSA.*

Women were not exited from the study if they elected to discontinue the allocated IUC to avoid the identified bias as well as have data for change in gVL with IUC removal. We have clarified this in lines 268 – 270 in the Methods section:

“*Women requesting IUC removal and agreeing to continue with the study were retained in the study through the 24 month visit but excluded from all as-treated analyses; the only participants terminated from the study were those with diagnosed pregnancy.*”

*7. An important point that is missing from the current version of the discussion is that 40% of women had a curable RTI which may not have been picked up via standard of care syndromic screening. Contraceptive programs should be supported by MOH to implement testing protocols.*

We agree and have raised this point in the Discussion in lines 1020 – 1022:

“*There were few cases of PID and the IUC had a high overall continuation rate, which is notable among a population with high background rate of curable RTI.*”

And lines 1070 – 1100:

“We found low numbers of related SAEs, particularly PID, despite relatively high RTI prevalence within our cohort. There were three PID cases, occurring in both arms and at levels similar to those reported *by studies with comparator groups in some contexts [8,43] but higher than in others [17,44,45].* *The cohorts with lower rates were European WLHIV comprising mainly of experienced ART users with no reported PID cases [14], a postpartum Zambia cohort recruited prior to Option B+ ART coverage [44], and a Ugandan cohort [45]. For the Zambian study, we believe recruiting postpartum women with no history of PID reduced PID risk due to possible reduced sexual activity and condom use and thus lower exposure to pathogens, evidenced by only one case of PID within that cohort of 296 women contributing 642 person-years (0.16/100 p-y) of follow-up [44]. The Ugandan cohort reported five cases (2 LNG-IUS and 3 C-IUD) of PID among 672 participants (incidence rate ratio=0.7, 95% CI: 0.06 – 6.04) with all cases diagnosed and managed by the study site [45]. Our three cases include two that were diagnosed and managed at other clinical sites and it is possible that IUC use may have predisposed the provider to PID diagnosis [46,47]. RTIs were measured, detected and treated at screening (prevalence data), continued to occur through the cohort period and were largely asymptomatic with few findings on exam [48]. Given the high prevalence of asymptomatic infections, RTI testing using point-of-care tests rather than syndromic management, the standard of care, should be added within IUC insertion guidelines to reduce risk of PID specifically related to IUC use and thus address some provider concerns that likely hamper offering IUCs to WLHIV [20,49].* ”

*8. Table 7 the back-slash (/) before p-value is odd looking in table. This can be managed in journal formatting.*

We have removed the back-slashes in the former Table 7, now Table 6.

*9. Consort Figure 1 - The reason for 21 of those excluded from the pre-ART arm is "other". Could you describe since this is 10% of those screened?*

We have revised the reasons for exclusion in Fig 1 to more accurately reflect reasons for ineligibility/exclusion. Please see the revised Fig. 1 on p. 16.

*** Reviewer #3:

*1. It is unclear in Figure 1 whether the 6 women who declined, were ineligible, or who could not have the IUD inserted are post-randomization. If so, these women should be included in the ITT analysis.*

We did not conduct any follow-up on women who declined or could not undergo IUC placement, regardless if this occurred after randomization. As such, these women are not included in the ITT analysis – they never received either IUC and did not come back to the clinic so do not have any outcome data.

*2. Table 2 is dense and I found it difficult to interpret. The reader is left to calculate the "deltas" on his/her own. Would it not work better to split it into a figure for the visit trends and then report the 6- and 24-month deltas in a separate table? I'm not really sure, but Table 2 seems to bury your primary results. Also, it is confusing how the "% (n)" designation in table 2 differs from table 1, which uses "n (%)."*

We agree and have converted these comparisons to graphic representation. Please see the new Fig. 2 “Trends in female genital tract and plasma viral load and intrauterine contraceptive continuation by study arm and within ART use strata across the cohort period overall and by as-treated women living with HIV” on p. 19.

*3. While there was no difference observed between the two methods with respect to genital HIV shedding, it might be informative to the reader to provide comparative data from HIV infected women on other methods. I found myself wondering whilst reading the discussion whether I should be concerned that 10-30% of women on ART had detectable virus in their vaginal samples. How does this compare to other contraceptive methods?*

We thank the reviewer for this comment and have added further information from other studies that assessed viral shedding in presence of depot medroxyprogesterone acetate and the levonorgestrel implant in the Discussion in lines 1033 - 1056:

“Chinula *et al.* found reduced proportions of women with detectable gVL following LNG implant use at six months for tear flow strip samples (17.7% and 3.2% at baseline vs. 6.1% at 180 days; adjusted relative risk (aRR)=0.40, (95% CI: 0.18 – 0.85) adjusted for baseline pVL and CD4 count), compared to no significant change in women using depot medroxyprogesterone acetate (DMPA; 12.1% and 10.7% at baseline vs. 10.3% at 180 days, aRR=1.37 (95% CI: 0.81 – 2.33)) among WLHIV reporting ART use in Malawi [40]. For the same cohort followed up to a maximum of 33 months, Kourtis *et al.* found that risk of detectable gVL did not differ significantly over time after initiation of either method (aRR, 0.97, 95% CI: 0.84 - 1.13, per increase of 6 months of follow-up) or between the DMPA study arm compared with the LNG implant study arm (aRR, 1.92, 95% CI: 0.97 - 3.79) [37]. In this extended cohort period, detectable gVL also not significantly differ before method initiation compared with follow-up visits (p= 0.47). In a prospective cohort of WLHIV initiating ART measuring detectable gVL, defined as >400 copies/mL from endocervical swab, 30.3% of women reporting ART use had detectable gVL at one or more visits; and DMPA (OR 0.96, 95% CI 0.44–2.13) exposure was not associated with detectable gVL [34]. In a Burkina Faso cohort of women with high background rates of RTI, 45% (77/170) women had detectable gVL at one or more visits despite ART use with undetectable pVL [22]. In this cohort, neither reported oral contraceptive (AOR= 1.57, 95% CI: 0.75 - 3.27) nor DMPA use (AOR= 1.32, 95% CI: 0.42 - 4.16) were associated with detectable gVL in analysis adjusted for pVL. Our findings bear distinction from a mucosal immunology standpoint as the LNG-IUS has markedly higher LNG concentrations in genital tract mucosa, reflecting its largely localized effect, in contrast to the LNG implant and DMPA [41]. When compared to the C-IUD, LNG does not appear to drive shedding via gVL; neither IUC appears to increase gVL through hypothesized inflammatory response from foreign body effect.”

*4. The frequency of PID in this study, while low, is higher than that reported from analogous trials in Zambia (not referenced, but probably should be) and Uganda (ref #26). It might be helpful to report this outcome as a rate (events/person-years of follow-up) to allow comparison with other research.*

We agree and have described calculating SAE rates in lines 406 - 407:

“We describe SAEs deemed related to IUC use by arm *and with rates based on time contributed in an as-treated model.*”

And have reported PID as a rate in the Results section, lines 912 – 920:

“There were 39 SAEs, including two deaths unrelated to IUC use (LNG-IUS: smoke inhalation; C-IUD: brief illness disclosed by family), three cases of PID (two LNG-IUS *(rate= 1.2/100 person years or p-y, 95% CI 0.3 – 4.6),* one C-IUD *(rate= 0.7/100 p-y, 95% CI 0.1 – 4.9, p=0.68; overall rate= 0.9/ 100 p-y, 95% CI 0.3 – 2.9),* and four pregnancies with IUC in place (two ectopic pregnancies in C-IUD users; one incomplete abortion with no IUC recovered in a C-IUD user, suggesting unrecognized expulsion; and one intrauterine pregnancy in an LNG-IUS user). *Pregnancy rates with IUC in place were 1.3/100 p-y overall (95% CI 0.40 – 3.3), with 2.1/100 p-y (95% CI 0.70 – 6.50) for C-IUD and 0.6/ 100 p-y (95% CI 0.10 – 4.60) for LNG-IUS (p=0.26).*”

We have also considered the rate for PID and pregnancy in our cohort relative to similar trials and have added text to the Discussion section as noted in response 7 to Reviewer 2.

*5. The pregnancy rate also seems higher than expected. The results might benefit from conversion to rates and the discussion from a few point estimates from other sources for comparison (even if only available in HIV- women). In fact, for each of the important events: continuation, expulsion, pregnancy, PID - it would be helpful to have these reported as rates so that the reader could compare to other papers.*

Please see the response 7 to Reviewer 2 and the above response where we have added rates for PID and pregnancy. We also added rates for expulsion in lines 981 - 985:

“IUC continuation was 78% (155/199) across all participants during the cohort period; the overall IUC expulsion rate was 2.8/100 p-y, 95% CI 1.5 – 5.5. Expulsion (5.6/100 p-y, 95% CI 2.8 – 11.1) for C-IUD vs 0.6/100 p-y (95% CI 0.10 – 4.10) for LNG-IUS, p=0.03) and all-cause discontinuation rates (35% for C-IUD vs 6% for LNG-IUS, p<=0.001; Fig 3) were significantly higher among women using the C-IUD.”

In the Discussion, we have added further context for PID rates as above and also include additional context for pregnancy and expulsion in lines 1100 - 1109:

“There were two ectopic pregnancies, both occurring with the C-IUD, a propensity noted previously and possibly magnified in this cohort due to high RTI prevalence with tubal scarring [50]. The overall pregnancy rate was higher than expected based on some studies which found pregnancy rates between 0.06 - 1.7/100 p-y [45,50,51] but lower than the 3-year cumulative rate for C-IUD users noted in a large multi-center cohort of 2.8/100 p-y (95% CI: 1.3 – 6.0) [43]. We attribute some of the pregnancies and expulsions to IUC malposition, which may have been clinically unrecognizable until the IUC was visible at the cervical os; Bahamondes *et al.* noted similar rates among C-IUD users which they attributed to issues related to variability in inserter design [43].”

*6. I don't understand why 19 women were "not eligible" for their final visit.*

Please see response 5 to the Editor.

*7. There's a statement on pp 25-26 that says: "Among women discontinuing their allocated IUC, median time to elective discontinuation was significantly shorter for C-IUD users (7.2 months for C-IUD vs 11.2 months for LNG-IUS; Fig 2)." It doesn't seem appropriate (or necessary) to report this rate among women who discontinued their method, since it would no longer be a randomized comparison. One way to report this and maintain the randomization would be to right censor those who continue at 24 months (like Figure 2 does) and report median time on contraception. If you do decide it is important to include this calculation, I would advise not referring to Figure 2, since that KM appears to include all women.*

We note the reviewer’s concern and have removed the reference to the new Fig 3 and the median time to discontinuation.

8. *CONSORT calls for the discussion to address sources of "potential bias, imprecision, and, if relevant, multiplicity of analyses." The imprecision issue seems particularly relevant here. For instance, the proportion of women on ART with detectable gVL was increased nearly 3-fold between randomization and 24 months in the cIUD group, compared to very little change in the LNG-IUG group. With a larger sample, would we be worried about this?*

Since the participants were randomized into treatment arms, bias should be minimal in expectation at the beginning of the study. However differential loss to follow-up by treatment arm, if related to risk of the primary outcome (gVL), could bias the results. While the C-IUD group experienced higher dropout, this was related to device expulsion and side effects, which seem unlikely to be related to gVL. Further, sensitivity analyses using inverse probability censoring weights to adjust for differential censoring yielded similar findings. While multiplicity of analyses is a potential problem, the issue here is that with many analyses, one will have a false positive finding by artifact. However, none of our analyses had a positive finding in the direction of the alternative hypothesis of greater viral replication with the LNG-IUD. In fact, the finding being noted of three-fold increase in the viral load in the C-IUD compared to no change in the LNG-IUD is in the opposite direction of the alternative hypothesis. Thus, while imprecision is an issue, in addition to the lack of statistical significance for the association, that it is directionally counter to the expectation under the alternative is further reassuring.

9. *Finally, while the trial analysis follows that which is outlined in the protocol, it is unclear why the authors opted for an adjusted analysis as primary. The randomization procedures described were convincing and I found myself most interested in the unadjusted ITT analysis. Perhaps the pooling approach mandates this, but I think most clinical readers will search for unadjusted head-to-head comparisons.*

Regarding selection of A-T, please see response 4 to Reviewer 1. We do provide results for both A-T and ITT analyses for the VL outcome measures.

For the ITT and AT analyses, we adjusted for baseline detectable gVL, as the primary outcome was the change in gVL post IUC insertion and the baseline measure is the one measure that occurred prior to insertion. We further adjusted for age and ART group as required when using stratified randomization. We have clarified this in the Methods section as follows in lines 361 - 364:

“All models were adjusted for baseline gVL, as the primary outcome was change in gVL, post-IUC insertion, and adjusted for age and ART group, given stratified randomization. In sensitivity analysis, we further adjusted AT models for pre-specified hypothesized confounders of concurrent RTI and baseline pVL”

Thank you for your consideration and we look forward to your response.

Sincerely,

Catherine Todd

1. Baeten JM, et al. Genital HIV-1 RNA predicts risk of heterosexual HIV-1 transmission. Sci Transl Med. 2011;3(77):77ra29; Mugo NR, et al. Increased risk of HIV-1 transmission in pregnancy: a prospective study among African HIV-1-serodiscordant couples. AIDS. 2011;25(15):1887-95. doi: 10.1097/QAD.0b013e32834a9338; Quinn TC, et al. Viral load and heterosexual transmission of human immunodeficiency virus type 1. Rakai Project Study Group. N Engl J Med. 2000 Mar 30;342(13):921-9.
2. Polis CB et al. Hormonal contraceptive use and female-to-male HIV transmission: a systematic review of the epidemiologic evidence. AIDS. 2013;27(4):493-505. doi: 10.1097/QAD.0b013e32835ad539.
3. Todd CS, et al. Awareness and interest in intrauterine contraceptive device use among HIV-positive women in Cape Town, South Africa. Infect Dis Obstet Gynecol. 2012;2012:956145. doi: 10.1155/2012/956145.
4. Powers KA, et al. Rethinking the heterosexual infectivity of HIV-1: a systematic review and meta-analysis. Lancet Infect Dis. 2008;8(9):553-63. doi: 10.1016/S1473-3099(08)70156-7; Baggaley RF, et al. Heterosexual HIV-1 infectiousness and antiretroviral use: systematic review of prospective studies of discordant couples. Epidemiology. 2013;24(1):110-21. doi: 10.1097/EDE.0b013e318276cad7; Boily MC, et al. Heterosexual risk of HIV-1 infection per sexual act: systematic review and meta-analysis of observational studies. Lancet Infect Dis. 2009;9(2):118-29. doi: 10.1016/S1473-3099(09)70021-0.
5. Attia S, et al. Sexual transmission of HIV according to viral load and antiretroviral therapy: systematic review and meta-analysis. AIDS. 2009;23(11):1397-404. doi: 10.1097/QAD.0b013e32832b7dca; Loutfy MR, et al. Systematic review of HIV transmission between heterosexual serodiscordant couples where the HIV-positive partner is fully suppressed on antiretroviral therapy. PLoS One. 2013;8(2):e55747. doi: 10.1371/journal.pone.0055747.
6. Cohen MS, et al. Sexually transmitted infections and HIV in the era of antiretroviral treatment and prevention: the biologic basis for epidemiologic synergy. J Int AIDS Soc. 2019;22 Suppl 6:e25355. doi: 10.1002/jia2.25355.
7. Low AJ, et al; Yerelon Study Group. Cervicovaginal HIV-1 shedding in women taking antiretroviral therapy in Burkina Faso: a longitudinal study. J Acquir Immune Defic Syndr 2014; 65: 237–45
